# 6-Chlorocoumarin Conjugates with Nucleobases and Nucleosides as Potent Anti-Hepatitis C Virus Agents

**DOI:** 10.3390/molecules30081776

**Published:** 2025-04-15

**Authors:** Shu-Yu Lin, Wen-Chieh Huang, Shwu-Chen Tsay, Johan Neyts, Pieter Leyssen, Chun-Cheng Lin, Kuo Chu Hwang, Jia-Cherng Horng, Jih Ru Hwu

**Affiliations:** 1 Institute of Biotechnology and Pharmaceutical Research, National Health Research Institutes, Miaoli County 350401, Taiwan; wchuang@nhri.edu.tw; 2 Department of Chemistry and Frontier Research Center on Fundamental & Applied Sciences of Matters, National Tsing Hua University, Hsinchu 300044, Taiwan; cclin66@mx.nthu.edu.tw (C.-C.L.); kchwang@mx.nthu.edu.tw (K.C.H.); jchorng@mx.nthu.edu.tw (J.-C.H.); 3 Rega Institute for Medical Research, Katholieke Universiteit Leuven, Minderbroedersstraat 10, B-3000 Leuven, Belgium; johan.neyts@kuleuven.be (J.N.); pieter.leyssen@kuleuven.be (P.L.)

**Keywords:** mercaptopurine, coumarin, nucleosides, hepatitis C virus, structure–activity relationship

## Abstract

On the basis of a “chemo-combination strategy”, (6-chloro)coumarin was incorporated to purines and pyrimidines, as well as their corresponding nucleosides, with a –SCH_2_– linker at different positions under alkaline conditions. These conjugates were found to exert an antiviral effect on the 1b subgenomic replicon replication of the hepatitis C virus (HCV) in Huh 5-2 and Huh 9-13 cells. In this compound library containing 14 new compounds, 6-[(6′-chlorocoumarin-3′-yl)methylthio]purine, 6-(6′-chlorocoumarin-3′-yl)methylthio-9-(β-D-ribofuranos-1″-yl)purine, and 2-[(6′-chlorocoumarin-3′-yl)methylthio]uracil showed great inhibitory abilities, with EC_50_ values between 6.6 and 9.4 μM and selectivity indexes >16–41. Moreover, the structure–activity relationship between purines and pyrimidines is elucidated, which reveals the critical factor of the attachment of the coumarin moiety at different positions in purines and pyrimidines.

## 1. Introduction

The hepatitis C virus (HCV) primarily influences the liver and is an infectious disease that is observed worldwide [1]. According to the World Health Organization, over 50 million individuals are chronically infected with HCV, and around 200,000 deaths each year are attributed to HCV-induced cirrhosis and liver cancer [2]. In the absence of a vaccine [3], treatment relies on direct-acting antiviral (DAA) agents [2]. A variety of DAA agents have been developed for the treatment of HCV, with FDA approval having been granted to prominent examples such as sofosbuvir and velpatasvir [2]. However, resistance-associated substitutions (RASs) have been reported with the use of these agents [4,5]. Additionally, their activity was restricted to certain genotypes and was linked to a notably high incidence of side effects [6]. The emergence of combination therapies that use drugs with different mechanisms of action addresses the issue of drug resistance in HCV treatment. Advancing treatments for the hepatitis C requires the discovery of drugs with mechanisms that are distinct from those which are already established. Consequently, the discovery of new chemical entities which can be used as drugs with high potency and selectivity remains as one of the top themes to be explored [7,8,9].

Various types of nucleoside analogues are being developed as potential treatments for HCV. By targeting the catalytic site of the RNA-dependent RNA polymerase, several nucleosides that are effective across all known genotypes and subtypes have progressed to clinical trials [10]. In addition, compounds from diverse families, such as benzimidazole, bromophenol, coumarin, and polyamide, are under investigation as promising NS3·4A protease inhibitors for HCV [11,12,13,14]. Their potency and selectivity, however, have been hampered due to the shallow and flat protease grooves that they possess for substrate binding [15,16]. 6-mercaptopurine (6-MP) analogs are prominent examples, which show significant inhibition of nucleoside transport proteins [17], as evaluated against *Toxoplasma gondii* adenosine kinase [18]. Moreover, these 6-MP derivatives exhibit antiviral activity against pathogens including respiratory syncytial virus (RSV), Middle East respiratory syndrome coronavirus (MERS-CoV), and Zika virus (ZIKV) [19,20,21]. Azathiopurine, a 6-MP prodrug, exhibits in vitro activity against bovine viral diarrhea virus (BVDV) and HCV, as reported by Hoover and Striker [22]. It has also been found that 6-mercaptopurine and 6-mercaptopurine ribonucleoside (6-MPR) can be used as anti-leukemic drugs [23,24,25].

As far as we are aware, there are few systematic studies on the development of conjugated compounds as anti-viral drugs through the incorporation of a designed moiety to nucleosides at various positions [26,27]. It is our plan to establish a strategic platform for such a study by synthesizing new HCV replication inhibitors [28,29,30,31], in which a coumarin moiety is connected at various positions of purines, pyrimidines, and their corresponding nucleosides. Its attachment at the C8 position of purines lead to so-called “waist-type” conjugates (e.g., **3a**,**b** and **5a**,**b**); at the C6 position of purines and the C4 position of pyrimidines lead to “head-type” conjugates (e.g., **7a**,**b**, **9a**,**b**, **11a**,**b**, and **16a**,**b**), and at the C2 position of pyrimidines lead to “tail-type” conjugates (e.g., **18** and **20**).

Here, we report an update on the establishment of a new compound library that includes 14 conjugated compounds. Among these conjugates obtained by chemical synthesis, three exhibited remarkable anti-HCV activity with high selective index values. The structure–activity relationship (SAR) among the 14 compounds is also deduced.

## 2. Results

### 2.1. Syntheses of Coumarin-Conjugated Nucleobases and Nucleosides

**Scheme 1:**
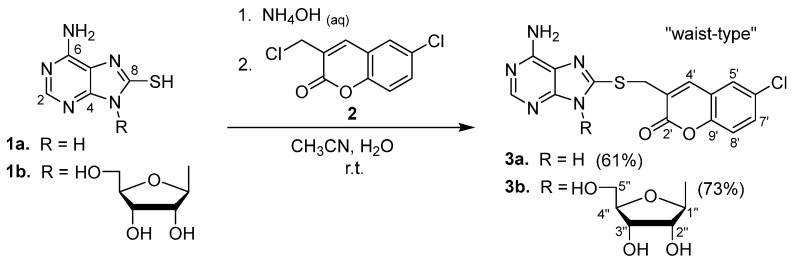
Synthesis of “waist-type” coumarin–thioadenine/thioadenosine conjugates.

**Scheme 2:**
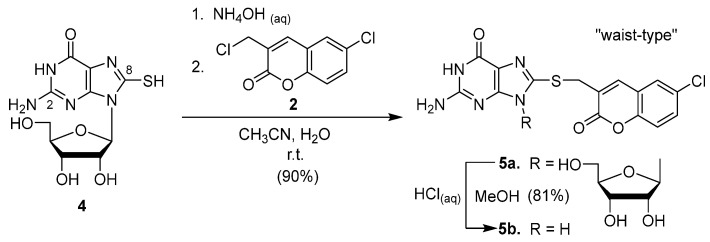
Synthesis of “waist-type” coumarin–thioguanine/thioguanosine conjugates.

**Scheme 3:**
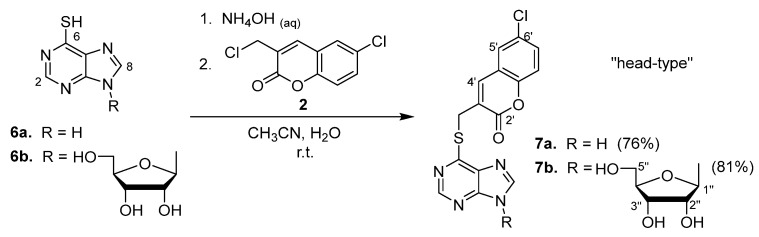
Synthesis of “head-type” coumarin–thiopurine/thiopurine nucleoside conjugates.

**Scheme 4:**
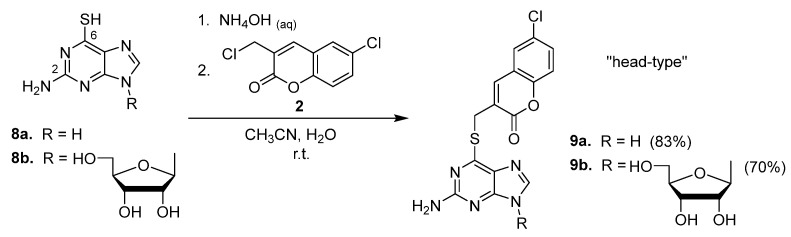
Synthesis of “head-type” coumarin–thiopurine/thiopurine nucleoside conjugates.

**Scheme 5:**
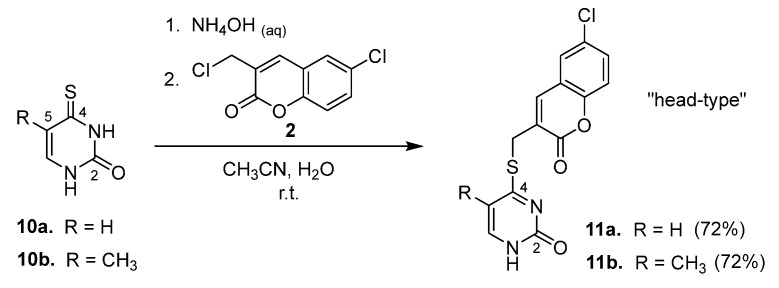
Synthesis of “head-type” coumarin–thiouracil conjugates.

**Scheme 6:**
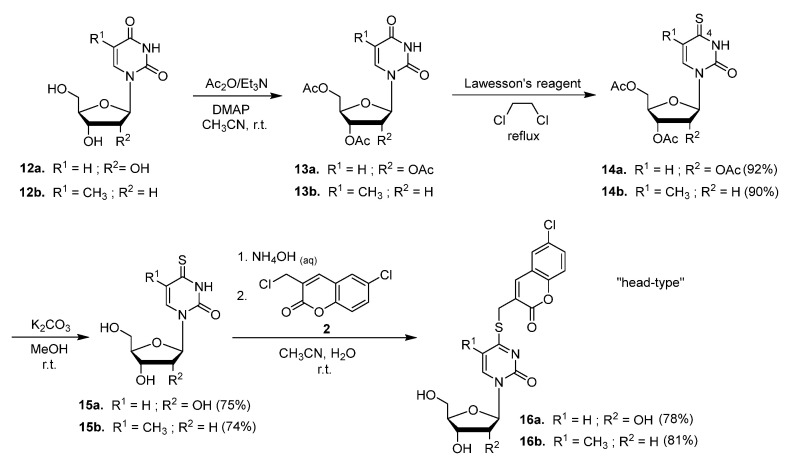
Synthesis of “head-type” coumarin–thiopyrimidine/thiopyrimidine nucleoside conjugates.

**Scheme 7:**
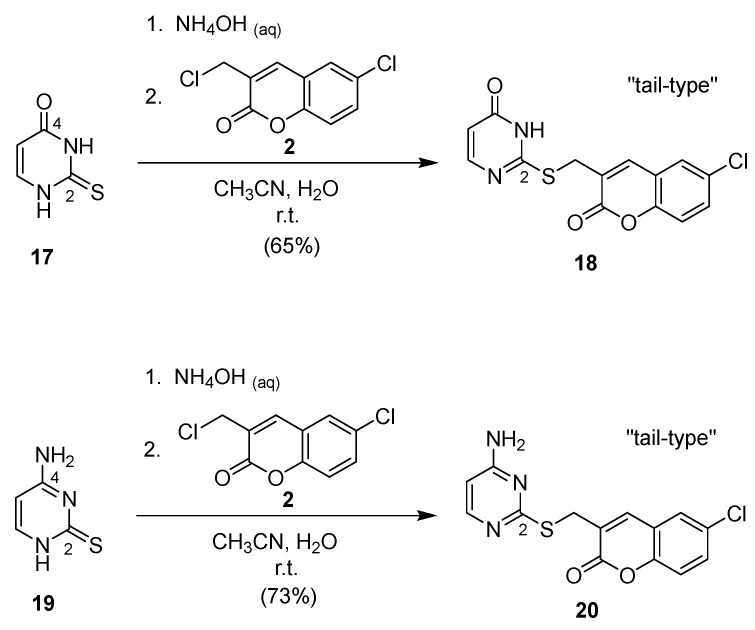
Synthesis of “tail-type” coumarin–thiopyrimidine conjugates.

Based on our previous work with imidazole-coumarin conjugates against HCV [32], we discovered that a 6-chloro substituent on the coumarin ring offers an improved balance between the inhibitory activity and selectivity index. Our studies comparing different substituents at the 6 and 8 positions confirmed that the 6-chloro substituent generally provides the optimal profile in imidazole-coumarin analogues. In the present study, we fixed the coumarin moiety with a 6-chloro group to evaluate the effects of various nucleobase conjugates on the resulting anti-HCV activity. Specifically, 3-(chloromethyl)coumarin **2** was selected to be coupled with purines, pyrimidines, and their corresponding nucleosides at different positions, enabling a systematic exploration of their structure–activity relationships in relation to their function as inhibitors of HCV replication. As shown in Scheme 1, Scheme 2, Scheme 3, Scheme 4, Scheme 5, Scheme 6 and Scheme 7, all of the conjugated products shared a common feature with a –SCH_2_– linker [33,34,35]. Thus, the pre-installation of a mercapto or thione group in the starting materials was necessary and the couplings were achieved by reaction of the coumarin **2** with various nucleobases and nucleosides in the presence of NH_4_OH_(aq)_ and acetonitrile at room temperature.

For the production of the “waist-type” conjugates, (8-mercapto)adenine (**1a**) and (8-mercapto)adenosine (**1b**) were treated with coumarin **2** (Scheme 1) [36]. Under the mild conditions that were employed, the C6-amino group did not restrain the C8-mercapto group from its alkylation to a significant extent. Consequently, the desired conjugates **3a** and **3b** were produced in 61% and 73% yields, respectively. The “waist-type” conjugates could also be generated by the coupling of (8-mercapto)guanosine (**4**) with coumarin **2**. As shown in Scheme 2, the desired coumarin–guanosine ribonucleoside **5a** was obtained in an excellent yield (90%). Apparently, the presence of three hydroxyl groups and a tautomerizable amido group in compound **4** did not efficiently impede the alkylation at the desired mercapto center. Furthermore, the ribosyl moiety in **5a** was removed successfully with aqueous hydrochloride in methanol to give a yield of 81% of the coumarin–guanine conjugate **5b**.

In contrast to the “waist-type” conjugates (i.e., **3a**,**b** and **5a**,**b**) resulting from adenine and guanine derivatives, shown in Scheme 1 and Scheme 2, the “head-type” conjugates **7a**,**b** were synthesized successfully by using the purine derivatives **6a**,**b** as the starting materials (see Scheme 3). Compounds **6a**,**b** possess a mercapto group at the C6 head position (cf. at the C8 waist position in **1a**,**b**). Moreover, 6-thioguanine (**8a**) and its corresponding nucleoside **8b** were converted in good yields to the “head-type” conjugates **9a** and **9b**, respectively, during which the C2-amino group remained intact (see Scheme 4). In addition to the purine derivatives, “head-type” conjugates with a pyrimidine moiety were also prepared (see Scheme 5). The treatment of 4-thiouracil (**10a**) and 4-thiothymine (**10b**) [37] with coumarin **2** yielded the corresponding conjugates **11a** and **11b**, respectively.

For the production of the “head-type” pyrimidine nucleosides, all hydroxyl groups in the uridine (**12a**) and thymidine (**12b**) were protected with acetyl groups by using acetic anhydride (see Scheme 6). The resultant acetates, **13a**,**b**, were converted to 4-thiouridine **14a** and 4-thiothymidine **14b**, respectively, by the Lawesson’s reagent. The deprotection of **14a**,**b** with K_2_CO_3_ in methanol afforded the free nucleosides **15a**,**b**, which were subsequently coupled with coumarin **2** to generate the desired conjugates **16a**,**b**.

Both 2-thiouracil (**17**) and 4-amino-2-mercaptopyrimidine (**19**) possess a thione functional group at the C2 position. The coupling of these two pyrimidine derivatives with coumarin **2** generated the ”tail-type” coumarin–2-thiopyrimidine conjugates **18** and **20** in 65% and 73% yields, respectively (see Scheme 7).

### 2.2. Structural Identification

The ^13^C NMR, ^1^H NMR, IR, and mass spectroscopic methods were applied to determine the structures of the 14 new conjugated compounds. The mass spectrum obtained from coumarin–thiopurine nucleoside **7b** using the ESIMS technique in positive ion mode showed a peak at 477.0628 for the [M + H]^+^ ion, indicating a molecular formula of C_20_H_18_ClN_4_O_6_S, which corresponds to a theoretical value of 477.0635. In the ^13^C NMR spectrum, the SCH_2_ carbon from the coupling reaction was observed at 27.22 ppm and the carbonyl carbon of the coumarin moiety was observed at 159.93 ppm. In the ^1^H NMR spectrum, a doublet at 5.98 ppm (J = 5.6 Hz) was observed which corresponded to the anomeric proton of the glycoside. Meanwhile, two distinctive singlets appeared at 4.51 ppm for the protons of the SCH_2_ and at 8.17 ppm for the proton of the CH=C–COO. One strong absorption band was observed in the IR spectrum at 1716 cm^−1^, which corresponds to the C=O stretching of the coumarin ring [38].

### 2.3. Evaluation of the Anti-HCV Activity

An evaluation of the antiviral effects of the conjugated compounds was initially performed in Huh 5-2 cells utilizing the HCV genotype 1b subgenomic replicon system [39], based on previously reported procedures [40]. By analyzing the dose–response curves, we calculated the concentration of each compound that inhibited the viral replication by 50% (EC50) and the concentration that reduced the host-cell metabolic activity by 50% (CC50). These values were then used to compute the selectivity index (SI = CC_50_/EC_50_), which serves as an indicator of a compound’s therapeutic window. Even if an EC50 was determined from the dose–response curve in the antiviral assay, the compounds that were evaluated may not qualify as promising hits. Only those selective inhibitors that achieve a significant decrease in virus replication (greater than 70%) and that do not negatively impact the metabolism of host cells are moved forward for further testing. It is likely that the antiviral activity observed in the other compounds is due to their nonspecific or pleiotropic effects on the host cell.

Among all of the newly synthesized conjugates, **7a** and **7b** showed impressive antiviral activity with a high window of selectivity (SI > 76 and >47, respectively) in the HCV 1b replicon assay system (see Table 1). Although compound **20** exhibited a higher SI value and a seemingly favorable EC_50_ at a dosage of 125 μg/mL, its inhibition performance was less robust, as it achieved only 69.8% inhibition at 50 μg/mL and less than 80% at 125 μg/mL. In contrast, compound **18** reached over 90% inhibition at 125 μg/mL, indicating a more consistent inhibitory effect. Furthermore, the antiviral activity of three of the hits (i.e., **7a**, **7b**, and **18**) was validated by evaluation of the dose–response effect of treatment with the compounds at the level of viral RNA replication in the Huh 9-13 assay system. The real-time quantitative RT-PCR readout was used. The results depicted in Table 2 indicate that the EC_50_ of all three compounds = 6.6–9.4 μM and that their SI > 16–41.

In previous studies by other research groups, several anti-HCV agents have demonstrated promising bioactivity. For example, the FDA-approved drug telaprevir (VX-950) exhibited an EC_50_ of 1.1 μM and a CC_50_ of ≥24 μM in Huh-5-2 cells [41], while boceprevir (SCH 503034) showed an EC_50_ of 0.93 μM with a CC_50_ of ≥7.4 μM [41]. Sofosbuvir, another widely used agent, has EC_50_ values ranging from 32 to 130 nM across different HCV genotypes [42]. Additionally, Han et al. reported that an N-protected indole scaffold (NINS) had an EC_50_ of 0.72 μM in Huh-7.5.1 cells [43]. More recently, Janssen BioPharma reported that AL-335, a 4′-fluoro-2′-C-substituted uridine 5′-phosphoramidate prodrug, exhibits an EC_50_ of 0.07 μM in the inhibition of the subgenomic HCV genotype 1b replicon [44]. Although the antiviral potency of our conjugated compounds does not reach the nanomolar level, their high CC_50_ values indicate low cytotoxicity, suggesting that these compounds may serve as valuable starting points for further optimization in anti-HCV drug development.

### 2.4. Early-Stage Safety Screening in Detroit 551 Assay (Normal Human Cells)

Detroit 551 cells, a human fibroblast cell line, are routinely employed to assess both acute and chronic toxicity in normal human cells. Evaluating the cytotoxicity of compounds in Detroit 551 cells helps to identify potential safety concerns early in drug development. The three promising leads, **7a**, **7b**, and **18**, were tested in a Detroit 551 assay using the antineoplastic and antibiotic agent actinomycin D as a positive control. As demonstrated in Table 3, none of the three compounds induced cell death at the highest tested concentration of 20 μM, suggesting that they are not highly toxic and are suitable for further development.

## 3. Discussion

### 3.1. Rationale of Anti-HCV Drug Design

Coumarin derivatives possess clinical potential [45,46], and were integrated in our previous work with thio-modified nucleobases and nucleosides to generate “one drug-like” leads with anti-HCV activity. Once the conjugated compounds are made to target viral enzymes, such as NS2·3 cysteine protease, NS3·4A serine protease, and NS5B RNA-dependent RNA polymerase, their enzymic activities and HCV replication could be inhibited [47]. The –SCH_2_– linker was used to incorporate the (6-chloro)coumarin moiety in adenine, guanine, purine, thymine, urine, and their corresponding nucleosides. Such an arrangement allowed us to place the coumarin moiety at the head (e.g., **7a**,**b**, **9a**,**b**, **11a**,**b**, and **16a**,**b**), the waist (e.g., **3a**,**b** and **5a**,**b**), and the tail positions (e.g., **18**, and **20**).

**Scheme 8:**
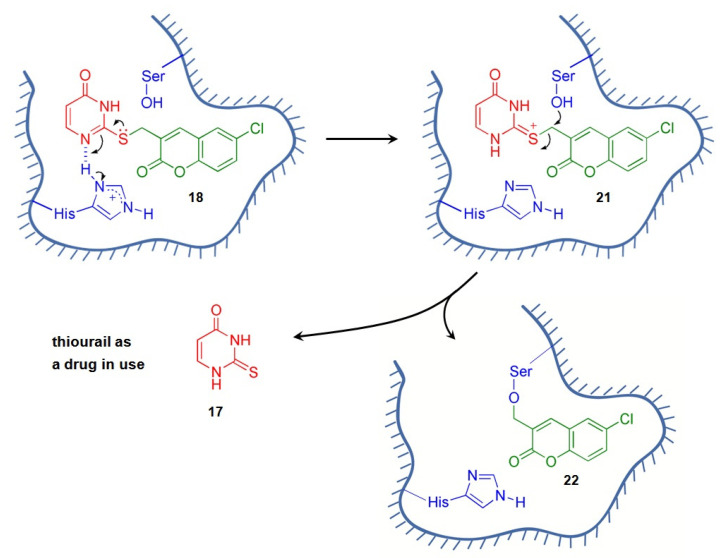
A plausible mechanistic pathway for the covalent inhibition of the viral protease by the coumarin–thiopyrimidine conjugate **18**.

Our design of the new leads involved a chemo-combination strategy, by which two biologically active compounds (or drugs) were amalgamated into one molecule. As an example, shown in Scheme 8, the uptake of the conjugated compound **18** into an HCV-infected cell leads to the formation of an H-bond between its N1 atom and the imidazole moiety of a histidine residue in the protease. The serine residue’s hydroxyl group is crucial in the catalytic mechanism, functioning as a nucleophile that donates electrons [48,49], which subsequently leads to a nucleophilic attack on the carbon atom of the electrophilic =S+CH_2_– group located in intermediate **21**. The formation of a covalent bond between the serine residue of the protease and the coumarin–thiopyrimidine conjugate **18** (i.e., the process of **21** → **22**) provides irreversible inhibition of the activity of the viral protease. As a consequence, thiouracil (**17**) is released by the cleavage of an S–C bond in **18**, from its tail position. Also known as 2-thioxo-1*H*-pyrimidin-4-one, thiouracil is the first thioamide anti-thyroid drug used as therapy for Graves’ disease [50,51]. Its trade names include Antagothyroil, Deracil, and Nobilen. The proposed mechanism of the covalent inhibition of the viral protease by the coumarin–thiopyrimidine conjugate **18** is further supported by analogous mechanisms observed with other drugs. For instance, the release of 6-mercaptopurine from azathioprine provides a useful comparison. In this process, the sulfur atom of deprotonated glutathione attacks the slightly electrophilic carbon center within the imidazole moiety of azathioprine, leading to the displacement and release of 6-mercaptopurine [52,53]. It is also possible that intracellular nucleophiles, such as glutathione, may contribute to this nucleophilic attack under certain conditions, further reinforcing the plausibility of our design rationale.

### 3.2. Structure–Activity Relationship

Analysis of the biological data in Table 1 and Table 2 enabled the derivation of SARs based on the EC_50_, CC_50_, and SI values of the conjugated compounds. These new compounds contained a common (6-chloro)coumarin moiety, which was attached to a purine or a pyrimidine nucleus through a –SCH_2_– linker at various positions:

(1) On the basis of the position of the coumarin moiety attached to the nucleobases and nucelosides of the conjugates, the three candidates selected for the Huh 9-13 assay system are “head-type” and “tail-type” conjugates. These targets, **7a**, **7b**, and **18**, showed impressive anti-HCV activity (6.6–9.4 μM) on the same order, and, among them, the “tail-type” **18** possessed the best SI value (i.e., >41);
(2) Regarding the purine nucleobases or the corresponding nucleosides, the SI values of the conjugates are better for the “head-type” than for the “waist-type” attachments (i.e., **7a**, **7b**, **9a**, and **9b** compared with **3a**, **3b**, **5a**, and **5b**, respectively). On the other hand, regarding the pyrimidine nucleobases or the corresponding nucleosides, the SI values of the conjugates are better for the “tail-type” than for the “head-type conjugates (i.e., **18** and **20** vs. **11a**, **11b**, **16a**, and **16b**);
(3) Switching the coumarin moiety from the wait position of a purine (i.e., **3a**,**b**) to the head position (i.e., **7a**,**b** and **9a**,**b**) resulted in the abatement of cytotoxicity;
(4) The removal of an amino group from the C2 position of coumarin–thioguanine **9a** gave 6-mercaptopurine conjugate **7a**, which exhibited a 7.6-fold improvement in its anti-HCV activity;
(5) Regarding its biological effects, the 2′,3′,5′-β-D-ribofuranosyl group plays an enigmatic role in terms of its incorporation in coumarin-purine conjugates. Its presence decreased the HCV inhibition and selectivity of the conjugates, which can be observed by comparing **3a** with **3b**; did not lead to a dramatic impact on the resulting activity and cytotoxicity, which can be observed by comparing **7a** with **7b**; and increased both the resulting HCV inhibition and selectivity, which can be observed by comparing **9a** with **9b**. Notwithstanding, it is certain that its presence improved the aqueous solubility of the conjugates.

## 4. Materials and Methods

### 4.1. General Information

Unless otherwise noted, the chemical reactions were performed in oven-dried (120 °C) glassware under a N_2(g)_ atmosphere. Solvents and reagents were sourced as follows: dichloromethane and MeOH from Mallinckrodt Chemicals Co. (Bridgewater, NJ, USA), CH_3_CN from Fischer Scientific (Waltham, MA, USA), and EtOAc along with hexanes—both dried and distilled from CaH_2_—from Echo Co. (Washington, DC, USA). We purchased NH_4_OH_(aq)_ from J. T. Baker Chemicals (Phillipsburg, NJ, USA). The following chemicals and reagents were supplied by Sigma-Aldrich (St. Louis, MA, USA): 6-mercaptopurine monohydrate (**6a**), 9-(β-D-ribofuranosyl)-6-mercaptopurine (**6b**), 6-thioguanine (**8a**), 6-thioguanosine (**8b**), 4-thiouracil (**10a**), 2-thiouracil (**17**), and 4-amino-2-mercaptopyrimidine (**19**). Lawesson’s reagent (2,4-bis(4-methoxyphenyl)-1,3,2,4-dithiadiphosphetane-2,4-dithione) was purchased from Alfa Aesar Chemicals Co. (Ward Hill, MA, USA), and potassium carbonate (K_2_CO_3_) from Showa Chemicals (Tokyo, Japan). Additionally, 8-mercaptoadenine [26] (**1a**), 8-mercaptoadenosine [54] (**1b**), 6-chloro-3-(chloromethyl)coumarins [36] (**2**), 8-mercaptoguanosine [55] (**4**), 5-methyl-4-thiouracil [37] (**10b**), 1-(2′,3′,5′-tri-O-acetyl-β-D-ribofuranosyl)uracil [56] (**13a**), and 1-(3′,5′-di-O-acetyl-β-D-ribofuranosyl)thymine [57] (**13b**) were prepared based on the known methods.

The silica gel 60 F 254 glass plates used for the thin layer chromatography were acquired from Merck (Boston, MA, USA). Purification was achieved by flash column chromatography using SiliCycle (Québec, QC, Canada) silica gel (40–63 microns, 230–400 mesh) ultrapure. Thermo (Waltham, MA, USA) 5 μm Hypersil ODS column (250 × 4.6 mm D.I.), two Waters HPLC 515 pumps (Waters Corporation, Milford, MA, USA), and a 2489 UV/Visible detector (Waters Corporation, Milford, MA, USA) were used in high-performance liquid chromatography (HPLC) analysis, which confirmed compound purities of >95.0%.

Infrared (IR) spectra were recorded using a PerkinElmer Spectrum One B spectrophotometer (PerkinElmer, Inc., Waltham, MA, USA) equipped with an attenuated total reflectance accessory. IR absorption bands are denoted as w (weak), m (medium), or s (strong). High-resolution mass spectra were obtained by means of the Thermo Scientific Q Exactive Plus hybrid quadrupole-orbitrap mass spectrometer (Waltham, MA, USA) at National Tsing Hua University (Hsinchu, Taiwan). ^1^H and ^13^C NMR experiments were performed on either a Bruker AC-400 (400 MHz) (Bruker Corporation, Billerica, MA, USA) or a Varian Mercury-400 (100 MHz) (Varian, Inc., Palo Alto, CA, USA) spectrometer, using CDCl_3_-*d* or DMSO-*d*_6_ as solvent. Proton chemical shifts were referenced to the solvent residual of CHCl_3_ (δ 7.24 ppm, singlet) or the center of the DMSO-*d*_6_ quintet (δ 2.49 ppm), while carbon-13 shifts were calibrated using residual CHCl_3_ (δ 77.0 ppm) or DMSO (δ 39.5 ppm). The various multiplicities are indicated as follows: s for a singlet, d for a doublet, t for a triplet, q for a quartet, and m for a multiplet, with J representing the coupling constant in hertz.

### 4.2. Standard Procedure for the Preparation of Conjugated Compounds **3**, **5**, **7**, **9**, **11**, **16**, **18**, and **20**

A nucleobase or nucleoside thione (**1**, **4**, **6**, **8**, **10**, **15, 17**, or **19**; 1.0 equiv) was dissolved in a mixture of H_2_O and CH_3_CN and treated with NH_4_OH_(aq)_, followed by stirring for 5–30 min. Subsequently, 6-chloro-3-(chloromethyl)coumarin (**2**, 1.2 equiv) was added, and the reaction mixture was stirred for an additional 40–120 min. After the reaction was finished, the mixture was evaporated under reduced pressure to obtain a crude product. The desired product was obtained by purifying the residue using silica gel column chromatography.

#### 4.2.1. 8-[(6′-Chlorocoumarin-3′-yl)methylthio]adenine (**3a**)

The established standard procedure was followed using 8-mercaptoadenine (**1a**, 42.3 mg, 0.253 mmol), H_2_O (5.0 mL), CH_3_CN (3.0 mL), and NH_4_OH_(aq)_ (0.25 mL). The solution was stirred at room temperature for 5.0 min, and **2** (69.5 mg, 0.303 mmol) was added. After another 2 h of stirring at room temperature, the solution was concentrated. The crude product was purified by flash column chromatography using 5.0% MeOH in dichloromethane as the eluant, affording **3a** as beige solids (55.7 mg, 0.155 mmol, 61% yield). ^1^H NMR results: (DMSO-*d*_6_, 400 MHz) δ 8.30 (s, 1 H, H-2), 8.01 (s, 1 H, CH=C–COO), 7.84 (d, *J* = 2.8 Hz, 1 H, ArH), 7.61 (dd, *J* = 8.8, 2.8 Hz, 1 H, ArH), 7.44 (d, *J* = 8.8 Hz, 1 H, ArH), 7.08 (s, 2 H, NH_2_), 4.30 (s, 2 H, SCH_2_); ^13^C NMR (DMSO-*d*_6_, 100 MHz) δ 159.74 (C=O), 153.90 (C-6), 152.09 (C-4), 151.55 (C-2 + C-9′), 146.17 (C-8), 140.47 (C-4′), 131.20 (C-7′), 128.37 (C-6′), 127.32 (C-5′), 125.34 (C-3′), 120.35 (C-10′), 119.24 (C-5), 118.10 (C-8′), 30.91 (SCH_2_); IR (ATR) 3394 (br, NH), 2926 (w, C–H), 1710 (s, C=O), 1602 (s, C=C), 1023 (s, C–O) cm^−1^; HRMS (ESI) *m*/*z* [M + H]^+^ calcd for C_15_H_11_ClN_5_O_2_S^+^: 360.0322, found 360.0315.

#### 4.2.2. 8-[(6′-Chlorocoumarin-3′-yl)methylthio]adenosine (**3b**)

The established standard procedure was followed using of 8-mercaptoadenosine (**1b**, 56.9 mg, 0.190 mmol), H_2_O (2.5 mL), CH_3_CN (1.5 mL), and NH_4_OH_(aq)_ (0.15 mL). The solution was stirred at room temperature for 30 min, and **2** (65.3 mg, 0.285 mmol) was added. After another 60 min of stirring at room temperature, the solution was concentrated. The crude product was purified by flash column chromatography (0–8.0% MeOH in EtOAc as the eluant) to give **3b** as pale yellow solids (68.4 mg, 0.139 mmol, 73% yield). ^1^H NMR results: (DMSO-*d*_6_, 400 MHz) δ 8.41 (s, 1 H, H-2), 8.04 (s, 1 H, CH=C–COO), 7.81 (d, *J* = 2.2 Hz, 1 H, ArH), 7.63 (dd, *J* = 8.8, 2.2 Hz, 1 H, ArH), 7.45–4.43 (m, 3 H, ArH + NH_2_), 5.67 (d, *J* = 6.8 Hz, 1 H, H-1″), 5.57 (dd, *J* = 8.2, 3.8 Hz, 1 H, OH), 5.40 (d, *J* = 6.4 Hz, 1 H, OH), 5.21 (d, *J* = 4.4 Hz, 1 H, OH), 4.96–4.91 (m, 1 H, H-2″), 4.35 (s, 2 H, SCH_2_), 4.12–4.09 (m, 1 H, H-3″), 3.92–3.90 (m, 1 H, H-4″), 3.63–3.60 (m, 1 H, H-5″), 3.50–3.45 (m, 1 H, H-5″); ^13^C NMR (DMSO-*d*_6_, 100 MHz) δ 159.77 (C=O), 154.60 (C-6), 151.65, 151.49, 150.54, 147.86 (C-8), 141.15 (C-4′), 131.32 (C-7′), 128.40 (C-6′), 127.30 (C-5′), 124.71 (C-3′), 120.39 (C-10′), 119.49 (C-5), 118.21 (C-8′), 88.75 (C-1″), 86.70 (C-4″), 71.33 (C-2″), 70.92 (C-3″), 62.15 (C-5″), 31.70 (SCH_2_); IR (ATR) 3360 (br, OH), 2891 (w, C–H), 1717 (m, C=O), 1659 (s, C=C), 994 (s, C–O) cm^−1^; HRMS (ESI) *m*/*z* [M + H]^+^ calcd for C_20_H_19_ClN_5_O_6_S^+^: 492.0744, found 492.0760.

#### 4.2.3. 8-[(6′-Chlorocoumarin-3′-yl)methylthio]guanosine (**5a**)

The standard procedure was followed by using 8-mercaptoguanosine (**4**, 62.4 mg, 0.198 mmol), H_2_O (5.0 mL), CH_3_CN (3.0 mL), and NH_4_OH_(aq)_ (0.25 mL). The solution was stirred at room temperature for 10 min, and **2** (54.4 mg, 0.238 mmol) was added. After another 60 min of stirring at room temperature, the solution was concentrated. The crude product was purified by flash column chromatography using 15% MeOH in CH_2_Cl_2_ as the eluant, affording **5a** as white solids (90.4 mg, 0.178 mmol, 90% yield). ^1^H NMR results: (DMSO-*d*_6_, 400 MHz) δ 10.73 (br, 1 H, NH), 7.90 (s, 1 H, CH=C–COO), 7.74 (d, *J* = 2.4 Hz, 1 H, ArH), 7.60 (dd, *J* = 8.8, 2.4 Hz, 1 H, ArH), 7.43 (d, *J* = 8.8 Hz, 1 H, ArH), 6.42 (s, 2 H, NH_2_), 5.70 (d, *J* = 6.4 Hz, 1 H, H-1″), 5.35 (d, *J* = 6.0 Hz, 1 H, OH), 5.08 (d, *J* = 4.8 Hz, 1 H, OH), 4.91 (dd, *J* = 6.0, 6.0 Hz, 1 H, OH), 4.89–4.84 (m, 1 H, H-2″), 4.23 (d, *J* = 14.0 Hz, 1 H, SCH), 4.19 (d, *J* = 14.0 Hz, 1 H, SCH), 4.10–4.08 (m, 1 H, H-3″), 3.80–3.77 (m, 1 H, H-4″), 3.62–3.54 (m, 1 H, H-5″), 3.49–3.43 (m, 1 H, H-5″); ^13^C NMR (DMSO-*d*_6_, 100 MHz) δ 159.64 (C=O), 155.63 (C-6), 153.13 (C-2), 152.47 (C-4), 151.51 (C-9′), 141.44 (C-8), 139.57 (C-4′), 131.20 (C-7′), 128.36 (C-6′), 127.28 (C-5′), 125.52 (C-3’), 120.25 (C-10’), 118.05 (C-8’), 117.42 (C-5), 88.27 (C-5″), 85.74(C-4″), 70.54 (C-2″+C-3″), 61.97 (C-5″), 32.80 (SCH_2_); IR (ATR) 3314 (bra, OH), 3070 (w, =C–H), 1711 (m, C=O), 1629 (s, C=C), 1584 (s, C=C), 1022 (s, C–O), 997 (s, C–O) cm^−1^; HRMS (ESI) *m*/*z* [M + H]^+^ calcd for C_20_H_19_ClN_5_O_7_S^+^: 508.0693, found 508.0694.

#### 4.2.4. 8-[(6’-Chlorocoumarin-3’-yl)methylthio]guanine (**5b**)

Hydrochloric acid (12 M, 0.40 mL) was added to a solution of **5a** (80.9 mg, 0.159 mmol) in MeOH (10 mL). The reaction mixture was then stirred at room temperature for 24 h to obtain precipitates, which were collected by vacuum filtration. The solids were washed by MeOH and then H_2_O to obtain **5b** as white solids (48.4 mg, 0.129 mmol, 81% yield). ^1^H NMR results: (DMSO-*d*_6_, 400 MHz) δ 11.22 (br, 1 H, NH), 7.93 (s, 1 H, CH=C–COO), 7.80 (d, *J* = 2.8 Hz, 1 H, ArH), 7.63 (dd, *J* = 8.8, 2.8 Hz, 1 H, ArH), 7.45 (d, *J* = 8.8 Hz, 1 H, ArH), 6.95 (br, 2 H, NH_2_), 4.21 (s, 2 H, SCH_2_); ^13^C NMR (DMSO-*d*_6_, 100 MHz) δ 159.72 (C=O), 156.01 (C-6), 155.58 (C-2), 153.79 (C-4), 151.42 (C-9′), 142.37 (C-8), 139.00 (C-4′), 130.72 (C-7′), 128.47 (C-6′), 127.09 (C-5′), 126.28 (C-3′), 123.00 (C-10′), 120.16 (C-8′), 117.59 (C-5), 31.80 (SCH_2_); IR (ATR) 3035 (w, C–C), 1698 (s, C=O), 1680 (s, C=C), 1330 (s, C–O) cm^−1^; HRMS (ESI) *m*/*z* [M + Na]^+^ calcd for C_15_H_10_ClN_5_NaO_3_S^+^: 398.0090, found 398.0073.

#### 4.2.5. 6-[(6′-Chlorocoumarin-3′-yl)methylthio]purine (**7a**)

The established standard procedure was followed using of 6-mercaptopurine (**6a**, 42.3 mg, 0.249 mmol), H_2_O (5.0 mL), CH_3_CN (5.0 mL), and NH_4_OH_(aq)_ (0.40 mL). The solution was stirred at room temperature for 20 min, and **2** (68.4 mg, 0.299 mmol) was added. After another 60 min of stirring at room temperature, the solution was concentrated. The crude product was purified by flash column chromatography using 3.0% MeOH in dichloromethane as the eluant, affording **7a** (65.5 mg, 0.190 mmol, 76% yield) as beige solids. ^1^H NMR results: (DMSO-*d*_6_, 400 MHz) δ 8.74 (s, 1 H, H-2), 8.45 (s, 1 H, H-8), 8.17 (s, 1 H, CH=C–COO), 7.87 (d, *J* = 2.5 Hz, 1 H, ArH), 7.60 (dd, *J* = 8.9, 2.5 Hz, 1 H, ArH), 7.44 (d, *J* = 8.9 Hz, 1 H, ArH), 4.50 (s, 2 H, SCH_2_); ^13^C NMR (DMSO-*d*_6_, 100 MHz) δ 159.91 (C=O), 157.33 (C-6), 151.45 (C-2), 151.35 (C-9′), 149.40 (C-4), 143.18 (C-8), 139.67 (C-4′), 131.13 (C-7′), 130.27 (C-5), 128.37 (C-6′), 127.44 (C-5′), 126.06 (C-3′), 120.27 (C-10′), 118.00 (C-8′), 27.17 (SCH_2_); IR (ATR) 3374 (m, NH), 1709 (s, C=O), 1636 (s, C=C), 1478 (m, C=C), 1272 (m, C–O), 1105 (m, C–O) cm^−1^; HRMS (ESI) *m*/*z* [M + H]^+^ calcd for C_15_H_10_ClN_4_O_2_S^+^: 345.0213, found 345.0205.

#### 4.2.6. 6-(6′-Chlorocoumarin-3′-yl)methylthio-9-(β-D-ribofuranos-1″-yl)purine (**7b**)

The established standard procedure was followed using 9-(β-D-ribofuranosyl)-6-mercaptopurine (**6b**, 61.2 mg, 0.215 mmol), H_2_O (5.0 mL), CH_3_CN (5.0 mL), and NH_4_OH_(aq)_ (0.25 mL). The solution was stirred at room temperature for 5.0 min, and **2** (59.2 mg, 0.258 mmol) was added. After another 60 min of stirring at room temperature, the solution was concentrated. The crude product was purified by flash column chromatography using 10% MeOH in dichloromethane as the eluant, affording **7b** as white solids (83.4 mg, 0.175 mmol, 81% yield). ^1^H NMR results: (DMSO-*d*_6_, 400 MHz) δ 8.79 (s, 1 H, H-2), 8.72 (s, 1 H, H-8), 8.17 (s, 1 H, CH=C–COO), 7.86 (d, *J* = 2.7 Hz, 1 H, ArH), 7.59 (dd, *J* = 8.9, 2.7 Hz, 1 H, ArH), 7.43 (d, *J* = 8.9 Hz, 1 H, ArH), 5.98 (d, *J* = 5.6 Hz, 1 H, H-1″), 5.51 (d, *J* = 6.0 Hz, 1 H, OH), 5.23 (d, *J* = 4.8 Hz, 1 H, OH), 5.10 (dd, *J* = 6.0, 5.2 Hz, 1 H, OH), 4.60–4.56 (m, 1 H, H-2″), 4.51 (s, 2 H, SCH_2_), 4.18–4.16 (m, 1 H, H-3″), 3.97–3.94 (m, 1 H, H-4″), 3.70–3.65 (m, 1 H, H-5″), 3.58–3.52 (m, 1 H, H-5″); ^13^C NMR (DMSO-*d*_6_, 100 MHz) δ 159.93 (C=O), 158.37 (C-6), 151.55 (C-2), 151.40 (C-9′), 148.35 (C-4), 143.51 (C-8), 139.79 (C-4′), 131.18 (C-7′), 128.41 (C-6′), 127.51(C-5′), 125.93 (C-3′), 120.30 (C-10′), 118.07 (C-8′), 87.88 (C-1″), 85.72 (C-4″), 73.78 (C-2″), 70.25 (C-3″), 61.21 (C-5″), 27.22 (SCH_2_); IR (ATR) 3394 (br, OH), 2923 (w, C–H), 1716 (s, C=O), 1560 (s, C=C), 1474 (m, C=C), 1212 (m, C–O), 1107 (s, C–O) cm^−1^; HRMS (ESI) *m*/*z* [M + H]^+^ calcd for C_20_H_18_ClN_4_O_6_S^+^: 477.0635, found 477.0628.

#### 4.2.7. 2-Amino-6-[(6′-chlorocoumarin-3′-yl)methylthio]purine (**9a**)

The standard procedure was followed using 6-thioguanine (**8a**, 31.7 mg, 0.190 mmol), H_2_O (3.0 mL), CH_3_CN (3.0 mL), and NH_4_OH_(aq)_ (0.40 mL). The solution was stirred at room temperature for 20 min, and **2** (52.1 mg, 0.227 mmol) was added. After another 2 h of stirring at room temperature, the solution was concentrated. The crude product was purified by flash column chromatography using 8.0% MeOH in dichloromethane as the eluant, affording **9a** as white solids (56.6 mg, 0.156 mmol, 83% yield). ^1^H NMR results: (DMSO-*d*_6_, 400 MHz) δ 12. 54 (br, 1 H, NH), 8.37 (s, 1 H, H-8), 7.88 (s, 1 H, CH=C–COO), 7.85 (d, *J* = 2.5 Hz, 1 H, ArH), 7.61 (dd, *J* = 8.7, 2.5 Hz, 1 H, ArH), 7.44 (d, *J* = 8.7 Hz, 1 H, ArH), 6.56 (s, 2 H, NH_2_), 4.29 (s, 2 H, SCH_2_); ^13^C NMR (DMSO-*d*_6_, 100 MHz) δ 159.89 (C=O), 159.54 (C-6), 157.44 (C-2), 151.81 (C-4), 151.42 (C-9′), 140.27 (C-4′), 138.96 (C-8), 131.03 (C-7′), 128.33 (C-6′), 127.19 (C-5′), 126.07 (C-3′), 123.69 (C-5), 120.44 (C-10′), 118.09 (C-8′), 27.14 (SCH_2_); IR (ATR) 3364 (m, NH), 3099 (w, C–H), 1699 (s, C=O), 1596 (s, C=C), 1490 (m, C=C), 1361 (m, CH), 1025 (s, C–O) cm^−1^; HRMS (ESI) *m*/*z* [M + H]^+^ calcd for C_15_H_11_ClN_5_O_2_S^+^: 360.0322, found 360.0323.

#### 4.2.8. 2-Amino-6-(6′-chlorocoumarin-3′-yl)methylthio-9-(β-D-ribofuranos-1″-yl)purine (**9b**)

The established standard procedure was followed using 6-thioguanosine (**8b**, 50.6 mg, 0.169 mmol), H_2_O (5.0 mL), CH_3_CN (5.0 mL), and NH_4_OH_(aq)_ (0.25 mL). The solution was stirred at room temperature for 10 min, and **2** (46.5 mg, 0.203 mmol) was added. After another 40 min of stirring at room temperature, the solution was concentrated. The crude product was purified by flash column chromatography using 10% MeOH in dichloromethane as the eluant, affording **9b** as pale yellow solids (58.5 mg, 0.119 mmol, 70% yield). ^1^H NMR results: (DMSO-*d*_6_, 400 MHz) δ 8.36 (s, 1 H, H-8), 8.17 (s, 1 H, CH=C–COO), 7.84 (d, *J* = 2.4 Hz, 1 H, ArH), 7.59 (dd, *J* = 8.8, 2.4 Hz, 1 H, ArH), 7.43 (d, *J* = 8.8 Hz, 1 H, ArH), 6.73 (s, 2 H, NH_2_), 5.76 (d, *J* = 6.0 Hz, 1 H, H-1″), 5.39 (d, *J* = 6.0 Hz, 1 H, OH), 5.13 (d, *J* = 4.8 Hz, 1 H, OH), 5.06 (dd, *J* = 5.6, 5.6 Hz, 1 H, OH), 4.47–4.42 (m, 1 H, H-2″), 4.31 (s, 2 H, SCH_2_), 4.10–4.07 (m, 1 H, H-3″), 3.89–3.87 (m, 1 H, H-4″), 3.64–3.60 (m, 1 H, H-5″), 3.54–3.49 (m, 1 H, H-5″); ^13^C NMR (DMSO-*d*_6_, 100 MHz) δ 159.87 (C=O), 159.40 (C-6), 158.39 (C-2), 151.44 (C-9′), 151.00 (C-4), 140.35 (C-4′), 139.10 (C-8), 131.08 (C-7′), 128.35 (C-6′), 127.19 (C-5′), 125.91 (C-3′), 124.09 (C-5), 120.43 (C-10′), 118.11 (C-8″), 86.62 (C-1″), 85.29 (C-4″), 73.46 (C-2″), 70.32 (C-3″), 61.31 (C-5″), 27.21 (SCH_2_); IR (ATR) 3309 (br, OH), 2922 (w, C–H), 1714 (s, C=O), 1567 (s, C=C), 1123 (m, C–O), 1023 (s, C–O), 997 (s, C–O) cm^−1^; HRMS (ESI) *m*/*z* [M + H]^+^ calcd for C_20_H_19_ClN_5_O_6_S^+^: 492.0744, found 492.0732.

#### 4.2.9. 4-[(6′-Chlorocoumarin-3′-yl)methylthio]uracil (**11a**)

The established standard procedure was followed by using 4-thiouracil (**10a**, 27.4 mg, 0.214 mmol), H_2_O (3.0 mL), CH_3_CN (3.0 mL), and NH_4_OH_(aq)_ (0.15 mL). The solution was stirred at room temperature for 5.0 min, and **2** (58.8 mg, 0.257 mmol) was added. After another 2 h of stirring at room temperature, the solution was concentrated. The crude product was purified by flash column chromatography using 5.0% MeOH in dichloromethane as the eluant, affording **11a** as white solids (49.7 mg, 0.155 mmol, 72% yield). ^1^H NMR results: (DMSO-*d*_6_, 400 MHz) δ 11.55 (br, 1 H, NH), 8.07 (s, 1 H, CH=C–COO), 7.85 (d, *J* = 2.4 Hz, 1 H, ArH), 7.64 (d, *J* = 6.4 Hz, 1 H, H-6), 7.59 (dd, *J* = 9.2, 2.4 Hz, 1 H, ArH), 7.45 (d, *J* = 9.2 Hz, 1 H, ArH), 6.31 (d, *J* = 6.4 Hz, 1 H, H-5), 4.23 (s, 2 H, SCH_2_); ^13^C NMR (DMSO-*d*_6_, 100 MHz) δ 175.64 (S-*C*=N, C-4), 159.86 (C=O), 154.15 (C-2), 151.40 (C-9′), 143.87 (C-6), 139.79 (C-4′), 131.24 (C-7′), 128.45 (C-6′), 127.40 (C-5′), 125.53 (C-3′), 120.24 (C-10′), 118.09 (C-8′), 101.89 (C-5), 27.92 (SCH_2_); IR (ATR) 3354 (br, OH), 2920 (w, C–H), 1716 (s, C=O), 1610 (s, C=C), 1416 (m, C–C), 1182 (m, C–N) cm^−1^; HRMS (ESI) *m*/*z* [M + H]^+^ calcd for C_14_H_10_ClN_2_O_3_S^+^: 321.0100, found 321.0093.

#### 4.2.10. 4-(6′-Chlorocoumarin-3′-yl)methylthio-5-methyluracil (**11b**)

The established standard procedure was followed using 5-methyl-4-thiouracil (**10b**, 27.1 mg, 0.191 mmol), H_2_O (5.0 mL), CH_3_CN (3.0 mL), and NH_4_OH_(aq)_ (0.25 mL). The solution was stirred at room temperature for 5.0 min, and **2** (52.5 mg, 0.229 mmol) was added. After another 2 h of stirring at room temperature, the solution was concentrated. The crude product was purified by flash column chromatography using 5.0% MeOH in dichloromethane as the eluent, affording **11b** as white solids (45.8 mg, 0.137 mmol, 72% yield). ^1^H NMR results: (DMSO-*d*_6_, 400 MHz) δ 8.09 (s, 1 H, CH=C–COO), 7.84 (d, *J* = 2.5 Hz, 1 H, ArH), 7.63 (dd, *J* = 9.1, 2.5 Hz, 1 H, ArH), 7.54 (s, 1 H, H-6), 7.46 (d, *J* = 9.1 Hz, 1 H, ArH), 4.25 (s, 2 H, SCH_2_), 1.92 (s, 3 H, CH_3_); ^13^C NMR (DMSO-*d*_6_, 100 MHz) δ 175.75 (C-1), 159.87 (C=O), 153.93 (C-2), 151.38 (C-9′), 141.46 (C-6), 139.86 (C-4′), 131.21 (C-7′), 128.42 (C-6′), 127.36 (C-5′), 125.44 (C-3′), 120.23 (C-10′), 118.07 (C-8′), 109.63 (C-5), 27.99 (SCH_2_), 12.93 (CH_3_); IR (ATR) 2796 (w, C–H), 1705 (s, C=O), 1610 (m, C=C), 1538 (m, C=C), 1278 (m, C–C), 1182 (m, C–O) cm^−1^; HRMS (ESI) *m*/*z* [M + H]^+^ calcd for C_15_H_12_ClN_2_O_3_S^+^: 335.0257, found 335.0259.

#### 4.2.11. 1-(2′,3′,5′-Tri-O-acetyl-β-D-ribofuranosyl)-4-thiouracil (**14a**)

A solution containing 1-(2′,3′,5′-tri-*O*-acetyl-β-D-ribofuranosyl)uracil (**13a**, 517.4 mg, 1.397 mmol) and Lawesson’s reagent (847.6 mg, 2.096 mmol) in 1,2-dichloroethane (25 mL) was stirred at reflux for 4.0 h. After the reaction was quenched with saturated NaHCO_3(aq)_ solution (25 mL), the resultant solution was extracted with dichloromethane (3 × 25 mL). The combined organic layers were washed with brine (30 mL), dried over anhydrous MgSO_4(s)_, filtered, and concentrated under reduced pressure to yield a residue, which was purified by flash column chromatography (50% hexanes in EtOAc as the eluant) to afford **14a** as yellow solids (498.7 mg, 1.291 mmol, 92% yield). ^1^H NMR results: (CDCl_3_, 400 MHz) δ 7.21 (d, *J* = 7.6 Hz, 1 H, H-6), 6.41 (d, *J* = 7.6 Hz, 1 H, H-5), 5.96 (d, *J* = 4.8 Hz, 1 H, H-1″), 5.33–5.20 (m, 2 H, H-2″ + H-3″), 4.36–4.33 (m, 3 H, H-4″ + 2 × H-5″), 2.12 (s, 3 H, CH_3_), 2.11 (s, 3 H, CH_3_), 2.09 (s, 3 H, CH_3_); ^13^C NMR (CDCl_3_, 100 MHz) δ 189.51 (C=S), 170.05 (C=O), 169.57, (C=O), 147.19 (C-2), 133.50 (C-6), 113.84 (C-5), 87.90 (C-1′), 80.12 (C-4′), 72.83 (C-2′), 70.05 (C-3′), 62.94 (C-5′), 20.76 (CH_3_), 20.47 (CH_3_), 20.40 (CH_3_). Its spectroscopic characteristics align with those previously documented for the same substance [58].

#### 4.2.12. 1-(3′,5′-Di-O-acetyl-β-D-ribofuranosyl)-4-thiothymine (**14b**)

A solution containing 1-(3′,5′-di-*O*-acetyl-β-D-ribofuranosyl)thymine (**13b**, 613 mg, 1.88 mmol) and Lawesson’s reagent (912 mg, 2.25 mmol) in 1,2-dichloroethane (30 mL) was stirred at reflux for 4.0 h. After the reaction was quenched with saturated NaHCO_3(aq)_ solution (25 mL), the resultant solution was extracted with dichloromethane (3 × 25 mL). The combined organic layers were washed with brine (30 mL), dried over anhydrous MgSO_4(s)_, filtered, and concentrated under reduced pressure to yield a residue, which was purified by flash column chromatography (40% hexanes in EtOAc as the eluant) to afford **14b** as yellow solids (581 mg, 1.67 mmol, 90% yield). ^1^H NMR results: (CDCl_3_, 400 MHz) δ 10.10 (br, 1 H, NH), 7.32 (s, 1 H, H-6), 6.24 (dd, *J* = 8.4, 5.6 Hz, 1 H, H-1″), 5.21–5.19 (m, 1 H, H-3″), 4.39–4.25 (m, 3 H, H-4″ + 2 × H-5″), 2.55–2.49 (m, 1 H, H-2″), 2.19–2.12 (m, 1 H, H-2″), 2.09 (s, 6 H, 2 × CH_3_), 2.08 (s, 3 H, CH_3_); ^13^C NMR (CDCl_3_ 100 MHz) δ 190.39 (C=S), 170.40 (C=O), 170.17, (C=O), 147.85 (C-2), 130.65 (C-6), 119.80 (C-5), 85.41 (C-1′), 82.46 (C-4′), 73.98 (C-3), 63.70 (C-5′), 37.78 (C-2′), 20.82 (CH_3_), 20.76 (CH_3_), 17.26 (CH_3_); IR (neat) 3218 (w, NH), 3073 (w, C–H), 2925 (w, C–H), 1747 (s, C=O), 1713 (s, C=O), 1634 (s, C=C), 1464 (m, C–C), 1239 (s, C–O) cm^−1^; MS (ESI^+^) *m*/*z* 343 (MH^+^); HRMS (ESI) calcd for (C_14_H_18_N_2_O_6_S + H)^+^: 343.0964, found 343.0958.

#### 4.2.13. 4-Thiouridine (**15a**)

K_2_CO_3_ (187.6 mg, 1.357 mmol) was added to a solution of **14a** (524.4 mg, 1.357 mmol) in MeOH (10 mL). After stirring the reaction mixture at room temperature overnight, MeOH was evaporated under reduced pressure to yield a residue, which was purified by flash column chromatography using 10% MeOH in dichloromethane as the eluent, affording **15a** as yellow solids (263.8 mg, 1.014 mmol, 75% yield). ^1^H NMR results: (DMSO-*d*_6_, 400 MHz) δ 7.82 (d, *J* = 7.4 Hz, 1 H, H-6), 6.30 (d, *J* = 7.4 Hz, 1 H, H-5), 5.72 (d, *J* = 4.4 Hz, 1 H, H-1″), 5.44 (d, *J* = 4.8 Hz, 1 H, OH), 5.11–5.07 (m, 2 H, OH), 4.03–4.01 (m, 1 H, H-2″), 3.96–3.94 (m, 1 H, H-3″), 3.86–3.84 (m, 1 H, H-4″), 3.65–3.62 (m, 1 H, H-5″), 3.56–3.53 (m, 1 H, H-5″). Its ^1^H NMR data match those previously reported for this compound [59].

#### 4.2.14. 4-Thiothymidine (**15b**)

K_2_CO_3_ (69.7 mg, 0.504 mmol) was added to a solution of **14b** (173 mg, 0.504 mmol) in MeOH (20 mL). After stirring the reaction mixture at room temperature overnight, MeOH was evaporated under reduced pressure to yield a residue, which was subsequently purified by flash column chromatography using 10% MeOH in EtOAc as the eluent to afford **15b** as yellow solids (95.8 mg, 0.371 mmol, 74% yield). ^1^H NMR results: (DMSO-*d*_6_, 400 MHz) δ 12.67 (br, 1 H, NH), 7.88 (s, 1 H, H-6), 6.10 (dd, *J* = 6.4, 6.8 Hz, 1 H, H-1″), 5.24 (d, *J* = 4.4 Hz, 1 H, OH), 5.06 (dd, *J* = 5.2, 5.2 Hz, 1 H, OH), 4.26–4.21 (m, 1 H, H-3″), 3.80–3.77 (m, 1 H, H-4″), 3.64–3.59 (m, 1 H, H-5″), 3.58–3.52 (m, 1 H, H-5″), 2.15–2.12 (m, 2 H, 2 × H-2″), 1.96 (s, 3 H, CH_3_). Its spectroscopic characteristics align with those previously documented for the same substance [60].

#### 4.2.15. 4-[(6′-Chlorocoumarin-3′-yl)methylthio]uridine (**16a**)

The established standard procedure was followed using **15a** (50.4 mg, 0.194 mmol), H_2_O (5.0 mL), CH_3_CN (3.0 mL), and NH_4_OH_(aq)_ (0.25 mL). The solution was stirred at room temperature for 10 min, and **2** (53.2 mg, 0.232 mmol) was then added. After another 50 min of stirring at room temperature, the solution was concentrated. The crude product was purified by flash column chromatography using 8.0% MeOH in dichloromethane as the eluant, affording **16a** as white solids (69.1 mg, 0.153 mmol, 78% yield). ^1^H NMR results: (DMSO-*d*_6_, 400 MHz) δ 8.27 (d, *J* = 7.2 Hz, 1 H, H-6), 8.09 (s, 1 H, CH=C–COO), 7.84 (d, *J* = 2.6 Hz, 1 H, ArH), 7.62 (dd, *J* = 8.9, 2.6 Hz, 1 H, ArH), 7.45 (d, *J* = 8.9 Hz, 1 H, ArH), 6.49 (d, *J* = 7.2 Hz, 1 H, H-5), 5.73 (d, *J* = 2.4 Hz, 1 H, H-1″), 5.48 (d, *J* = 4.8 Hz, 1 H, OH), 5.16 (dd, *J* = 5.2, 4.8 Hz, 1 H, OH), 5.04 (d, *J* = 4.8 Hz, 1 H, OH), 4.25 (s, 2 H, SCH_2_), 3.96–3.93 (m, 2 H, H-2″ + H-3″), 3.90–3.88 (m, 1 H, H-4″), 3.74–3.70 (m, 1 H, H-5″), 3.59–3.56 (m, 1 H, H-5″); ^13^C NMR (DMSO-*d*_6_, 100 MHz) δ 175.02 (C-4), 159.82 (C=O), 152.79 (C-2), 151.41 (C-9′), 141.91 (C-6), 139.87 (C-4′), 131.25 (C-7′), 128.43 (C-6′), 127.40 (C-5′), 125.38 (C-3′), 120.22 (C-10′), 118.08 (C-8′), 102.53 (C-5), 90.18 (C-1″), 84.22 (C-4″), 74.49 (C-2″), 68.61 (C-3″), 59.78 (C-5″), 28.19 (SCH_2_); IR (ATR) 3474 (br, OH), 1693 (s, C=O), 1603 (s, C=C), 1260 (s, C–O), 1036 (s, C–O) cm^−1^; HRMS (ESI) *m*/*z* [M + H]^+^ calcd for C_19_H_18_ClN_2_O_7_S^+^: 453.0523, found 453.0515.

#### 4.2.16. 4-[(6′-Chlorocoumarin-3′-yl)methylthio]thymidine (**16b**)

The established standard procedure was followed using **15b** (50.1 mg, 0.194 mmol), H_2_O (5.0 mL), CH_3_CN (3.0 mL), and NH_4_OH_(aq)_ (0.25 mL). The solution was stirred at room temperature for 10 min, and **2** (53.3 mg, 0.233 mmol) was then added. After another 50 min of stirring at room temperature, the solution was concentrated. The crude product was purified by flash column chromatography using 8.0% MeOH in dichloromethane as the eluant, affording **16b** as white solids (70.6 mg, 0.157 mmol, 81% yield). ^1^H NMR results: (DMSO-*d*_6_, 400 MHz) δ 8.07 (s, 1 H, CH=C–COO), 8.00 (s, 1 H, H-6), 7.81 (d, *J* = 2.4 Hz, 1 H, ArH), 7.60 (dd, *J* = 8.8, 2.4 Hz, 1 H, ArH), 7.43 (d, *J* = 8.8 Hz, 1 H, ArH), 6.05 (dd, *J* = 6.4, 6.4 Hz, 1 H, H-1″), 5.21 (d, *J* = 4.4 Hz, 1 H, OH), 5.06 (dd, *J* = 5.2, 4.8 Hz, 1 H, OH), 4.24 (s, 2 H, SCH_2_), 4.21–4.17 (m, 2 H, H-3″), 3.81–3.79 (m, 1 H, H-4″), 3.63–3.58 (m, 1 H, H-5″), 3.56–3.52 (m, 1 H, H-5″), 2.23–2.19 (m, 1 H, H-2″), 2.02–1.96 (m, 1 H, H-2″), 1.94 (s, 3 H, CH_3_); ^13^C NMR (DMSO-*d*_6_, 100 MHz) δ 175.05 (C-4), 159.84 (C=O), 152.31 (C-2), 151.39 (C-9′), 139.94 (C-4′), 139.21 (C-6), 131.23 (C-7′), 128.41 (C-6′), 127.37 (C-5′), 125.30 (C-3′), 120.21 (C-10′), 118.07 (C-8′), 110.28 (C-5), 87.78 (C-1″), 85.89 (C-4″), 69.76 (C-3″), 60.79 (C-5″), 40.70 (C-2″), 28.28 (SCH_2_), 13.45 (CH_3_); IR (ATR) 3244 (br, OH), 1728 (m, C=O), 1706 (m, C=O), 1639 (s, C=C), 1032 (s, C–O) cm^−1^; HRMS (ESI) *m*/*z* [M + Na]^+^ calcd for C_20_H_19_ClN_2_NaO_6_S^+^: 473.0550, found 473.0543.

#### 4.2.17. 2-[(6′-Chlorocoumarin-3′-yl)methylthio]uracil (**18**)

The established standard procedure was followed using 2-thiouracil (**17**, 27.6 mg, 0.215 mmol), H_2_O (3.0 mL), CH_3_CN (3.0 mL), and NH_4_OH_(aq)_ (0.15 mL). The solution was stirred at room temperature for 5.0 min, and **2** (59.2 mg, 0.258 mmol) was then added. After another 2 h of stirring at room temperature, the solution was concentrated. The crude product was purified by flash column chromatography (5.0–20% MeOH in dichloromethane as the eluant) to give **18** as white solids (44.6 mg, 0.139 mmol, 65% yield). ^1^H NMR results: (Pyridine-*d*_5_ + DMSO-*d*_6_, 400 MHz) δ 8.04 (s, 1 H, CH=C–COO), 7.98 (d, *J* = 6.6 Hz, 1 H, H-6), 7.59 (d, *J* = 2.6 Hz, 1 H, ArH), 7.41 (dd, *J* = 8.8, 2.6 Hz, 1 H, ArH), 7.21 (d, *J* = 8.8 Hz, 1 H, ArH), 6.30 (d, *J* = 6.6 Hz, 1 H, H-5), 4.41 (s, 2 H, SCH_2_); ^13^C NMR (Pyridine-*d*_5_ + DMSO-*d*_6_, 100 MHz) δ 164.87 (C-2), 163.93 (C-4), 160.44 (C=O), 154.78 (C-6), 151.99 (C-9′), 139.87 (C-4′), 131.25 (C-7′), 129.15 (C-6′), 127.69 (C-5′), 126.45 (C-3′), 120.75 (C-10′), 118.08 (C-8′), 109.76 (C-5), 29.96 (SCH_2_); IR (ATR) 2926 (w, C–H), 1704 (s, C=O), 1538 (m, C=C), 1277 (m, C–O), 1181 (m, C–O) cm^−1^; HRMS (ESI) *m*/*z* [M + H]^+^ calcd for C_14_H_10_ClN_2_O_3_S^+^: 321.0100, found 321.0101.

#### 4.2.18. 4-Amino-2-[(6′-chlorocoumarin-3′-yl)methylthio]pyrimidine (**20**)

The established standard procedure was followed using 4-amino-2-mercaptopyrimidine (**19**, 27.4 mg, 0.215 mmol), H_2_O (3.0 mL), CH_3_CN (3.0 mL), and NH_4_OH_(aq)_ (0.50 mL). The solution was stirred at room temperature for 30 min, and **2** (49.3 mg, 0.258 mmol) was added. After another 2 h of stirring at room temperature, the mixture was concentrated. The crude product was purified by flash column chromatography using 20% hexanes in EtOAc as the eluent, affording **20** as white solids (50.2 mg, 0.157 mmol, 73% yield). ^1^H NMR results: (DMSO-*d*_6_, 400 MHz) δ 8.19 (s, 1 H, CH=C–COO), 7.89 (d, *J* = 5.8 Hz, H, H-6), 7.87 (d, *J* = 2.7 Hz, 1 H, ArH), 7.60 (dd, *J* = 8.9, 2.7 Hz, 1 H, ArH), 7.43 (d, *J* = 8.9 Hz, 1 H, ArH), 7.01 (s, 2 H, NH_2_), 6.14 (d, *J* = 5.8 Hz, 1 H, H-5), 4.09 (s, 2 H, SCH_2_); ^13^C NMR (DMSO-*d*_6_, 100 MHz) δ 168.60 (C-2), 163.15 (C-4), 159.93 (C=O), 155.04 (C-6), 151.35 (C-9′), 139.30 (C-4′), 130.96 (C-7′), 128.34 (C-6′), 127.25 (C-5′), 126.46 (C-3′), 120.43 (C-10′), 118.04 (C-8′), 101.35 (C-5), 29.36 (SCH_2_); IR (neat) 3422 (m, NH), 2920 (w, C–H), 1714 (s, C=O), 1648 (m, C=C), 1586 (m, C=C), 1252 (m, C–O) cm^−1^; HRMS (ESI) *m*/*z* [M + H]^+^ calcd for C_14_H_11_ClN_3_O_2_S^+^: 320.0260, found 320.0265.

### 4.3. Antiviral Assay and Cell Viability Assay

#### 4.3.1. Anti-HCV Assay in Huh 5-2 Cells

Huh 5-2 cells were plated in white, tissue culture-treated 96-well view plates (Packard, Canberra, Canada) at a density of 5 × 10^3^ cells per well, using complete Dulbecco’s Modified Eagle Medium (DMEM) containing 250 µg/mL G418. Following 24 h incubation at 37 °C with 5% CO_2_, the medium was discarded and serial 3-fold dilutions of the test compounds—prepared in complete DMEM lacking G418—were added to reach a final volume of 100 µL per well. After an additional 4-day incubation period under the same conditions, the medium was removed, and luciferase activity was assessed using the Steady-Glo luciferase assay kit (Promega, Leiden, The Netherlands). Luminescence was measured with a Luminoskan Ascent reader (Thermo, Vantaa, Finland). The EC_50_ value was determined as the compound concentration resulting in a 50% decrease in luciferase signal.

#### 4.3.2. Anti-HCV Assay in Huh 9-13 Cells

Huh 9-13 cells were seeded at 5 × 10^3^ cells per well into 96-well plates containing complete DMEM supplemented with 1000 µg/mL G418. After 24 h of incubation at 37 °C, the medium was replaced with 3-fold serial dilutions of test compounds prepared in complete DMEM without G418 or hygromycin, which were added to a final volume of 100 µL per well. After 4 days of incubation, the culture medium was removed, and the cell monolayers were washed once with phosphate-buffered saline (PBS). Cells were then lysed using 350 µL of RLT buffer (Qiagen, Venlo, The Netherlands) as per the manufacturer’s protocol, and the lysates were stored at −80 °C for future analysis. Upon completion of sample collection, RNA was extracted, and the samples were analyzed through quantitative real-time PCR to measure their HCV replicon content. The real-time RT-PCR data for all samples were calibrated against the “no-drug control”.

#### 4.3.3. Cell Viability Assay in Detroit 551 Cells

Detroit 551 cells were harvested and seeded into a 96-well plate, then incubated at 37 °C for 24 h. A serial dilution of the test compound was prepared in eight three-fold concentrations (20,000 nM, 6667 nM, 2222 nM, 741 nM, 247 nM, 82 nM, 27 nM, and 9 nM), and 10 µL of each dilution was added to the corresponding wells. The plate was then returned to 37 °C for an additional 72 h of incubation. After this incubation, a 3-(4,5-dimethylthiazol-2-yl)-5-(3-carboxymethoxyphenyl)-2-(4-sulfophenyl)-2*H*-tetrazolium (MTS) assay medium (mixed in a 2:0.1 ratio of MTS–phenazine methyl sulfate (PMS)) was prepared. The plate was moved to a biological safety cabinet, and 21 µL of the MTS medium was added to each well. The plate was then incubated at 37 °C for 90 min before absorbance was measured at 490 nm on a Victor 2 plate reader (PerkinElmer, Inc., Waltham, MA, USA). Data were subsequently analyzed to assess cytotoxicity.

## 5. Conclusions

A 6-Chlorocoumarin moiety was incorporated into nucleobases and nucleosides through a –SCH_2_– linker to produce head-, waist-, and tail-type conjugated compounds. In this new compound library, three of the conjugates exhibited appealing inhibitory effects on the HCV subgenomic replicon replication in Huh 5-2 cells. Moreover, the “head-type” coumarin–6-mercaptopurinosines **7a** and **7b** showed EC_50_ values of 7.3 and 6.6 μM, respectively, and SI values >16–20 in Huh 9-13 cells; the “tail-type” coumarin–2-thiopyrimidine **18** showed an EC_50_ value of 9.4 μM and an SI value > 41. Their success was attributed to the application of the chemo-combination strategy to their molecular design, which allowed a possible liberation of the drugs 6-mercaptopurine ribonucleoside from **7a** and **7b** and of thiouracil from **18**. The structure–activity relationships among these conjugated compounds were established, which will assist our further work on the mechanisms of drug action to be performed in due course.

## Figures and Tables

**Table 1:** Inhibitory activity of the 14 compounds against HCV genotype **1b** replicons in Huh 5-2 cells.

Compound	Connection	50 μg/mL ^a^			125 μg/mL ^b^		
	Type	CC_50_ ^c^ (μM)	EC_50_ ^d^ (μM)	SI ^e^	CC_50_ ^c^ (μM)	EC_50_ ^d^ (μM)	SI ^e^
**3a**	waist	69	10	6.7	—	—	—
**3b**	waist	48	32	1.5	—	—	—
**5a**	waist	>98	>98	ND ^f^	—	—	—
**5b**	waist	>133	51	>2.6	—	—	—
**7a**	head	>145	8.6	>17	>363	4.8	>76
**7b**	head	>105	8.8	>12	>262	5.6	>47
**9a**	head	>139	65	>2.1	>347	48	>7.3
**9b**	head	>102	15	>6.9	—	—	—
**11a**	head	>156	18	>8.6	—	—	—
**11b**	head	>149	16	>9.1	—	—	—
**16a**	head	>108	16	>6.8	—	—	—
**16b**	head	>111	59	>1.9	—	—	—
**18**	tail	>156	48	>3.2	>390	87	>4.5
**20**	tail	>156	53	>2.9	>391	21	>19

^a^ Interferon α-2b served as a positive reference at a dosage of 10,000 units per well, effectively reducing the signal in the viral RNA (luciferase) assay to background levels without causing any cytotoxic effects. In the primary screening, compounds were initially tested at 50 µg/mL (with values converted to µM). ^b^ Compounds meeting the hit selection criteria (i.e., greater than 70% inhibition of the luminescence signal) were required to show this effect at concentrations that did not negatively impact host-cell metabolism. Only these hits were advanced to a secondary screen, which began at 125 µg/mL. The EC_50_ depicted in the table is the average of triplicate determinations. ^c^ The CC_50_ is defined as the concentration at which a 50% reduction in host-cell metabolic activity is observed, as determined by the MTS assay. ^d^ The EC_50_ represents the concentration at which a 50% inhibition of virus replication is detected, based on luminescence measurements. ^e^ The selectivity index is calculated as the ratio of CC_50_–EC_50_. ^f^ ND: not determined.

**Table 2:** Inhibitory activity of compounds **7a**, **7b**, and **18** on HCV subgenomic replicon replication in Huh 9-13 cells.

Compound	Connection Type	CC_50_ ^a^ (μM)	EC_50_ ^b^ (μM)	SI ^c^
**7a**	head	>145	7.3	>20
**7b**	head	>105	6.6	>16
**18**	tail	>390 ^d^	9.4	>41

^a^ The CC_50_ is defined as the concentration at which a 50% reduction in host-cell metabolic activity is observed. ^b^ The EC_50_ represents the concentration at which a 50% inhibition of virus replication is detected. ^c^ The selectivity index is calculated as the ratio of CC_50_–EC_50_. ^d^ Compound was tested starting at 125 µg/mL.

**Table 3:** Cell cytotoxicity study of conjugated compounds **7a**, **7b**, and **18** in Detroit 551 cells ^a,b^.

Compound	Connection Type	IC_50_ ^c^ (μM)
**7a**	head	>20.0
**7b**	head	>20.0
**18**	tail	>20.0
Actinomycin D	—	15.4

^a^ IC_50_ values were determined from the cell survival curve after compound incubation for 72 h. ^b^ Compound was tested starting at 20.0 μM. ^c^ IC_50_ is defined as the inhibitory concentration corresponding to a cell survival rate of 50%.

## Data Availability

The data is available within the article and Supplementary Materials.

## Supplementary Materials

The following supporting information can be downloaded at: https://www.mdpi.com/article/10.3390/molecules30081776/s1.

## References

[1] Stroffolini T., Stroffolini G. (2024). Prevalence and modes of transmission of hepatitis C virus infection: A historical worldwide review. Viruses.

[2] World Health Organization (WHO). https://www.who.int/news-room/fact-sheets/detail/hepatitis-c.

[3] Meshram R., Kolte B., Gacche R. (2024). Reverse vaccinology approach for identification of epitopes from E1 protein as peptide vaccine against HCV: A proof of concept. Vaccine.

[4] Bhattacharjee C., Singh M., Das D., Chaudhuri S., Mukhopadhyay A. (2021). Current therapeutics against HCV. Virusdisease.

[5] Spengler U. (2018). Direct antiviral agents (DAAs)—A new age in the treatment of hepatitis C virus infection. Pharmacol. Ther..

[6] Ren X.-D., Fu X., He Y.-Q., Li C.-Y., Guo M., Qiao M. (2022). Safety and efficacy of sofosbuvir-velpatasvir: A meta-analysis. Medicine.

[7] Mengshetti S., Zhou L., Sari O., De Schutter C., Zhang H., Cho J.H., Tao S., Bassit L.C., Verma K., Domaoal R.A. (2019). Discovery of a Series of 2′-α-Fluoro,2′-β-bromo-ribonucleosides and their phosphoramidate prodrugs as potent pan-genotypic inhibitors of hepatitis C virus. J. Med. Chem..

[8] Nio Y., Sasai M., Akahori Y., Okamura H., Hasegawa H., Oshima M., Watashi K., Wakita T., Ryo A., Tanaka Y. (2019). Bardoxolone methyl as a novel potent antiviral agent against hepatitis B and C viruses in human hepatocyte cell culture systems. Antiviral Res..

[9] Zhang H., Zheng X., Li J., Liu Q., Huang X.-X., Ding H., Suzuki R., Muramatsu M., Song S.-J. (2021). Flavonoid-triazolyl hybrids as potential anti-hepatitis C virus agents: Synthesis and biological evaluation. Eur. J. Med. Chem..

[10] Pawlotsky J.-M. (2012). New antiviral agents for hepatitis C. F1000 Biol. Rep..

[11] Yeung K.-S., Yeung K.-S., Meanwell N.A., Qiu Z., Hernandez D., Zhang S., McPhee F., Weinheimer S., Clark J.M., Janc J.W. (2001). Structure–activity relationship studies of a bisbenzimidazole-based, Zn^2+^-dependent inhibitor of HCV NS3 serine protease. Bioorg. Med. Chem. Lett..

[12] Chen C.-S., Chiou C.-T., Chen G.S., Chen S.-C., Hu C.-Y., Chi W.-K., Chu Y.-D., Hwang L.-H., Chen P.-J., Chen D.-S. (2009). Structure-based discovery of triphenylmethane derivatives as inhibitors of hepatitis C virus helicase. J. Med. Chem..

[13] François J., Raymond A. (2008). Small-Molecule Hepatitis C Virus (HCV) NS3/4A Serine Protease Inhibitors. WO Patent.

[14] De Clercq E. (2007). The Design of Drugs for HIV and HCV. Nat. Rev. Drug Discov..

[15] Njoroge F.G., Chen K.X., Shih N.-Y., Piwinski J.J. (2008). Challenges in modern drug discovery: A case study of Boceprevir, an HCV protease inhibitor for the treatment of hepatitis C virus infection. Accounts Chem. Res..

[16] Lin C., Kwong A.D., Perni R.B. (2006). Discovery and development of VX-950, a novel, covalent, and reversible inhibitor of hepatitis C virus NS3·D4A serine protease. Infect. Disord. Drug Targets.

[17] Paul B., Chen M.F., Paterson A.R.P. (1975). Inhibitors of nucleoside transport. a structure–activity study using human erythrocytes. J. Med. Chem..

[18] Yadav V., Chu C.K., Rais R.H., Al Safarjalani O.N., Guarcello V., Naguib F.N.M., el Kouni M.H. (2004). Synthesis, biological activity and molecular modeling of 6-benzylthioinosine analogues as subversive substrates of *Toxoplasma gondii* adenosine kinase. J. Med. Chem..

[19] Fu Y.H., Xu Z.X., Jiang N., Zheng Y.P., Rameix-Welti M.A., Jiao Y.Y., Peng X.L., Wang Y., Eleouet J.F., Cen S. (2019). High-throughput screening of active compounds against human respiratory syncytial virus. Virology.

[20] Cheng K.W., Cheng S.C., Chen W.Y., Lin M.H., Chuang S.J., Cheng I.H., Sun C.Y., Chou C.Y. (2015). Thiopurine analogs and mycophenolic acid synergistically inhibit the papain-like protease of Middle East respiratory syndrome coronavirus. Antiviral Res..

[21] de Carvalho O.V., Felix D.M., de Mendonca L.R., de Araujo C., de Oliveira Franca R.F., Cordeiro M.T., Silva Junior A., Pena L.J. (2017). The thiopurine nucleoside analogue 6-methylmercaptopurine riboside (6MMPr) effectively blocks Zika virus replication. Int. J. Antimicrob. Agents.

[22] Hoover S., Striker R. (2008). Thiopurines inhibit bovine viral diarrhea virus production in a thiopurine methyltransferase-dependent manner. J. Gen. Virol..

[23] Elion G.B. (1989). The purine path to chemotherapy. Science.

[24] Podsiadlo P., Sinani V.A., Bahng J.H., Kam N.W.S., Lee J., Kotov N.A. (2008). Gold nanoparticles enhance the anti-leukemia action of a 6-mercaptopurine chemotherapeutic agent. Langmuir.

[25] Miron T., Arditti F., Konstantinovski L., Rabinkov A., Mirelman D., Berrebi A., Wilchek M. (2009). Novel derivatives of 6-mercaptopurine: Synthesis, characterization and antiproliferative activities of *s*-allylthio-mercaptopurines. Eur. J. Med. Chem..

[26] Borrmann T., Abdelrahman A., Volpini R., Lambertucci C., Alksnis E., Gorzalka S., Knospe M., Schiedel A.C., Cristalli G., Müller C.E. (2009). Structure–activity relationships of adenine and deazaadenine derivatives as ligands for adenine receptors, a new purinergic receptor family. J. Med. Chem..

[27] Hocek M., Holý A., Dvořáková H. (2002). Cytostatic 6-arylpurine nucleosides IV. synthesis of 2-substituted 6-phenylpurine ribonucleosides. Collect. Czech. Chem. Commun..

[28] Neyts J. (2006). Selective inhibitors of hepatitis C virus replication. Antiviral Res..

[29] De Francesco F., Carfí A. (2007). Advances in the development of new therapeutic agents targeting the NS3-4A serine protease or the NS5B RNA-dependent RNA polymerase of the hepatitis C virus. Adv. Drug Deliv. Rev..

[30] Lange C.M., Sarrazin C., Zeuzem S. (2010). Review article: Specifically targeted anti-viral therapy for hepatitis C–a new era in therapy. Aliment. Pharmacol. Ther..

[31] Jazwinski A.B., Muir A.J. (2011). Direct-acting antiviral medications for chronic hepatitis C virus infection. Gastroenterol. Hepatol..

[32] Tsay S.-C., Lin S.-Y., Huang W.-C., Hsu M.-H., Hwang K.C., Lin C.-C., Horng J.-C., Chen I.-C., Hwu J.R., Shieh F.-K. (2016). Synthesis and structure-activity relationships of imidazole-coumarin conjugates against hepatitis C virus. Molecules.

[33] Hwu J.R., Singha R., Hong S.C., Chang Y.H., Das A.R., Vliegen I., De Clercq E., Neyts J. (2008). Synthesis of New Benzimidazole–Coumarin Conjugates as Anti-Hepatitis C Virus Agents. Antiviral Res..

[34] Neyts J., De Clercq E., Singha R., Chang Y.H., Das A.R., Chakraborty S.K., Hong S.C., Tsay S.-C., Hsu M.-H., Hwu J.R. (2009). Structure–activity relationship of new anti-hepatitis C virus agents: Heterobicycle–coumarin conjugates. J. Med. Chem..

[35] Hwu J.R., Lin S.-Y., Tsay S.-C., De Clercq E., Leyssen P., Neyts J. (2011). Coumarin–purine ribofuranoside conjugates as new agents against hepatitis C virus. J. Med. Chem..

[36] Kaye P.T., Musa M.A. (2002). A convenient and improved Bayli–Hillman synthesis of 3-substututed 2*H*-1-benzopyran-2-ones. Synthesis.

[37] Kaneko K., Katayama H., Wakabayashi T., Kumonaka T. (1988). Pyrimidine derivatives; 1. the highly regioselective 4-thionation of pyrimidine-2,4(1*H*,3*H*)-dione derivatives with Lawesson reagent. Synthesis.

[38] Silverstein R.M., Webster F.X., Kiemle D.J. (2005). Spectrometric Identification of Organic Compounds.

[39] Lohmann V., Körner F., Koch J.-O., Herian U., Theilmann L., Bartenschlager R. (1999). Replication of subgenomic hepatitis C virus RNAs in a hepatoma cell line. Science.

[40] Paeshuyse J., Kaul A., De Clercq E., Rosenwirth B., Dumont J.-M., Scalfaro P., Bartenschlager R., Neyts J. (2006). The non-immunosuppressive cyclosporin DEBIO-025 is a potent inhibitor of hepatitis C virus replication in vitro. Hepatology.

[41] Vliegen I., Paeshuyse J., De Burghgraeve T., Lehman L.S., Paulson M., Shih I.-H., Mabery E., Boddeker N., De Clercq E., Reiser H. (2009). Substituted imidazopyridines as potent inhibitors of HCV replication. J. Hepatol..

[42] Han B., Martin R., Xu S., Parvangada A., Svarovskaia E.S., Mo H., Dvory-Sobol H. (2019). Sofosbuvir susceptibility of genotype 1 to 6 HCV from DAA-naïve subjects. Antiviral Res..

[43] Han Z., Liang X., Wang Y., Qing J., Cao L., Shang L., Yin Z. (2016). The discovery of indole derivatives as novel hepatitis C virus inhibitors. Eur. J. Med. Chem..

[44] Wang G., Dyatkina N., Prhavc M., Williams C., Serebryany V., Hu Y., Huang Y., Wan J., Wu X., Deval J. (2019). Synthesis and anti-HCV activities of 4’-fluoro-2’-substituted uridine triphosphates and nucleotide prodrugs: Discovery of 4’-fluoro-2’-C-methyluridine 5’-phosphoramidate prodrug (AL-335) for the treatment of hepatitis C infection. J. Med. Chem..

[45] Ito C., Itoigawa M., Mishina Y., Filho V.C., Enjo F., Tokuda H., Nishino H., Furukawa H. (2003). Chemical constituents of *Calophyllum brasiliense*. 2. structure of three new coumarins and cancer chemopreventive activity of 4-substituted coumarins. J. Nat. Prod..

[46] Nam N.-H., Kim Y., You Y.-J., Hong D.-H., Kim H.-M., Ahn B.-Z. (2002). Preliminary structure–antiangiogenic activity relationships of 4-senecioyloxymethyl-6,7- dimethoxycoumarin. Bioorg. Med. Chem. Lett..

[47] Lin C., Tan S.-L. (2006). Hepatitis C Viruses: Genomes and Molecular Biology.

[48] Wei Y., Huang M., Jiang L. (2024). Advancements in serine protease inhibitors: From mechanistic insights to clinical applications. Catalysts.

[49] Rajagopalan S., Wang C., Yu K., Kuzin A.P., Richter F., Lew S., Miklos A.E., Matthews M.L., Seetharaman J., Su M. (2014). Design of activated serine–containing catalytic triads with atomic-level accuracy. Nat. Chem. Biol..

[50] Gerabek W.E., Haage B.D., Keil G., Wegner W. (2005). Enzyklopädie Medizingeschichte.

[51] Bunevicius R., Prange A.J. (2006). Psychiatric manifestations of graves’ hyperthyroidism: Pathophysiology and treatment options. CNS Drugs.

[52] Kurtovic S., Modén O., Shokeer A., Mannervik B. (2008). Structural determinants of glutathione transferases with azathioprine activity identified by DNA shuffling of alpha class members. J. Mol. Biol..

[53] Lazarević S., Đanic M., Al-Salami H., Mooranian A., Mikov M. (2022). Gut Microbiota Metabolism of Azathioprine: A New Hallmark for Personalized Drug-Targeted Therapy of Chronic Inflammatory Bowel Disease. Front. Pharmacol..

[54] Holmes R.E., Robins R.K. (1964). Purine nucleosides. VII. direct bromination of adenosine, deoxyadenosine, guanosine, and related purine nucleosides. J. Am. Chem. Soc..

[55] Lin T.-S., Cheng J.-C., Ishiguro K., Sartorelli A.C. (1985). 8-Substituted guanosine and 2′-deoxyguanosine derivatives as potential inducers of the differentiation of friend erythroleukemia cells. J. Med. Chem..

[56] Matsuda A., Itoh H., Takenuki K., Sasaki T., Ueda T. (1988). Alkyl addition reaction of pyrimidine 2′-ketonucleosides: Synthesis of 2′-branched-chain sugar pyrimidine nucleosides (nucleosides and nucleotides. LXXXI). Chem. Pharm. Bull..

[57] Lusic H., Young D.D., Lively M.O., Deiters A. (2007). Photochemical DNA activation. Org. Lett..

[58] Kaleta Z., Makowski B.T., Soós T., Dembinski R. (2006). Thionation using fluorous Lawesson’s Reagent. Org. Lett..

[59] National Center for Biotechnology Information PubChem Compound Summary for CID 3032615, Thiouridine. https://pubchem.ncbi.nlm.nih.gov/compound/Thiouridine.

[60] Palomino E., Meltsner B.R., Kessel D., Horwitz J.P. (1990). Synthesis and in Vitro evaluation of some modified 4-thiopyrimidine nucleosides for prevention or reversal of AIDS-associated neurological disorders. J. Med. Chem..

