# Supplementary material for: 6-Chlorocoumarin Conjugates with Nucleobases and Nucleosides as Potent Anti-Hepatitis C Virus Agents
Source: Molecules. 2025 Apr 15;30(8):1776. doi: 10.3390/molecules30081776 (PMC12029179; doi:10.3390/molecules30081776)
Supplement: Supplementary file 1 [file molecules-30-01776-s001.zip › molecules-3518700-supplementary.pdf]

Supplementary Material

6-Chlorocoumarin Conjugates with Nucleobases and Nucleosides as Potent Anti-Hepatitis C Virus Agents

Shu-Yu Lin [1,*], Wen-Chieh Huang [1], Shwu-Chen Tsay [2,*], Johan Neyts [3], Pieter Leyssen [3], Chun-Cheng Lin [2], Kuo Chu Hwang [2], Jia-Cherng Horng [2] and Jih Ru Hwu [2,*]

[1] Institute of Biotechnology and Pharmaceutical Research, National Health Research Institutes, Miaoli County, 350401, Taiwan.
[2] Department of Chemistry and Frontier Research Center on Fundamental & Applied Sciences of Matters, National Tsing Hua University, Hsinchu 300044, Taiwan.
[3] Rega Institute for Medical Research, Katholieke Universiteit Leuven, Minderbroedersstraat 10, B-3000 Leuven, Belgium.

Corresponding Author:

Shu-Yu Lin, email: shuyulin@nhri.edu.tw; Shwu-Chen Tsay, email: tsay.susan@gmail.com; Jih Ru Hwu, email: jrhwu@mx.nthu.edu.tw.

Table of Contents S1

1. ^{1}H NMR, ^{13}C NMR, HRMS, and IR spectra of **3**−**20** S2

2. Dose-Response Curve of **7a**, **7b** and **18** in Huh 5-2 Cells S29

3. Dose-Response Data of **7a**, **7b** and **18** in Huh 9-13 Cells S32

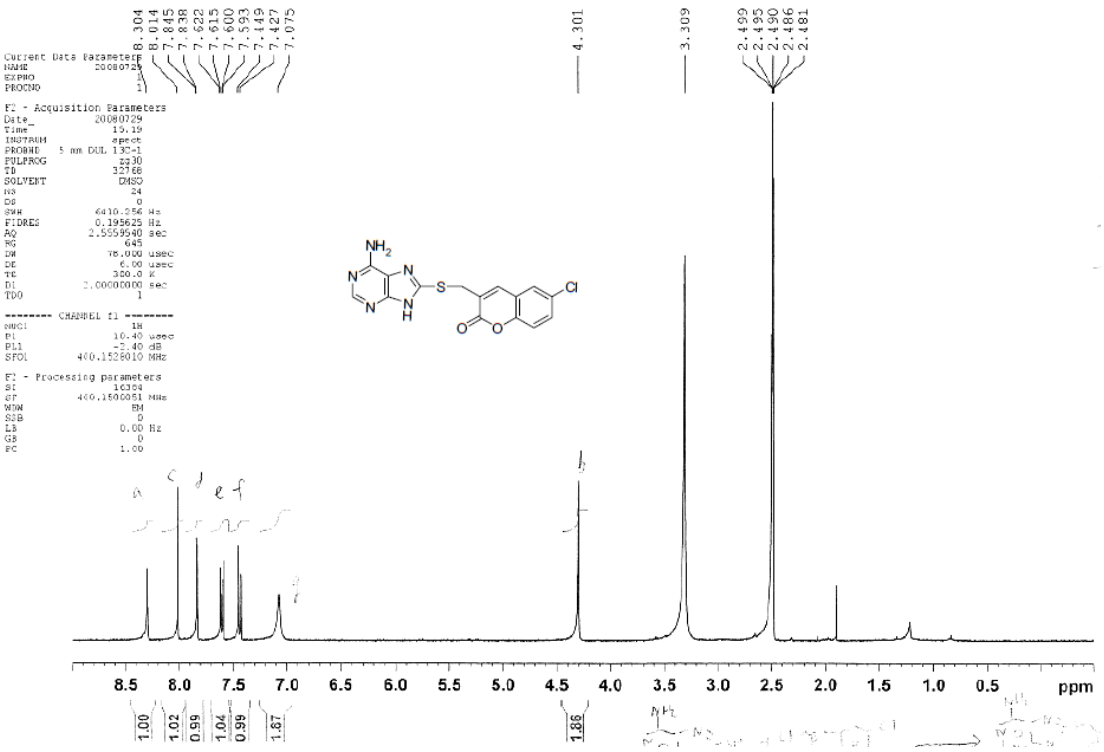

^{1}H NMR of Compound **3a**

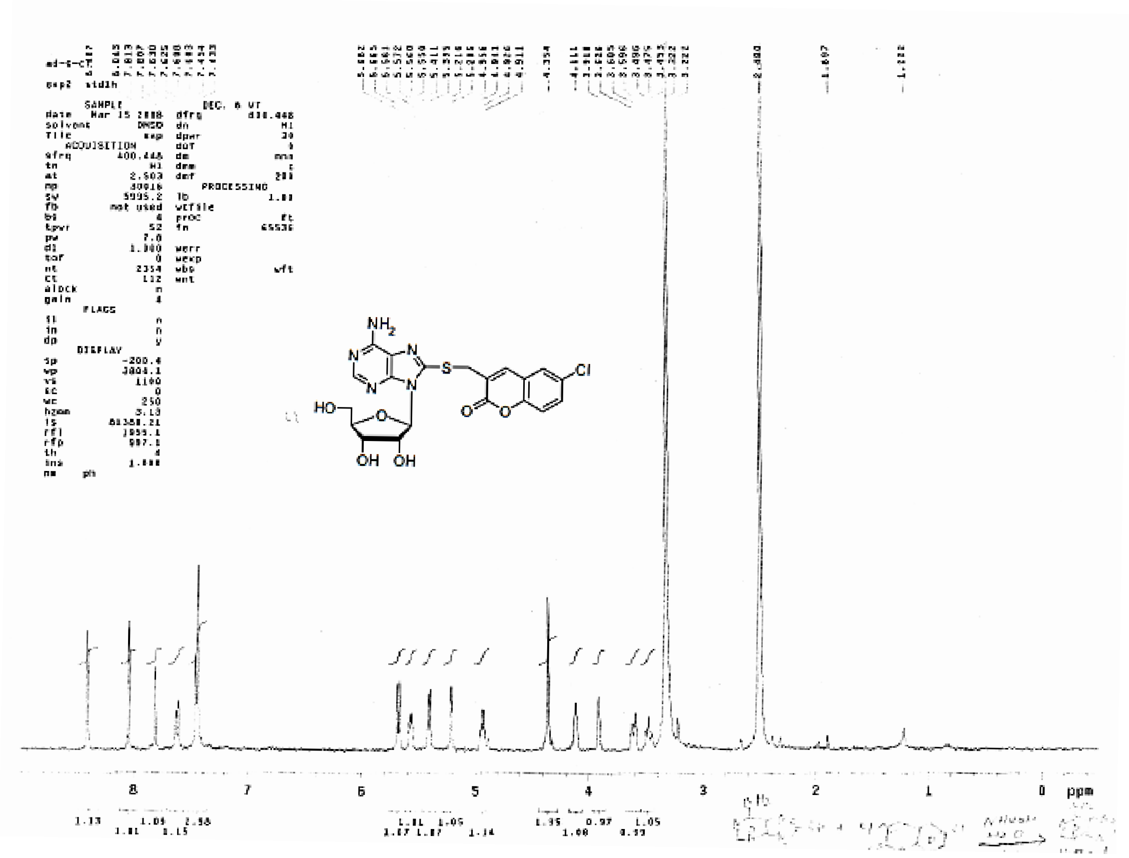

^{1}H NMR of Compound **3b**

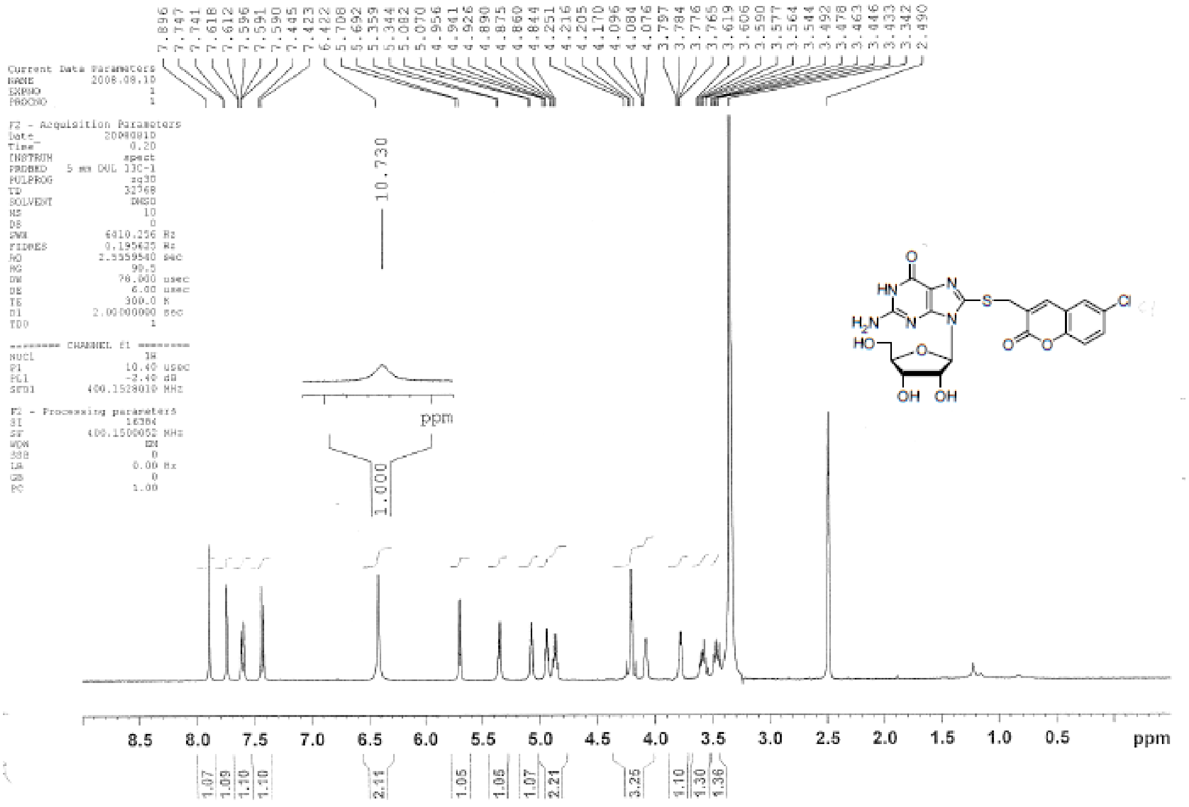

^{1}H NMR of Compound **5a**

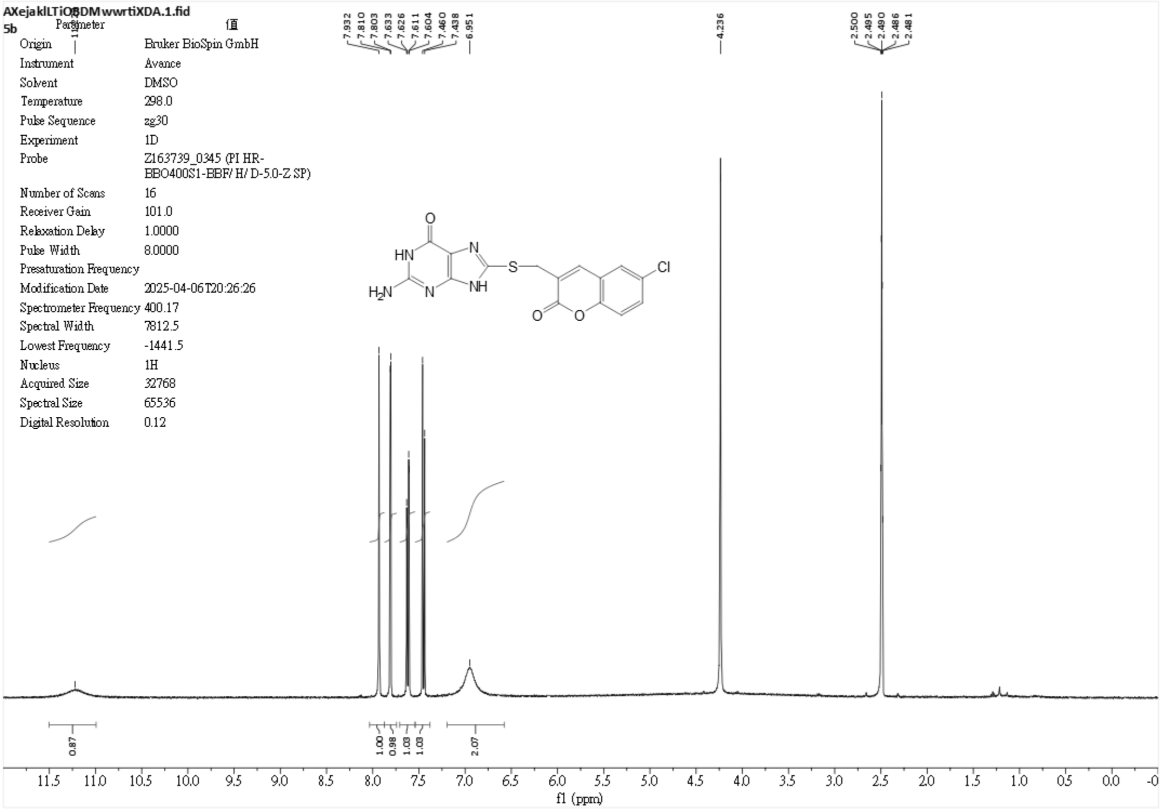

^{1}H NMR of Compound **5b**

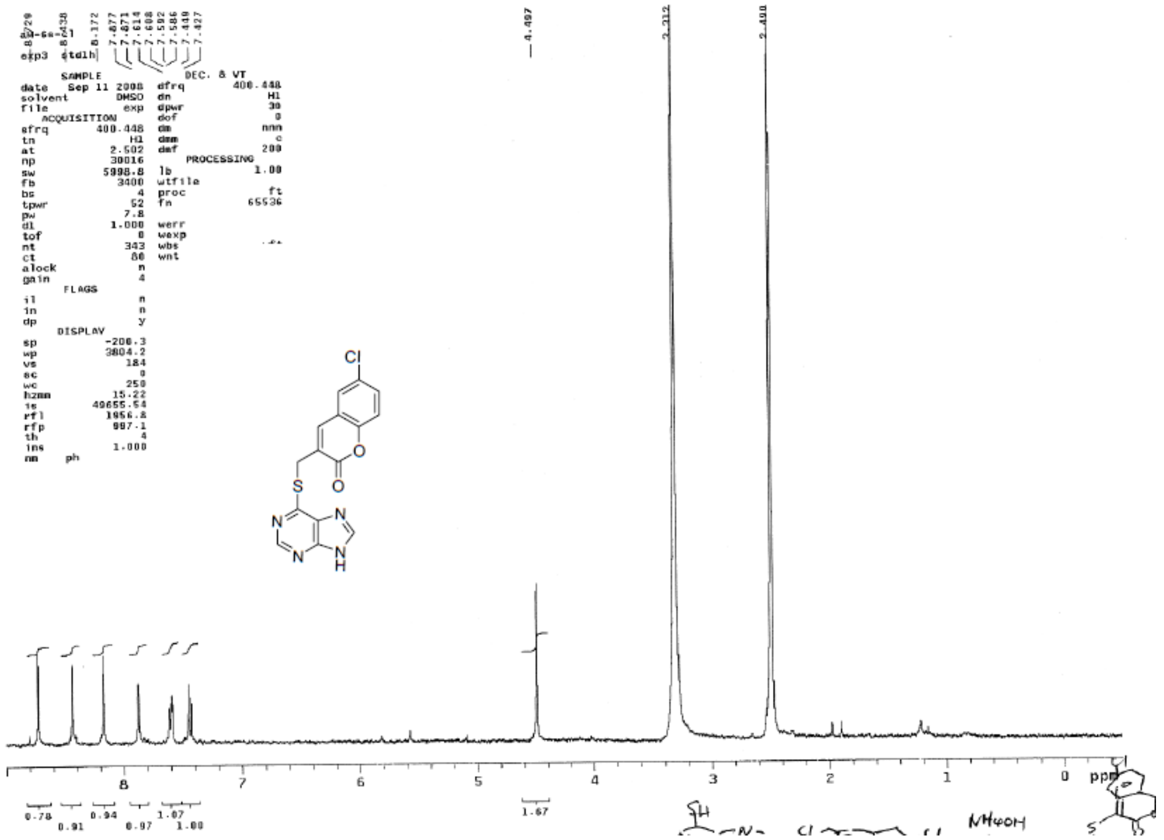

^{1}H NMR of Compound **7a**

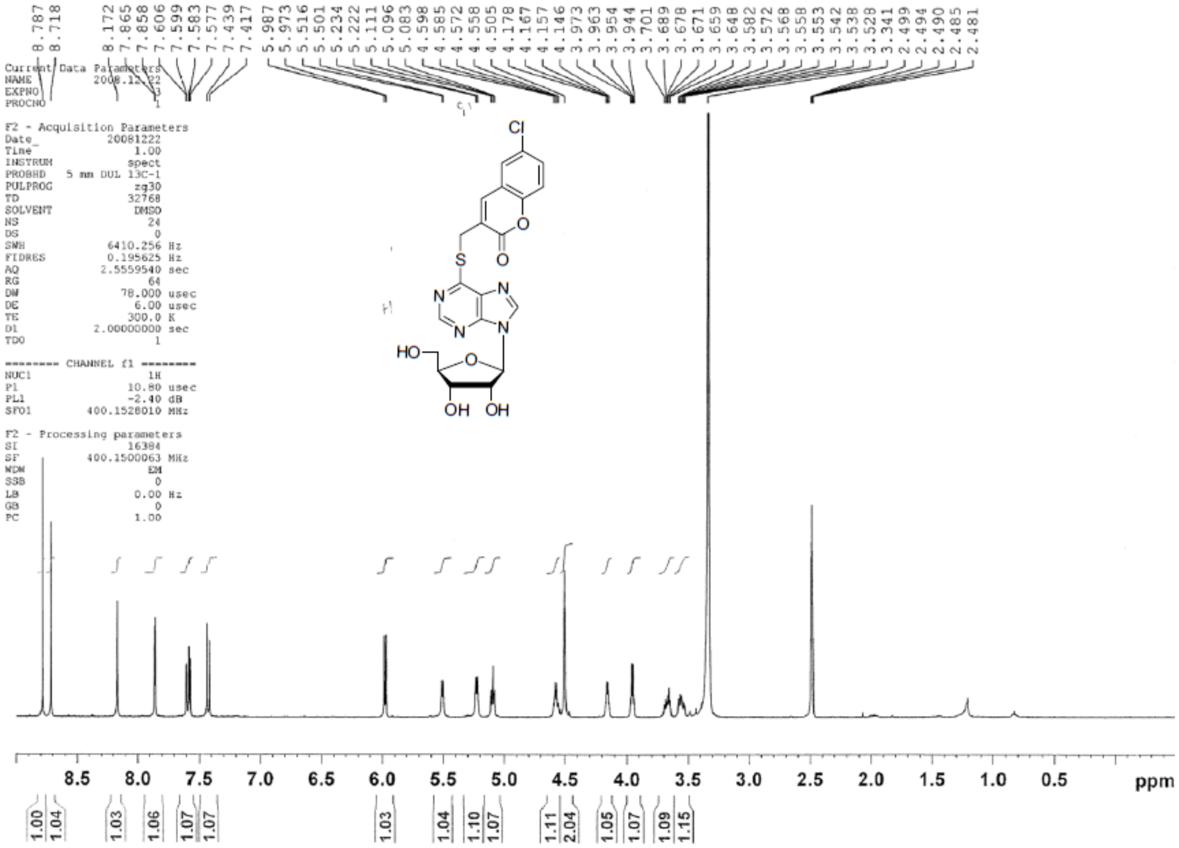

^{1}H NMR of Compound **7b**

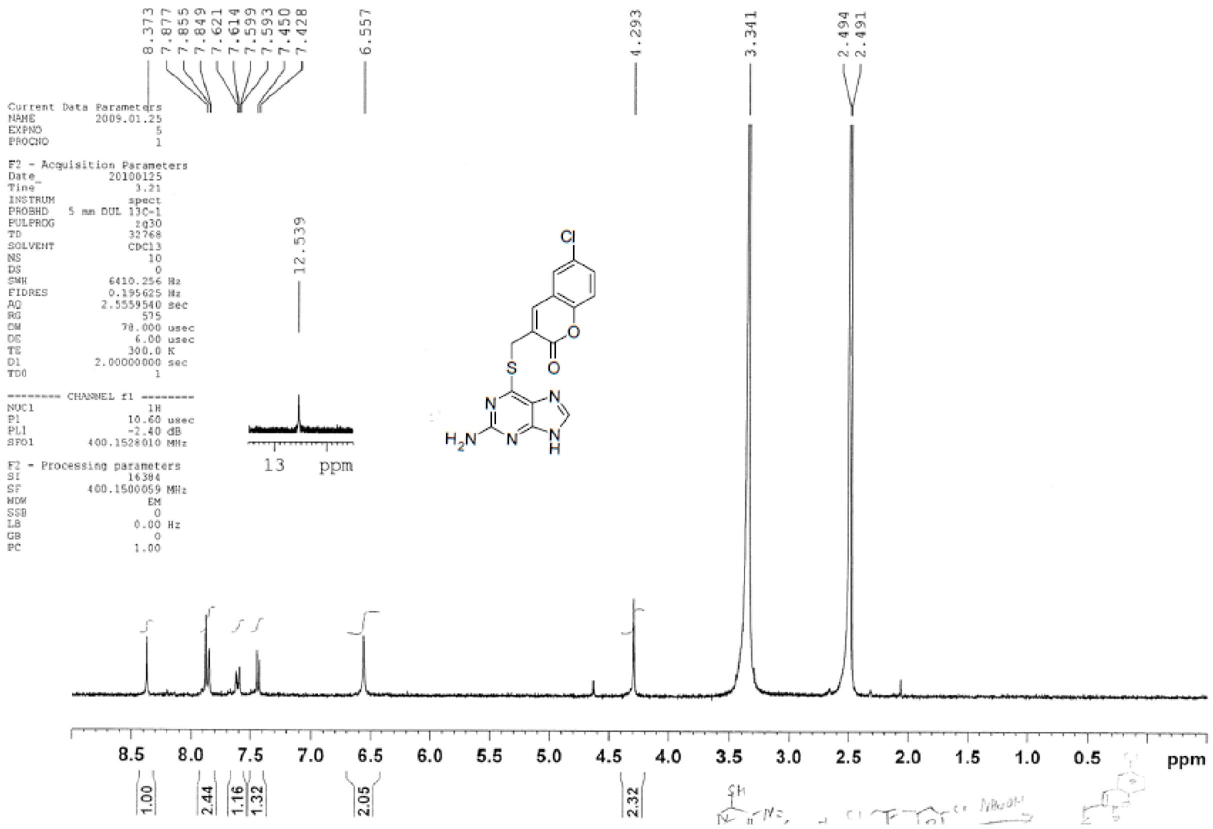

^{1}H NMR of Compound **9a**

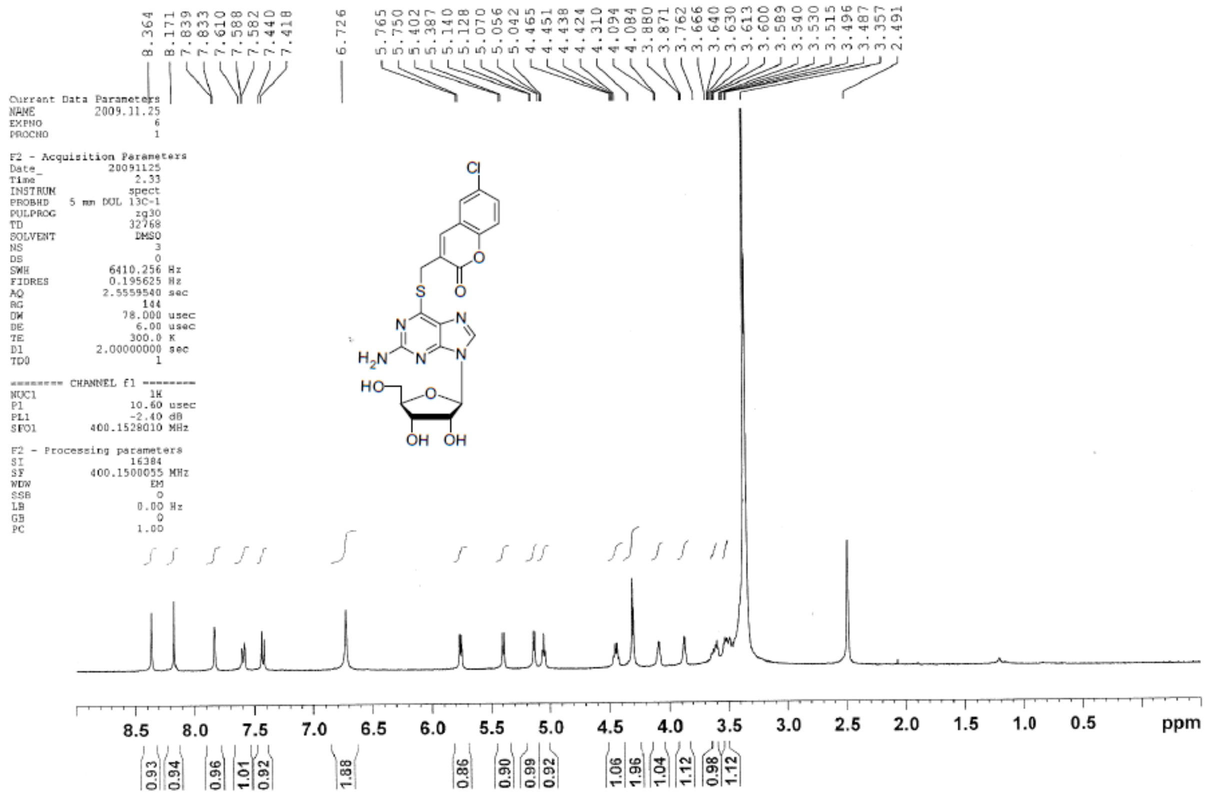

^{1}H NMR of Compound **9b**

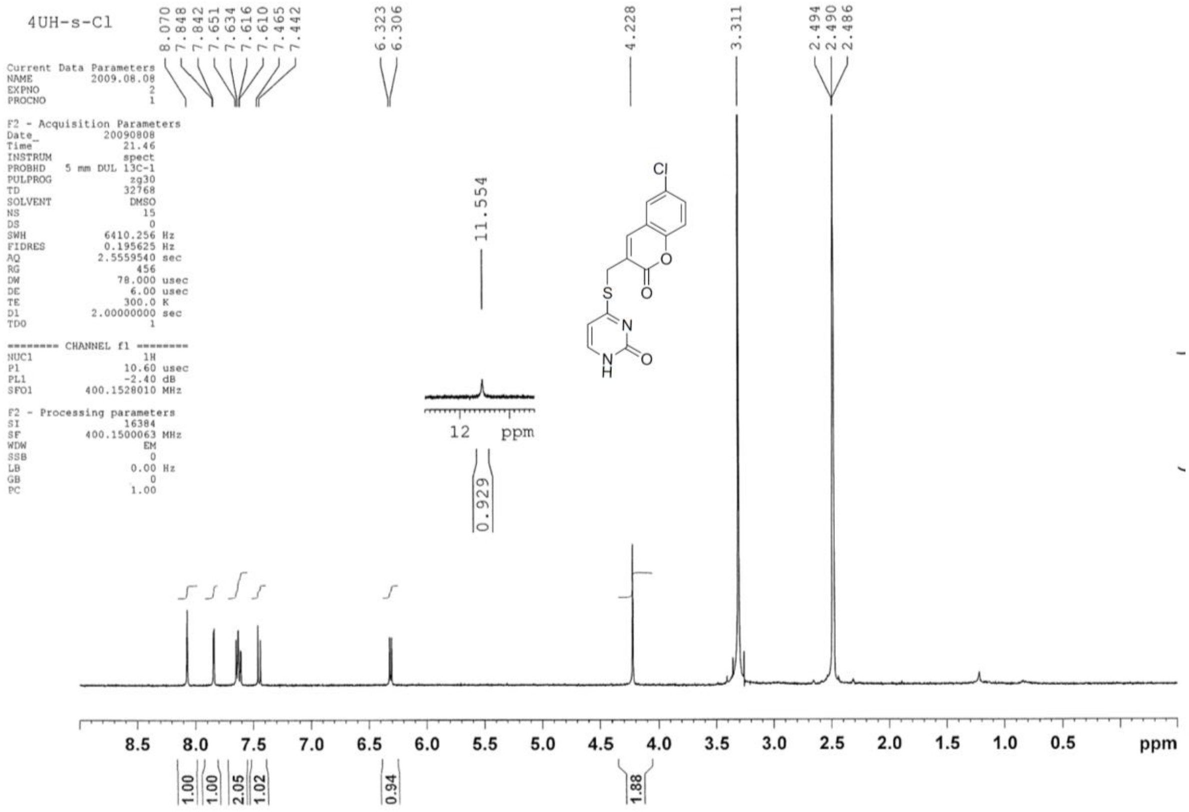

^{1}H NMR of Compound **11a**

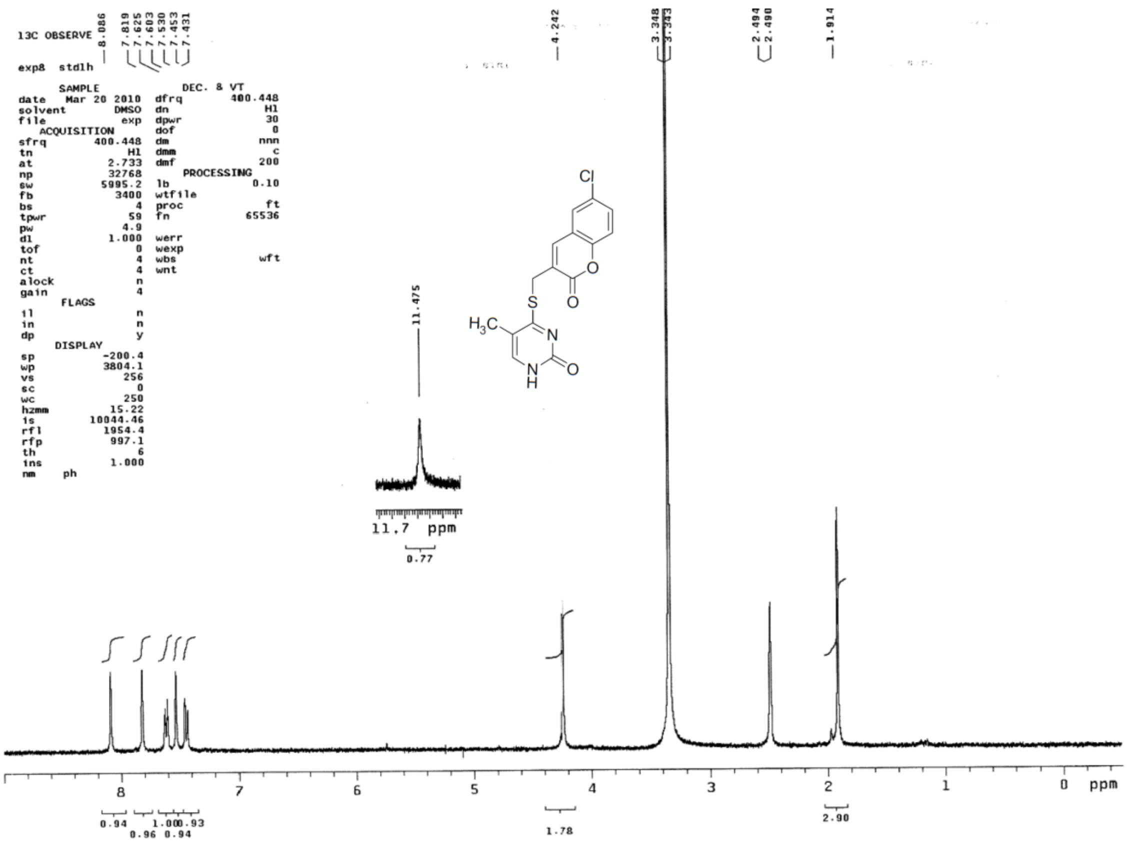

^{1}H NMR of Compound **11b**

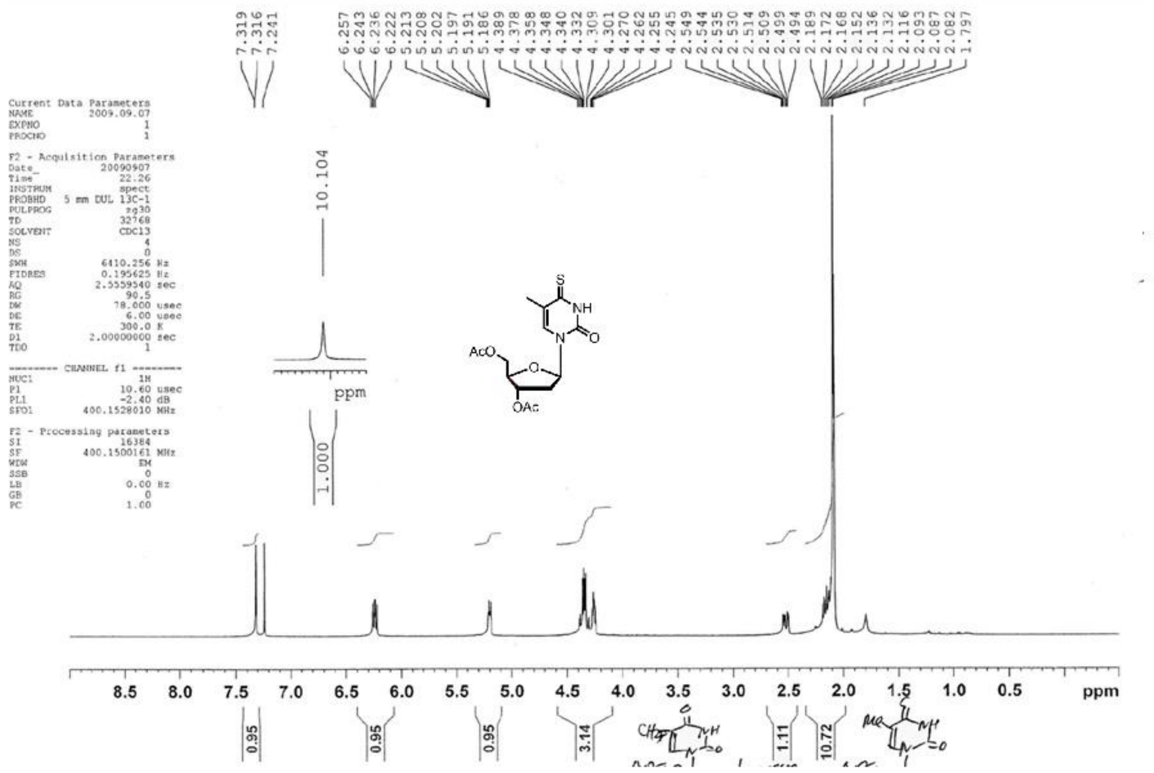

^{1}H NMR of Compound **14b**

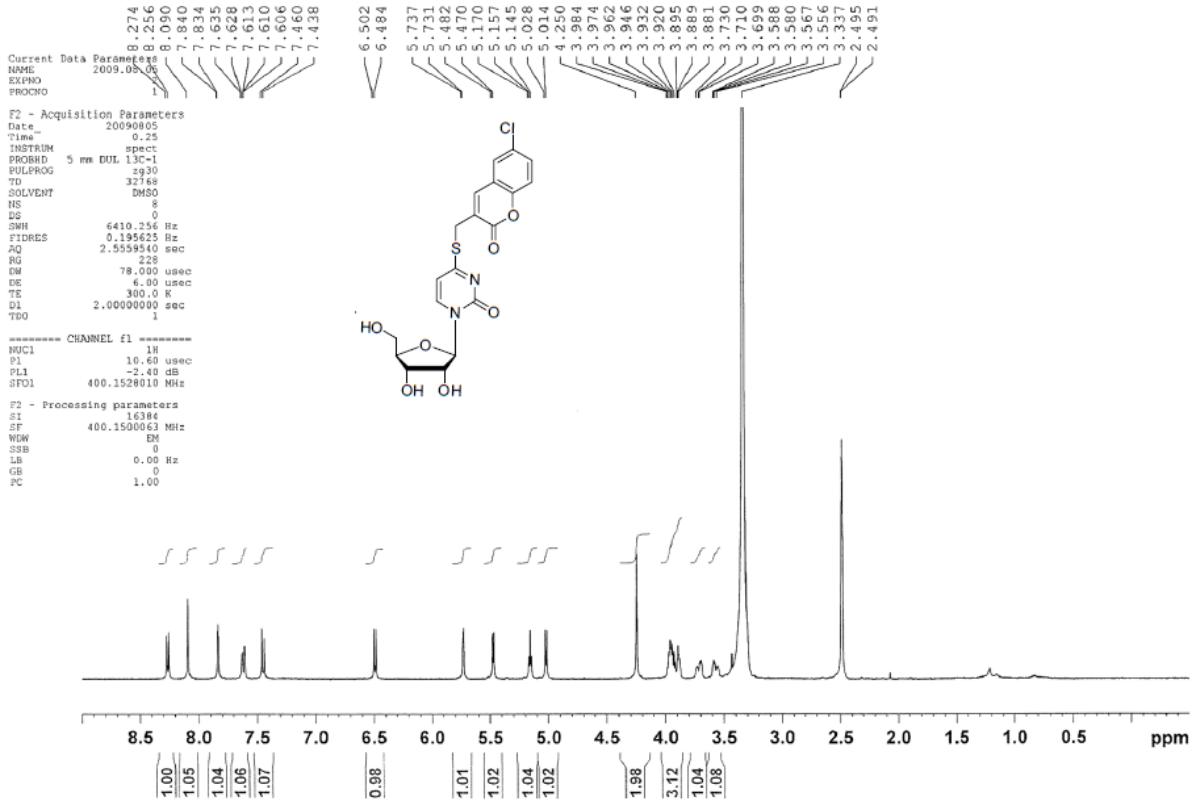

^{1}H NMR of Compound **16a**

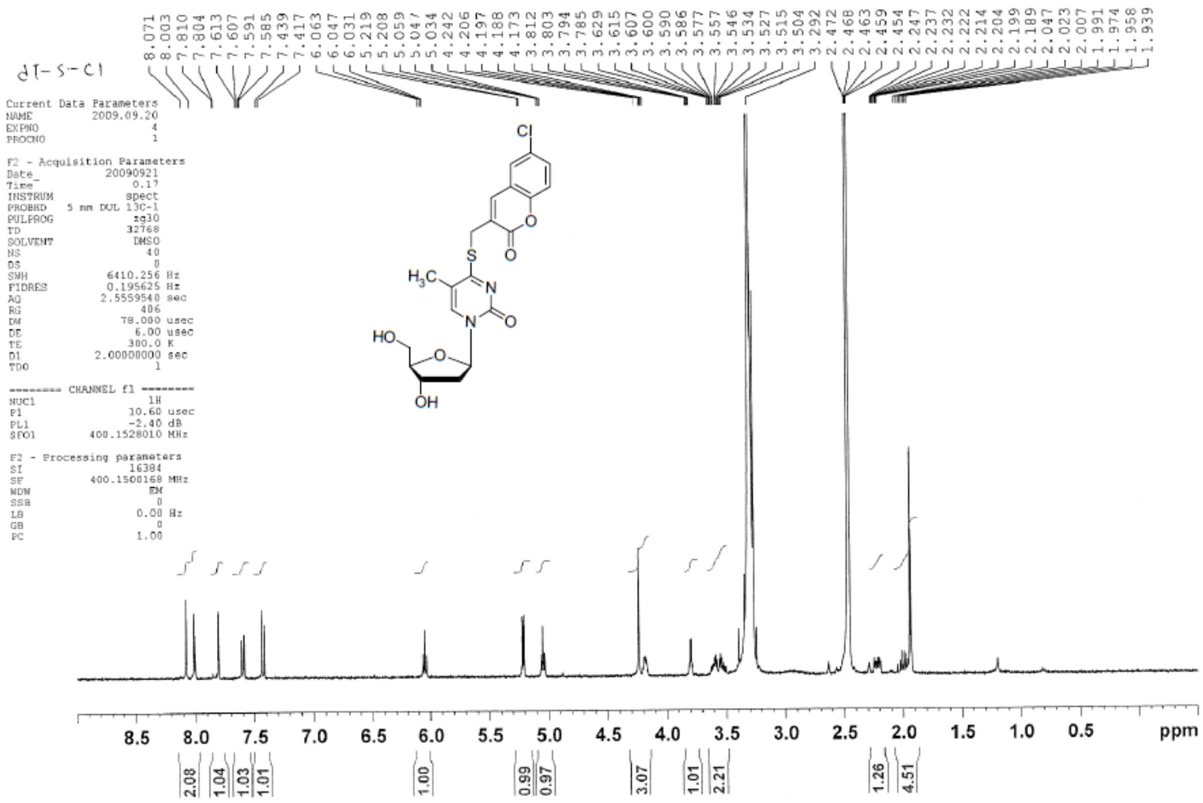

^{1}H NMR of Compound **16b**

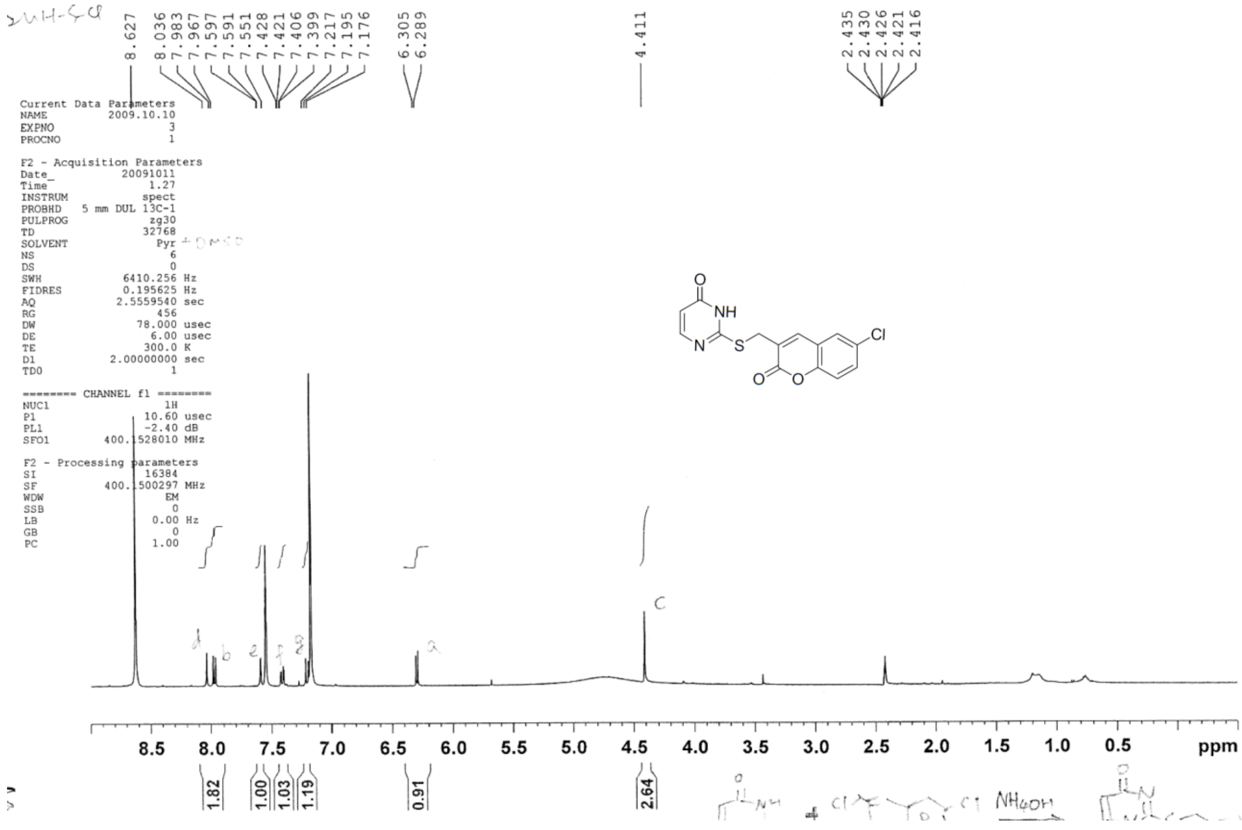

^{1}H NMR of Compound **18**

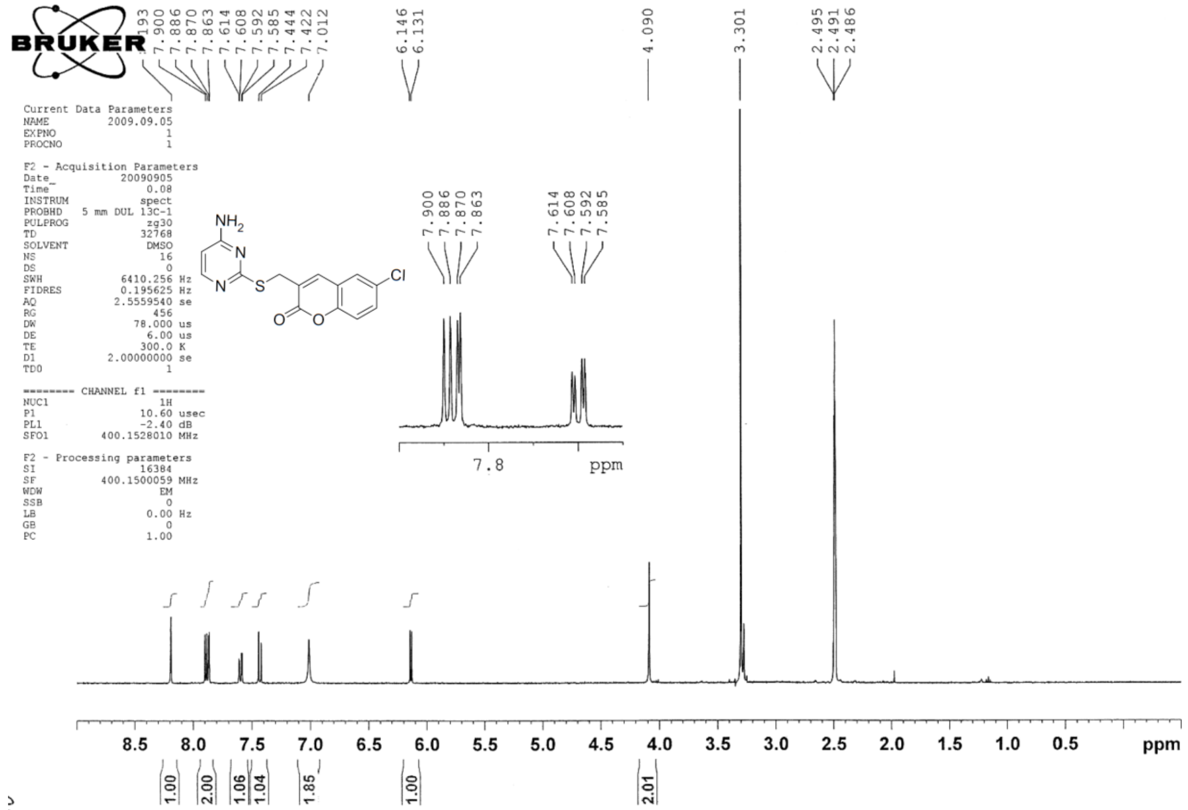

^{1}H NMR of Compound **20**

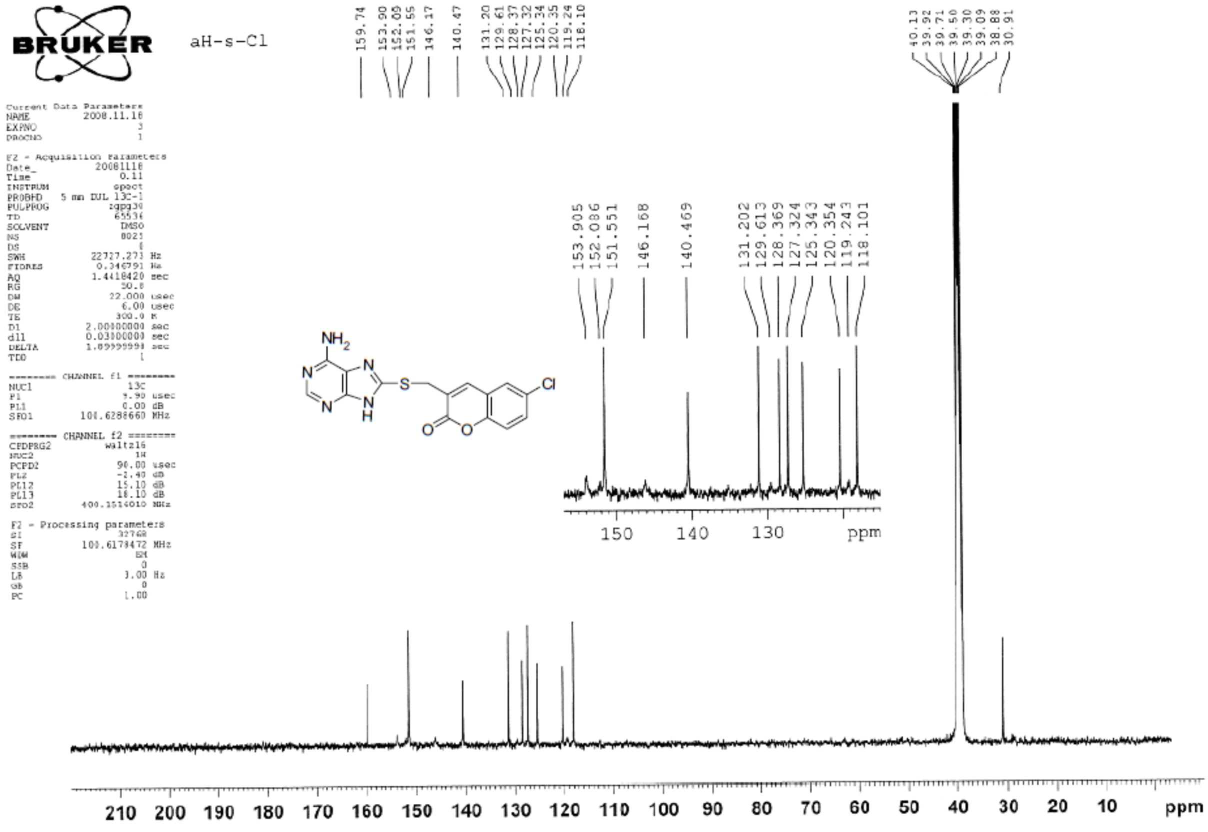

^{13}C NMR of Compound **3a**

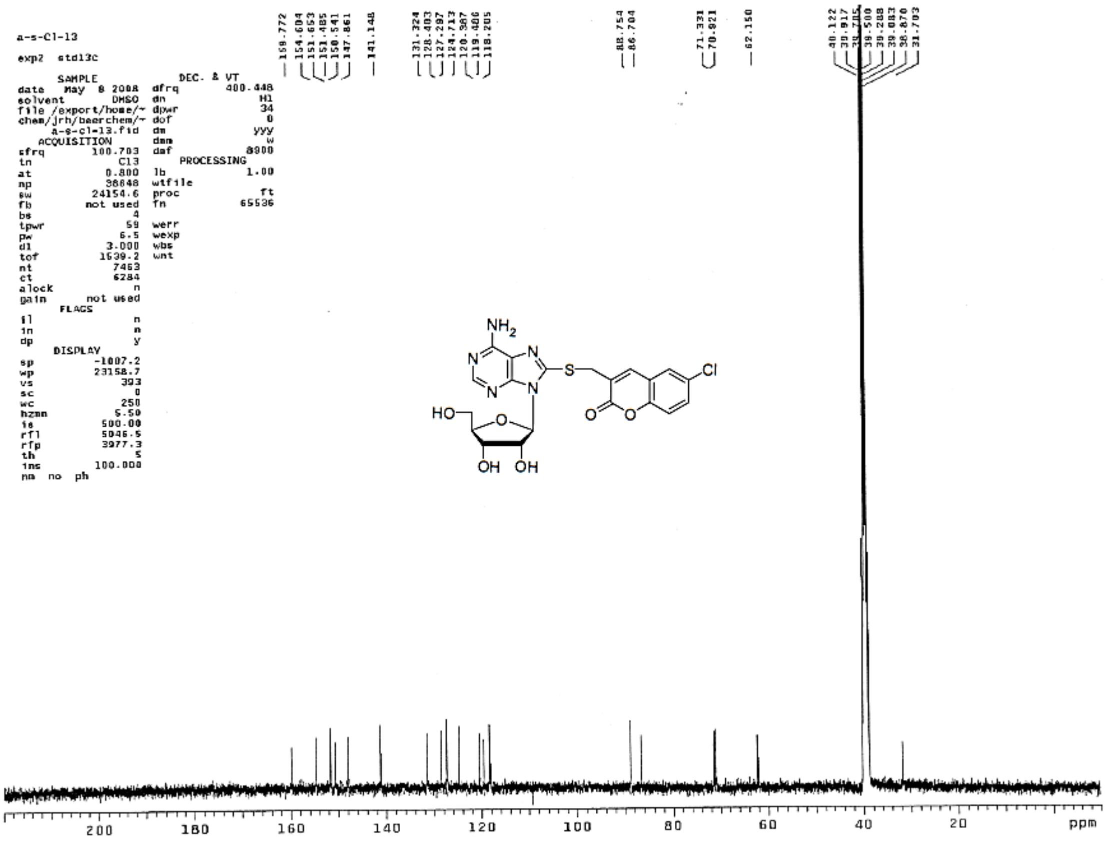

^{13}C NMR of Compound **3b**

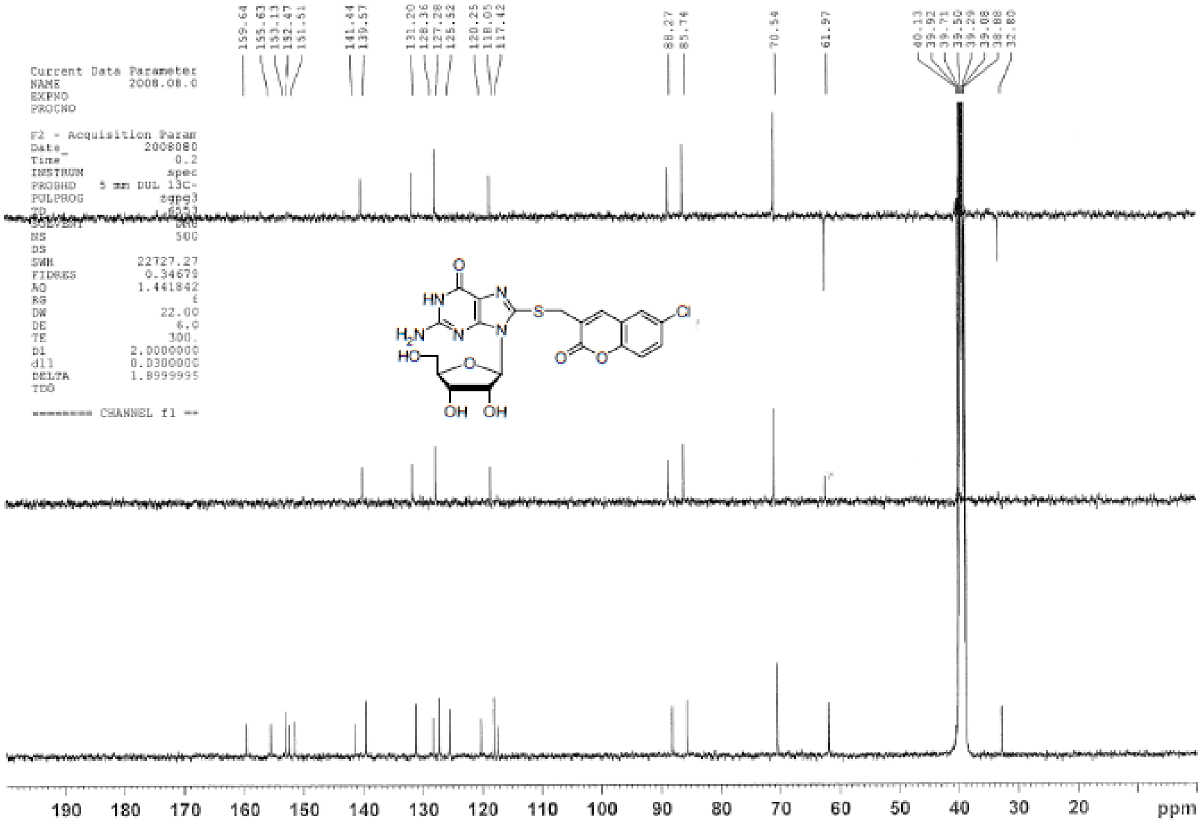

^{13}C NMR of Compound **5a**

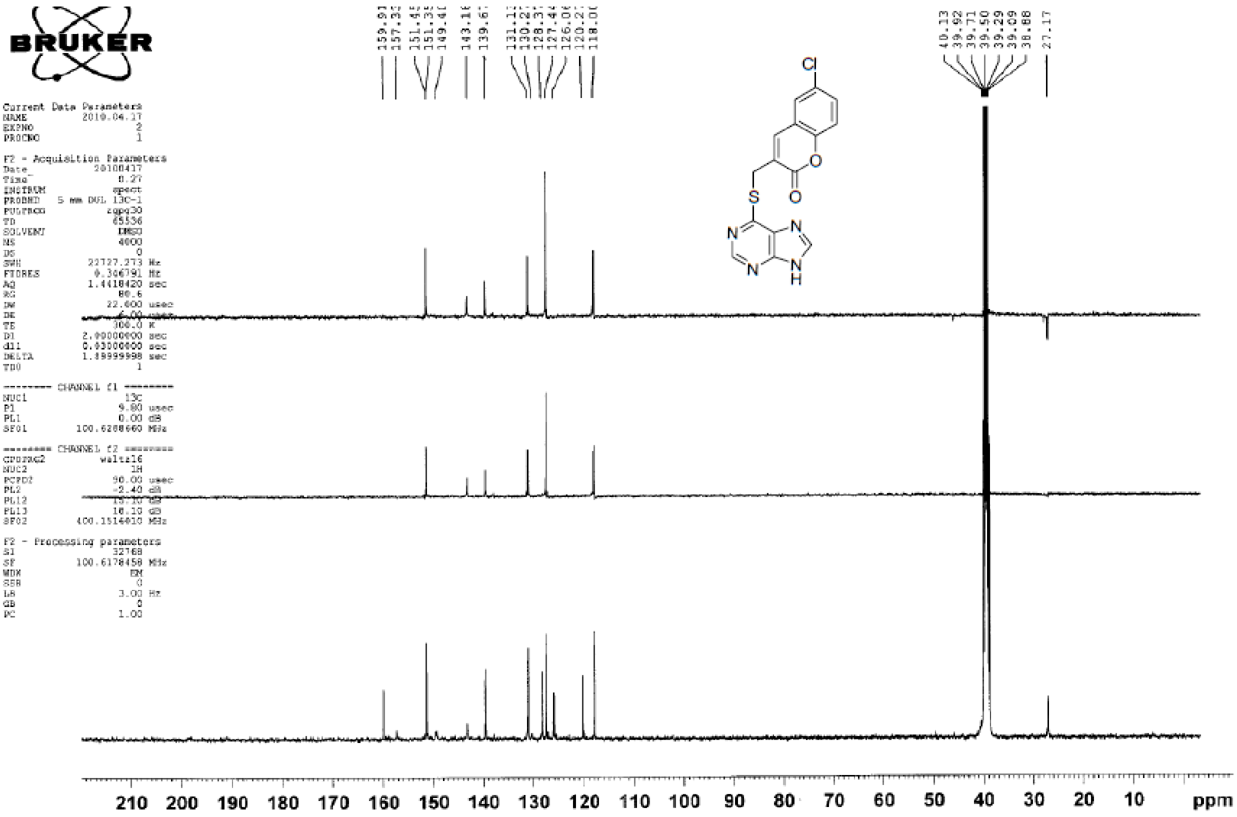

^{13}C NMR of Compound **7a**

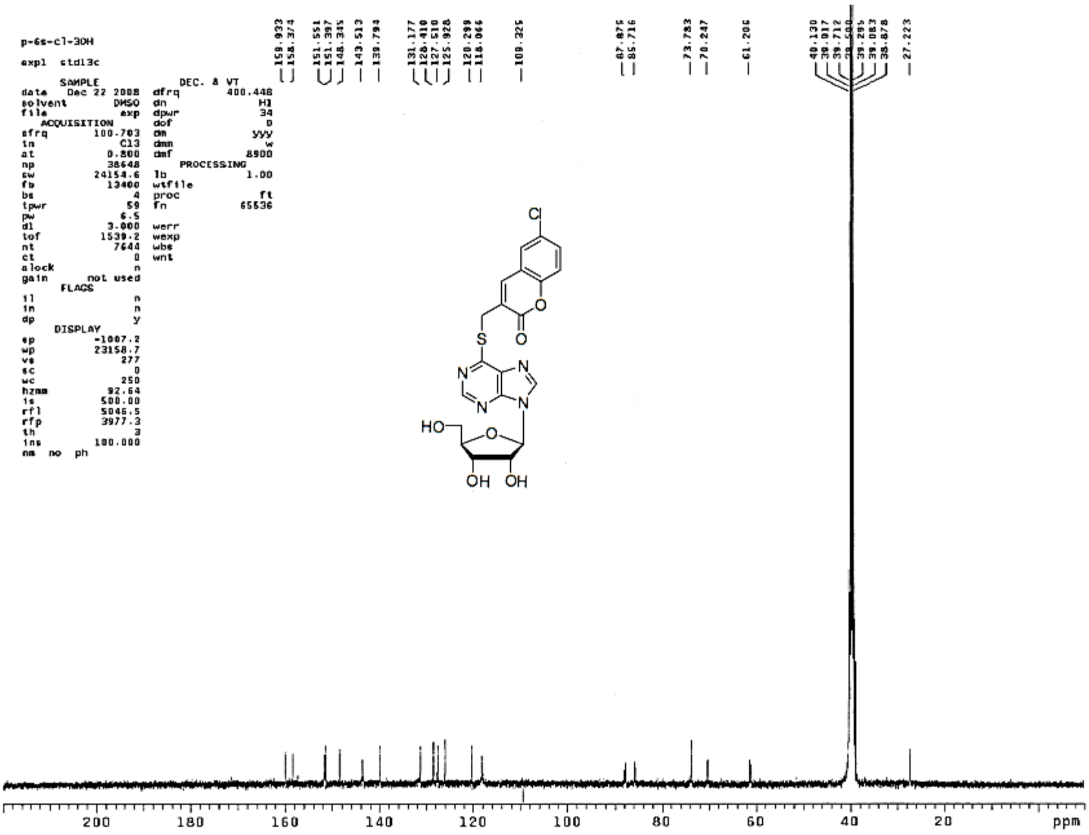

^{13}C NMR of Compound **7b**

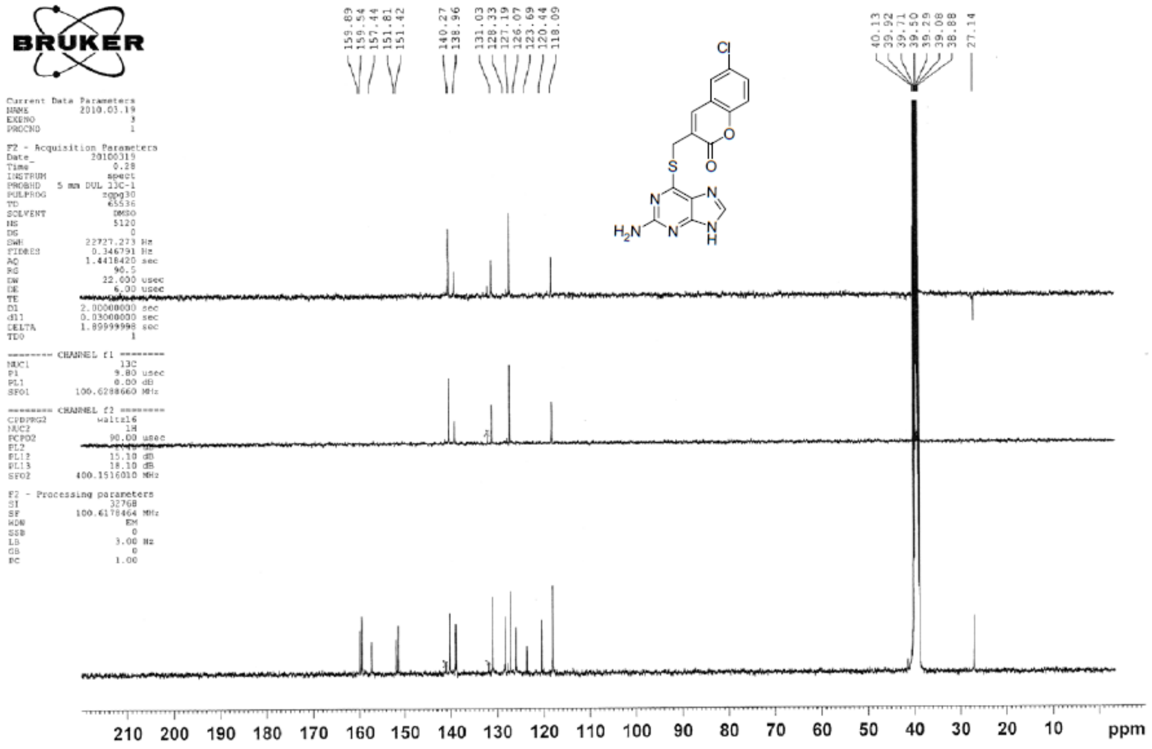

^{13}C NMR of Compound **9a**

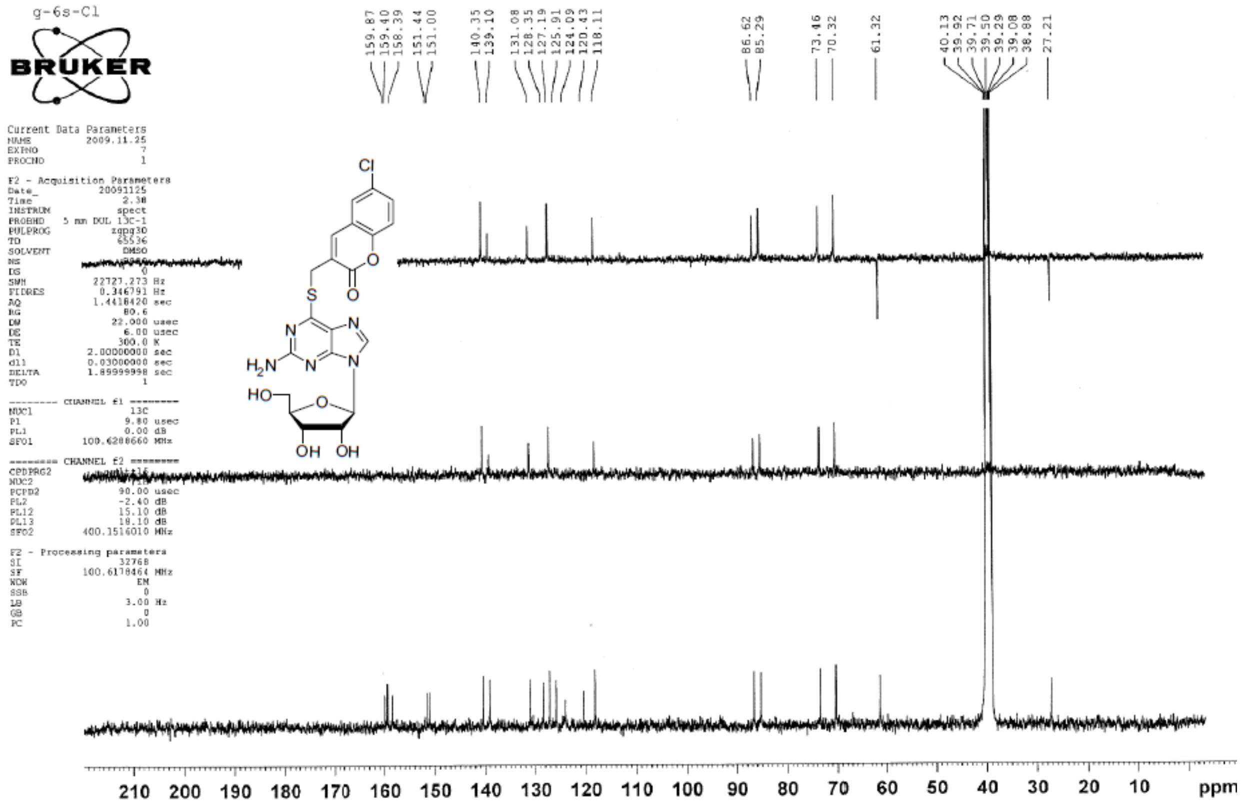

^{13}C NMR of Compound **9b**

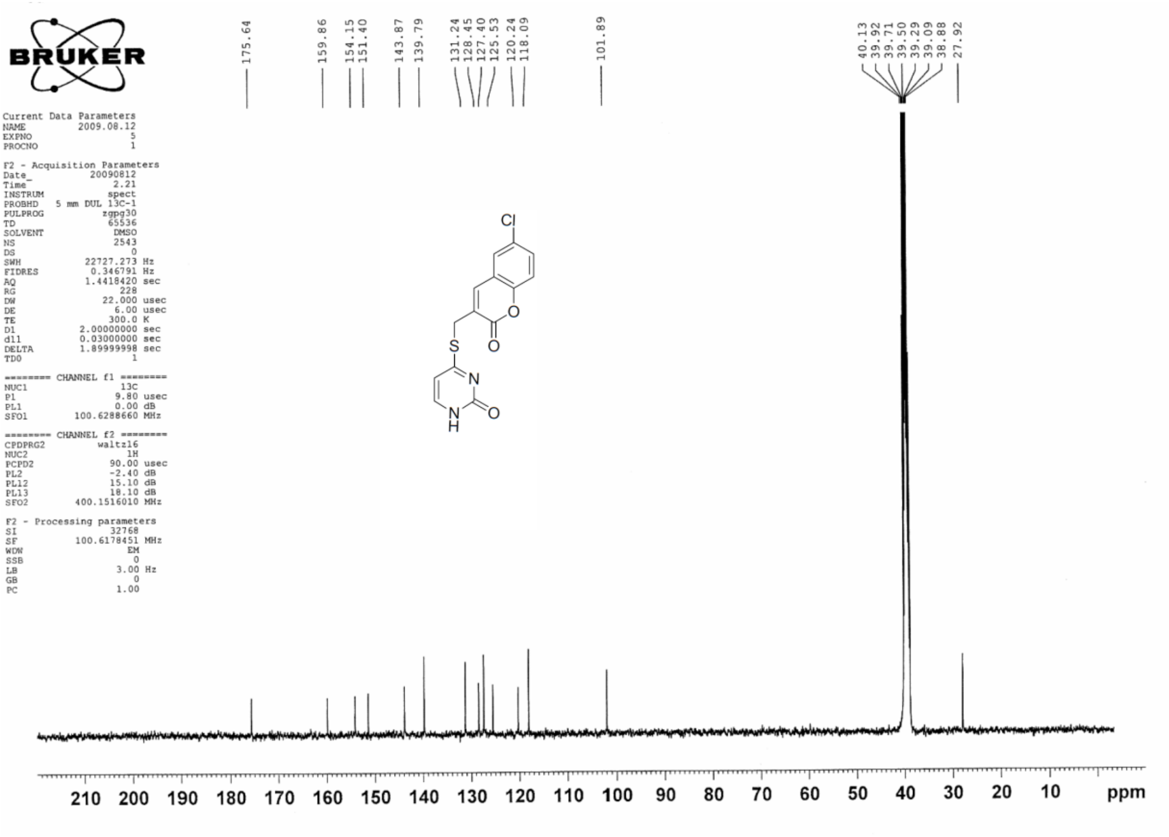

^{13}C NMR of Compound **11a**

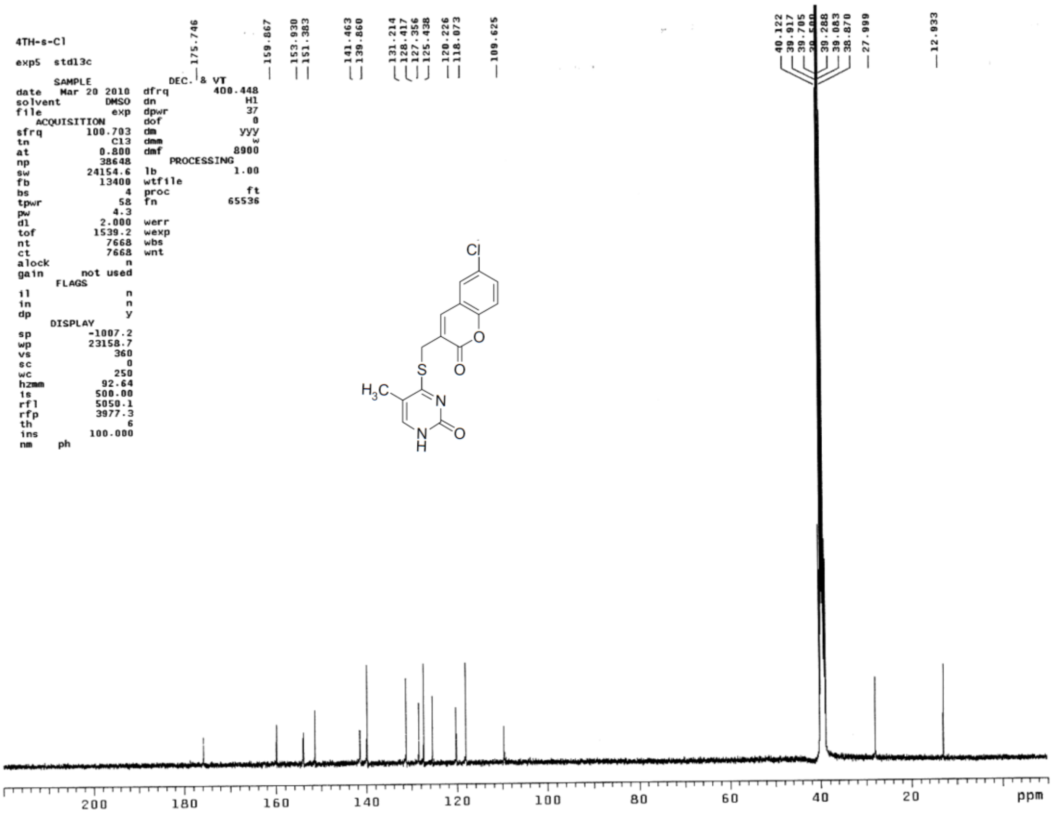

^{13}C NMR of Compound **11b**

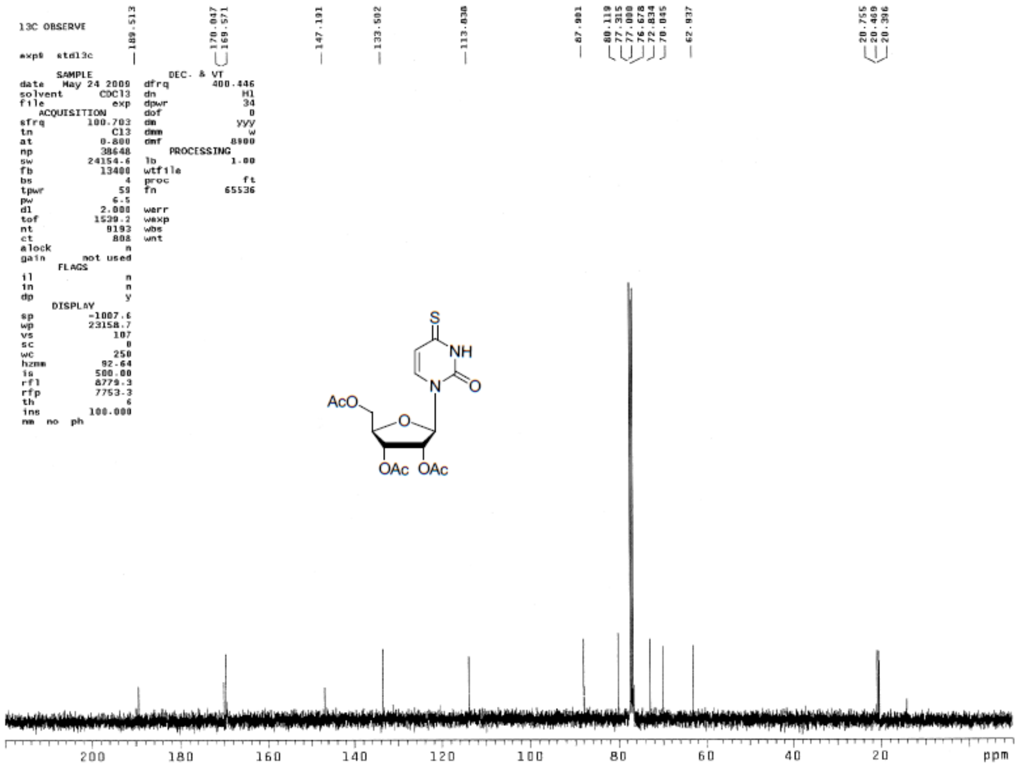

^{13}C NMR of Compound **14a**

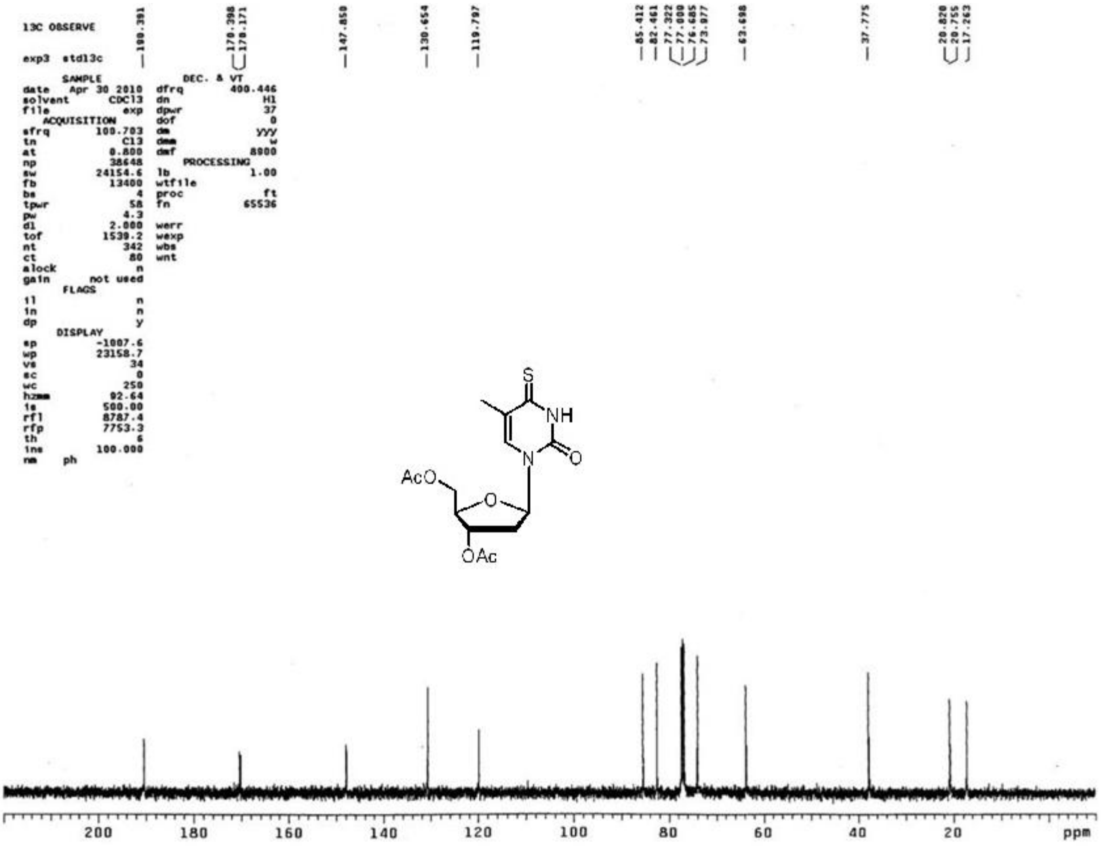

^{13}C NMR of Compound **14b**

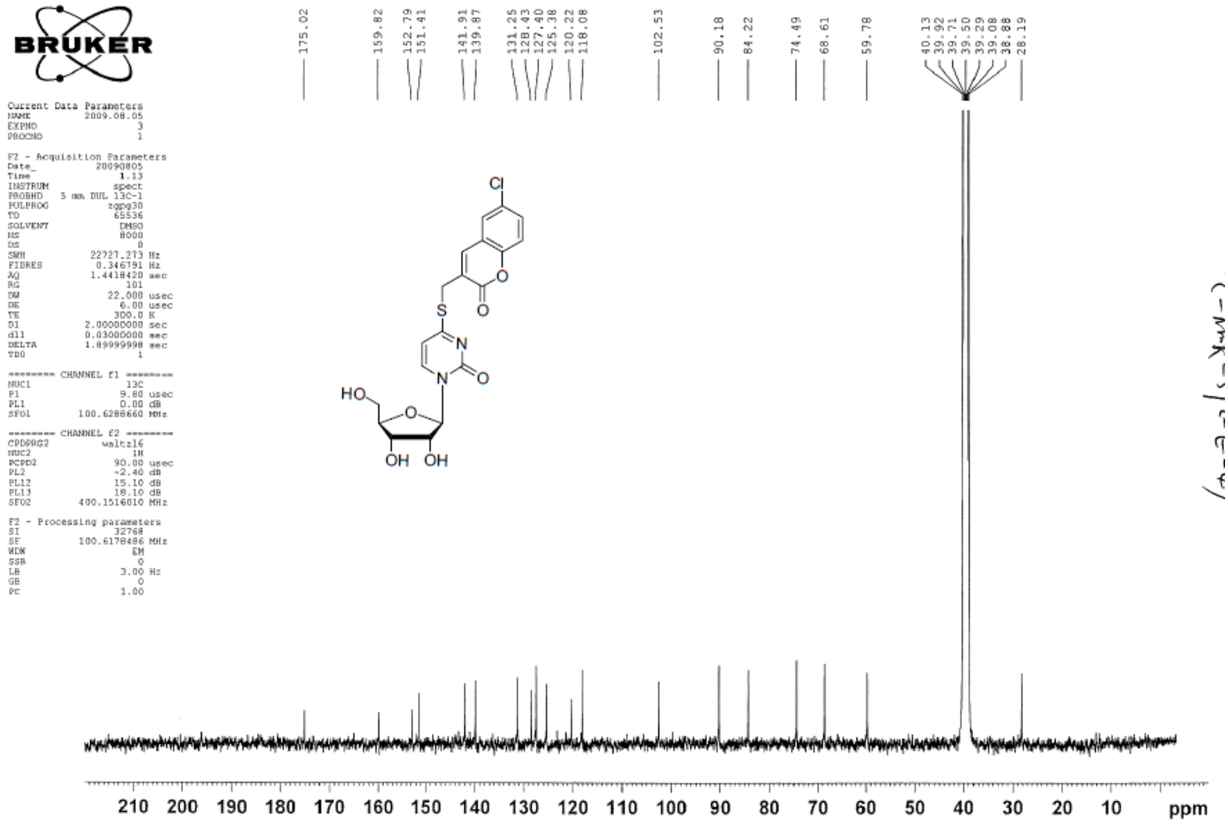

^{13}C NMR of Compound **16a**

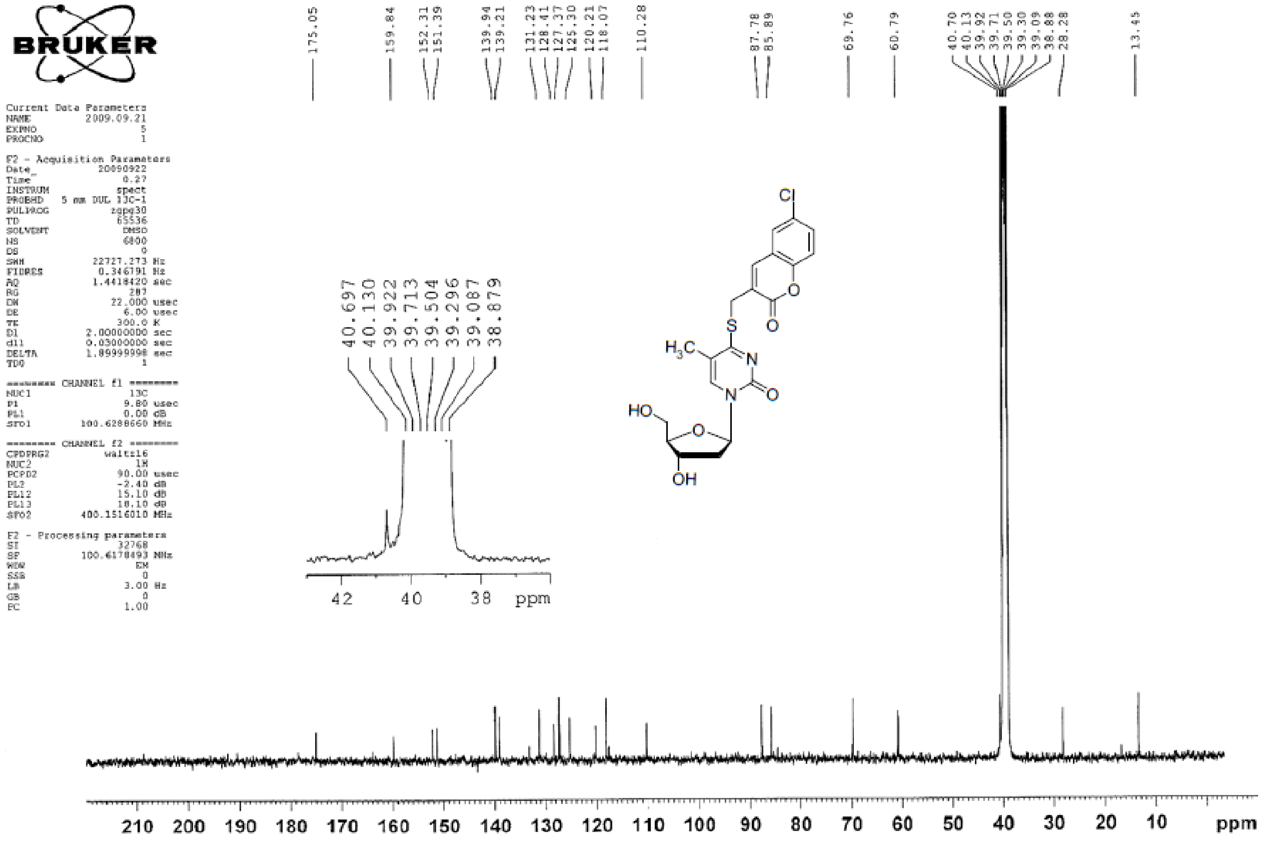

^{13}C NMR of Compound **16b**

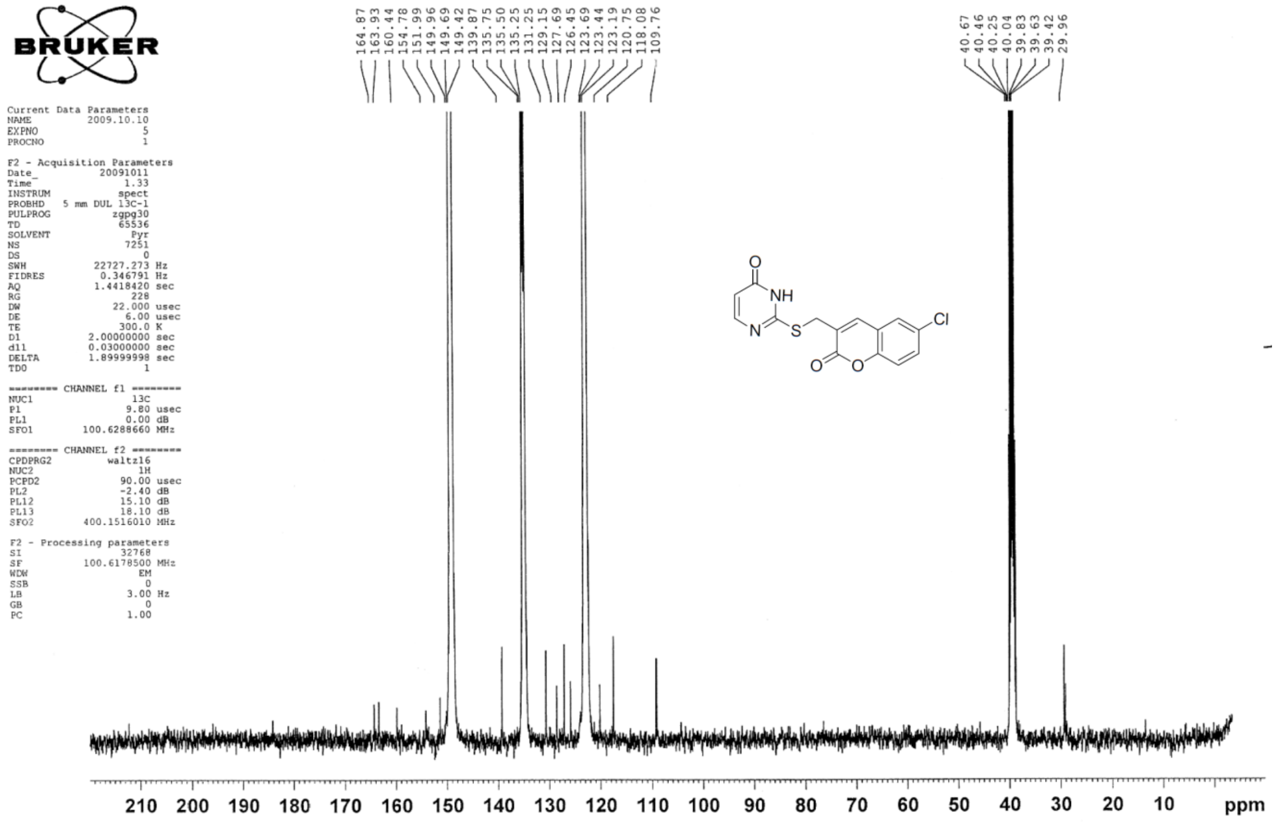

^{13}C NMR of Compound **18**

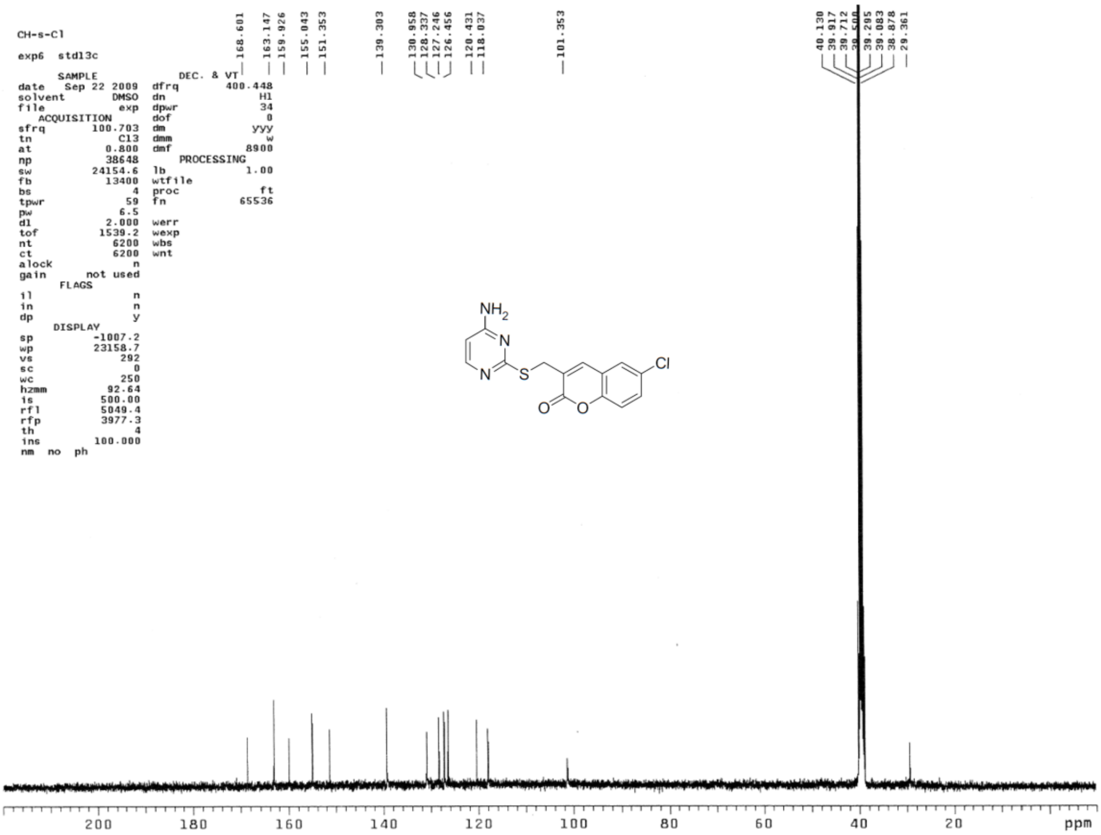

^{13}C NMR of Compound **20**

Charge number:1 Tolerance:500.00[ppm], 500.00 .. 500.... Unsaturation Number:-50.5 .. 50.0 (Fra...
Element:^{12}C:15 .. 15, ^{1}H:10 .. 11, ^{35}Cl:1 .. 1, ^{14}N:5 .. 5, ^{23}Na:0 .. 1, ^{16}O:2 .. 2, ^{32}S:1 .. 1

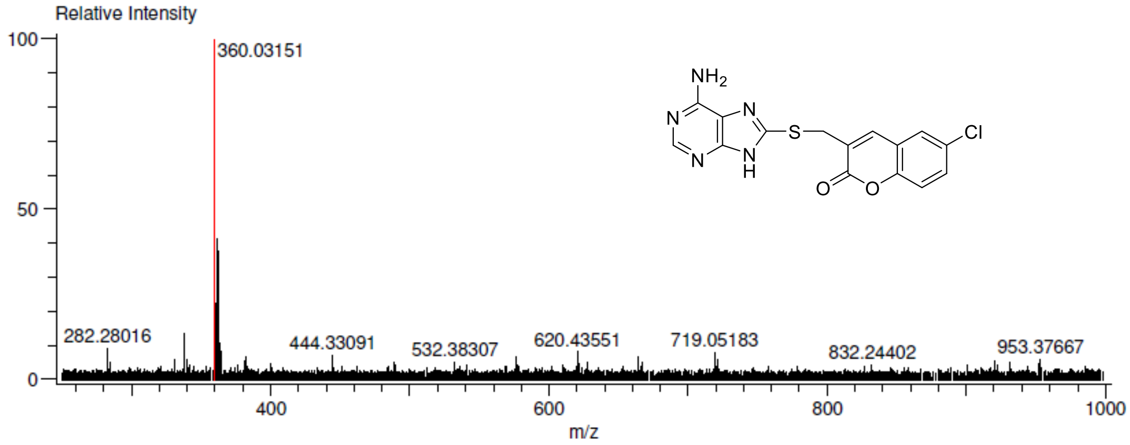

Mass	Intensity	Calc. Mass	Mass Difference [mDa]	Mass Difference [ppm]	Possible Formula
360.03151	103256.91	360.03220	-0.69	-1.91	$^{12}C_{15}{}^{1}H_{11}{}^{35}Cl_{1}{}^{14}N_{5}{}^{16}O_{2}{}^{32}S_{1}$

Mass spectrum of compound **3a**

Charge number:1 Tolerance:500.00[ppm], 500.00 .. 500.... Unsaturation Number:-50.5 .. 50.0 (Fra...
Element:^{12}C:20 .. 20, ^{1}H:18 .. 19, ^{35}Cl:1 .. 1, ^{14}N:5 .. 5, ^{16}O:6 .. 6, ^{32}S:1 .. 1

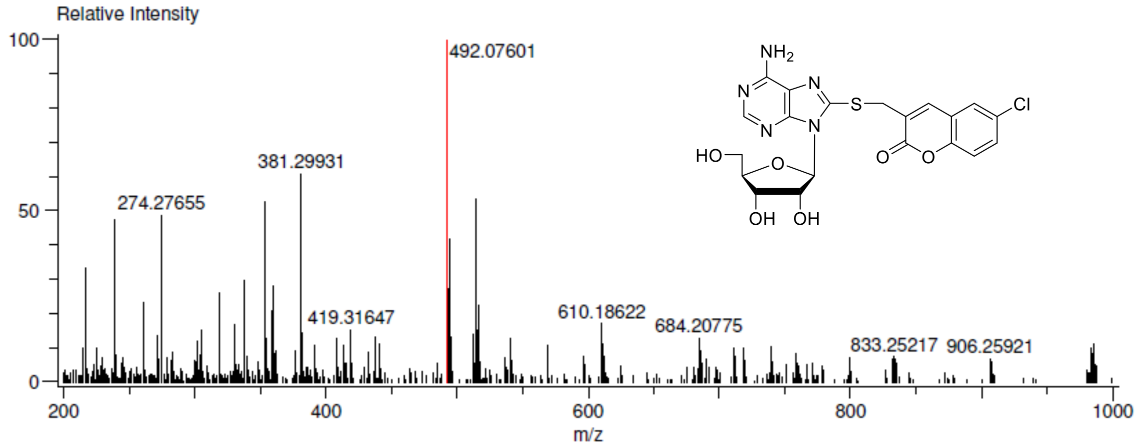

Mass	Intensity	Calc. Mass	Mass Difference [mDa]	Mass Difference [ppm]	Possible Formula
492.07601	31261.19	492.07446	1.55	3.15	$^{12}C_{20}{}^{1}H_{19}{}^{35}Cl_{1}{}^{14}N_{5}{}^{16}O_{6}{}^{32}S_{1}$

Mass spectrum of compound **3b**

Charge number:1 Tolerance:500.00[ppm], 500.00 .. 500.... Unsaturation Number:-50.5 .. 50.0 (Fra...
Element:^{12}C:20 .. 20, ^{1}H:18 .. 19, ^{35}Cl:1 .. 1, ^{14}N:5 .. 5, ^{23}Na:0 .. 1, ^{16}O:7 .. 7, ^{32}S:1 .. 1

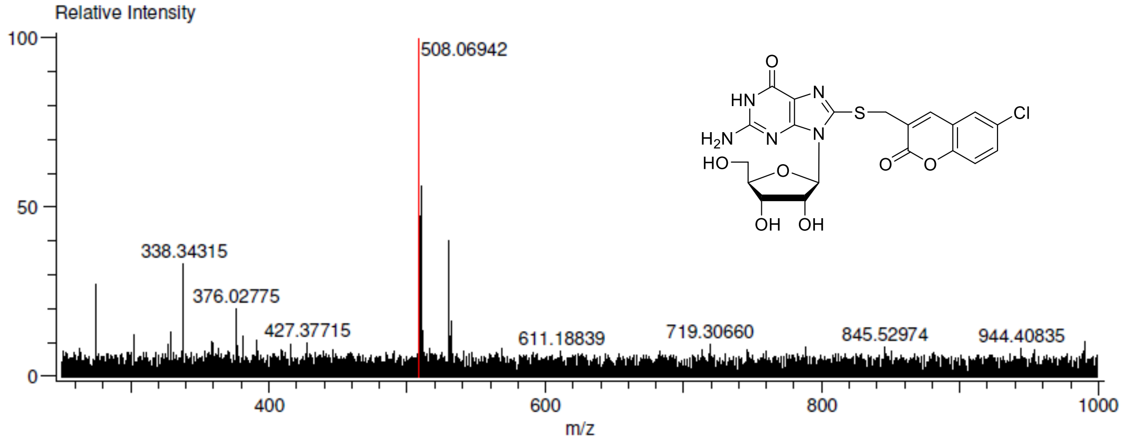

Mass	Intensity	Calc. Mass	Mass Difference [mDa]	Mass Difference [ppm]	Possible Formula
508.06942	27953.61	508.06937	0.05	0.10	$^{12}C_{20}{}^{1}H_{19}{}^{35}Cl_{1}{}^{14}N_{5}{}^{16}O_{7}{}^{32}S_{1}$

Mass spectrum of compound **5a**

Charge number:1 Tolerance:500.00[ppm], 500.00 .. 500.... Unsaturation Number:-50.5 .. 50.0 (Fra...
Element:^{12}C:15 .. 15, ^{1}H:10 .. 11, ^{35}Cl:1 .. 1, ^{14}N:5 .. 5, ^{23}Na:0 .. 1, ^{16}O:3 .. 3, ^{32}S:1 .. 1

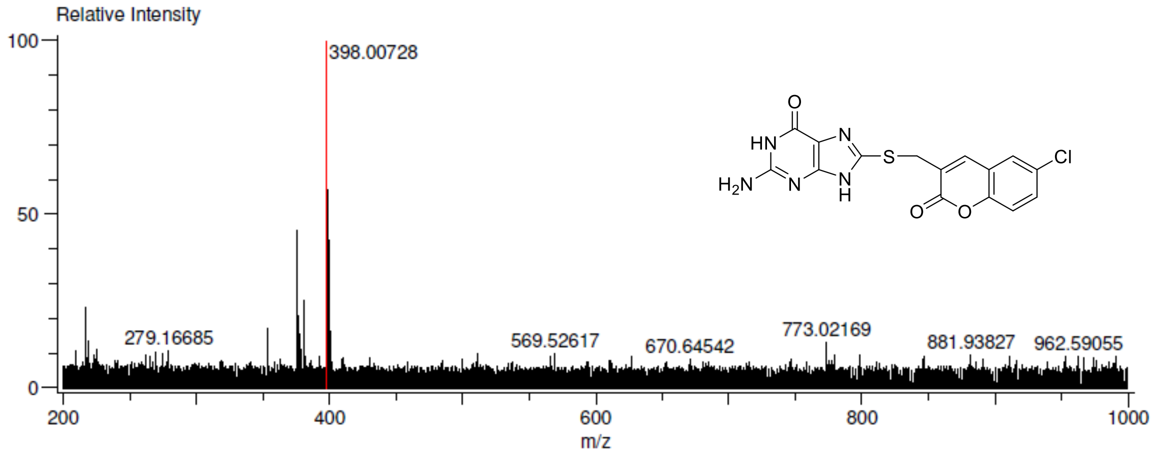

Mass	Intensity	Calc. Mass	Mass Difference [mDa]	Mass Difference [ppm]	Possible Formula
398.00728	35766.85	398.00906	-1.77	-4.45	$^{12}C_{15}{}^{1}H_{10}{}^{35}Cl_{1}{}^{14}N_{5}{}^{23}Na_{1}{}^{16}O_{3}{}^{32}S_{1}$

Mass spectrum of compound **5b**

Charge number:1 Tolerance:500.00[ppm], 500.00 .. 500.... Unsaturation Number:-50.5 .. 50.0 (Fra...
Element:^{12}C:15 .. 15, ^{1}H:9 .. 10, ^{35}Cl:1 .. 1, ^{14}N:4 .. 4, ^{23}Na:0 .. 1, ^{16}O:2 .. 2, ^{32}S:1 .. 1

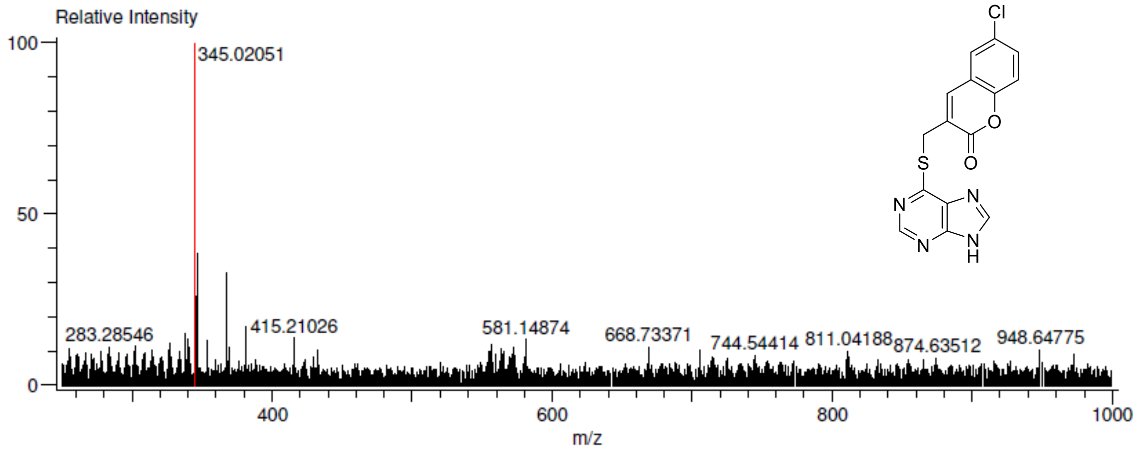

Mass	Intensity	Calc. Mass	Mass Difference [mDa]	Mass Difference [ppm]	Possible Formula
345.02051	40418.50	345.02130	-0.79	-2.28	$^{12}C_{15}{}^{1}H_{10}{}^{35}Cl_{1}{}^{14}N_{4}{}^{16}O_{2}{}^{32}S_{1}$

Mass spectrum of compound **7a**

Charge number:1 Tolerance:500.00[ppm], 500.00 .. 500.... Unsaturation Number:-50.5 .. 50.0 (Fra...
Element:^{12}C:20 .. 20, ^{1}H:17 .. 18, ^{35}Cl:1 .. 1, ^{14}N:4 .. 4, ^{23}Na:0 .. 1, ^{16}O:6 .. 6, ^{32}S:1 .. 1

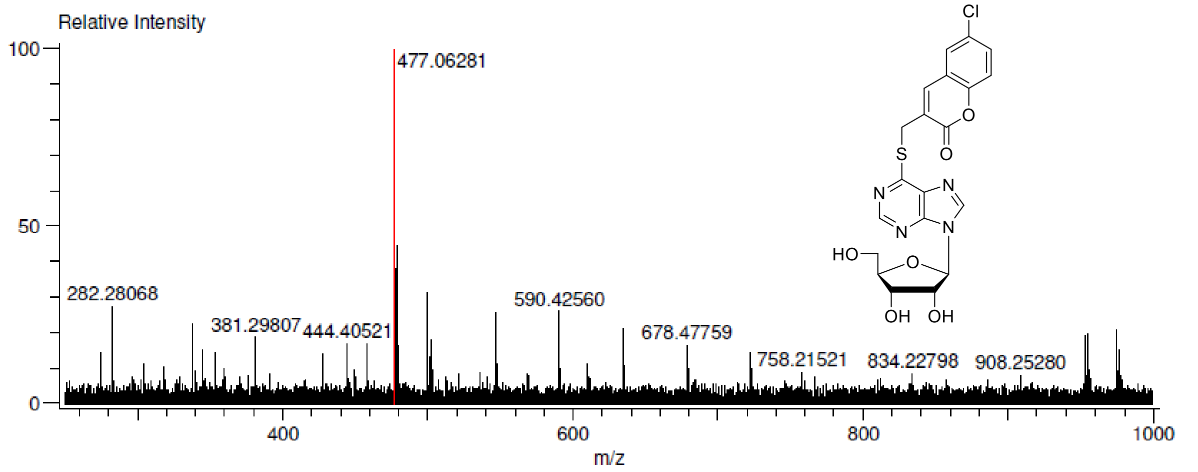

Mass	Intensity	Calc. Mass	Mass Difference [mDa]	Mass Difference [ppm]	Possible Formula
477.06281	29344.80	477.06356	-0.74	-1.56	$^{12}C_{20}{}^{1}H_{18}{}^{35}Cl_{1}{}^{14}N_{4}{}^{16}O_{6}{}^{32}S_{1}$

Mass spectrum of compound **7b**

Charge number:1 Tolerance:500.00[ppm], 500.00 .. 500.... Unsaturation Number:-50.5 .. 50.0 (Fra...

Element:^{12}C:15 .. 15, ^{1}H:10 .. 11, ^{35}Cl:1 .. 1, ^{14}N:5 .. 5, ^{23}Na:0 .. 1, ^{16}O:2 .. 2, ^{32}S:1 .. 1

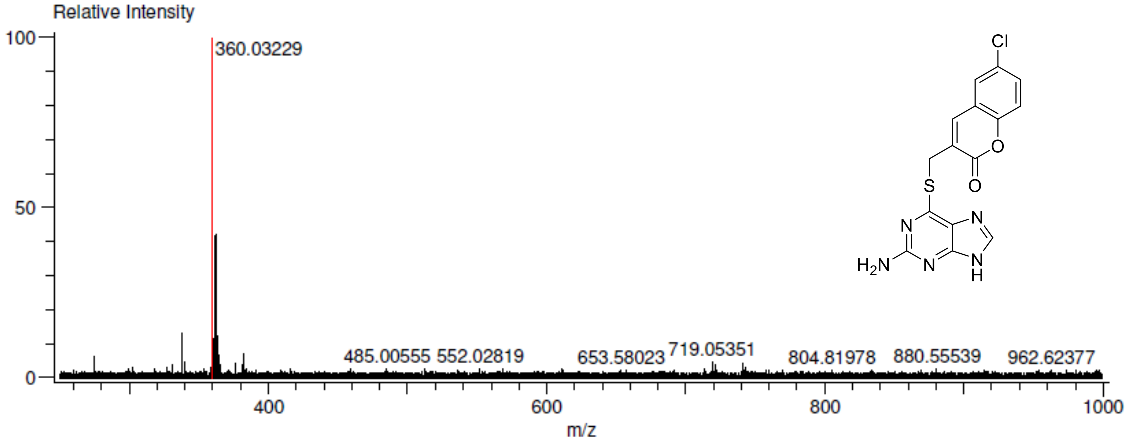

Mass	Intensity	Calc. Mass	Mass Difference [mDa]	Mass Difference [ppm]	Possible Formula
360.03229	107304.52	360.03220	0.09	0.26	$^{12}C_{15}{}^{1}H_{11}{}^{35}Cl_{1}{}^{14}N_{5}{}^{16}O_{2}{}^{32}S_{1}$

Mass spectrum of compound **9a**

Charge number:1 Tolerance:500.00[ppm], 500.00 .. 500.... Unsaturation Number:-50.5 .. 50.0 (Fra...

Element:^{12}C:20 .. 20, ^{1}H:18 .. 19, ^{35}Cl:1 .. 1, ^{14}N:5 .. 5, ^{23}Na:0 .. 1, ^{16}O:6 .. 6, ^{32}S:1 .. 1

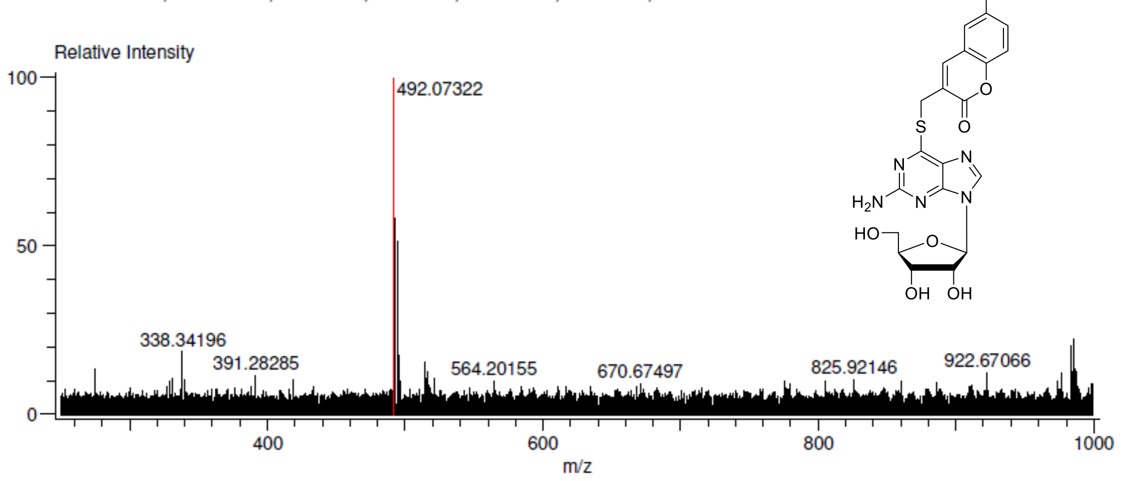

Mass	Intensity	Calc. Mass	Mass Difference [mDa]	Mass Difference [ppm]	Possible Formula
492.07322	34159.15	492.07446	-1.23	-2.50	$^{12}C_{20}{}^{1}H_{19}{}^{35}Cl_{1}{}^{14}N_{5}{}^{16}O_{6}{}^{32}S_{1}$

Mass spectrum of compound **9b**

Charge number:1 Tolerance:500.00[ppm], 500.00 .. 500.... Unsaturation Number:-50.5 .. 50.0 (Fra...
Element:^{12}C:14 .. 14, ^{1}H:9 .. 10, ^{35}Cl:1 .. 1, ^{14}N:2 .. 2, ^{16}O:3 .. 3, ^{32}S:1 .. 1

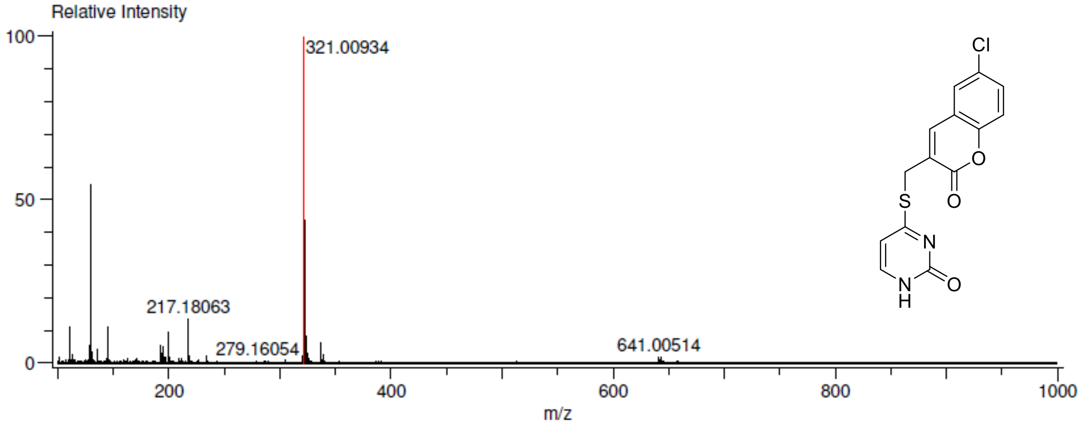

Mass	Intensity	Calc. Mass	Mass Difference [mDa]	Mass Difference [ppm]	Possible Formula
321.00934	3634961.75	321.01007	-0.72	-2.25	$^{12}C_{14}{}^{1}H_{10}{}^{35}Cl_{1}{}^{14}N_{2}{}^{16}O_{3}{}^{32}S_{1}$

Mass spectrum of compound **11a**

Charge number:1 Tolerance:500.00[ppm], 500.00 .. 500.... Unsaturation Number:-50.5 .. 50.0 (Fra...
Element:^{12}C:15 .. 15, ^{1}H:11 .. 12, ^{35}Cl:1 .. 1, ^{14}N:2 .. 2, ^{23}Na:0 .. 1, ^{16}O:3 .. 3, ^{32}S:1 .. 1

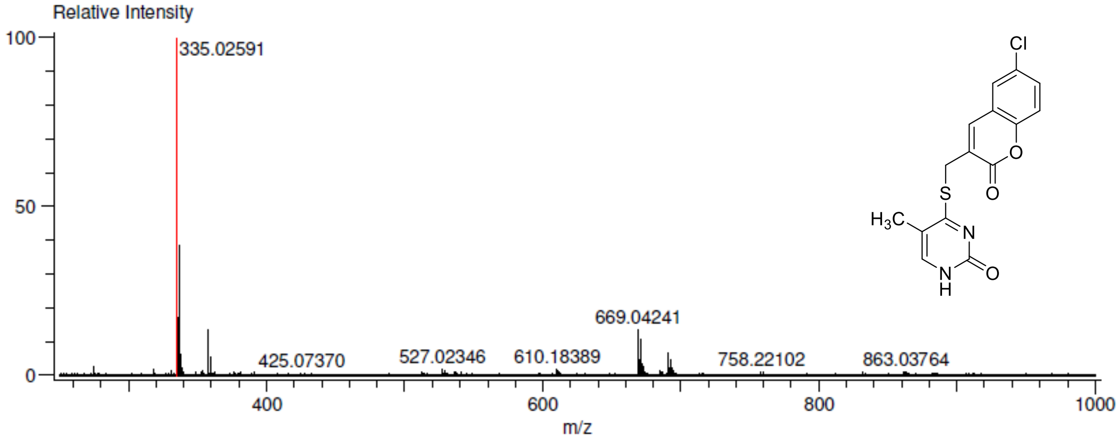

Mass	Intensity	Calc. Mass	Mass Difference [mDa]	Mass Difference [ppm]	Possible Formula
335.02591	222492.09	335.02572	0.20	0.60	$^{12}C_{15}{}^{1}H_{12}{}^{35}Cl_{1}{}^{14}N_{2}{}^{16}O_{3}{}^{32}S_{1}$

Mass spectrum of compound **11b**

Charge number:1 Tolerance:500.00[ppm], 500.00 .. 500.... Unsaturation Number:-50.5 .. 50.0 (Fra...
Element:^{12}C:19 .. 19, ^{1}H:17 .. 18, ^{35}Cl:1 .. 1, ^{14}N:2 .. 2, ^{23}Na:0 .. 1, ^{16}O:7 .. 7, ^{32}S:1 .. 1

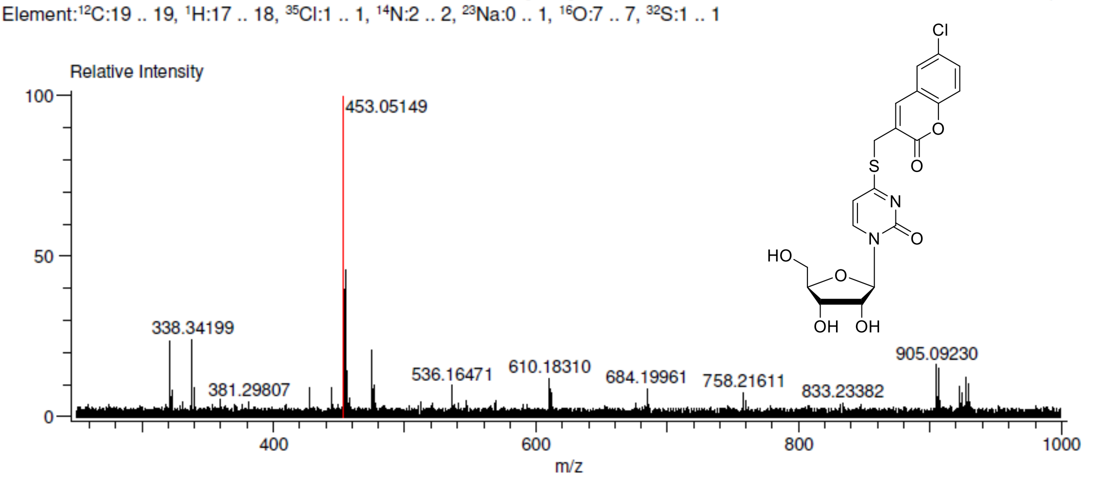

Mass	Intensity	Calc. Mass	Mass Difference [mDa]	Mass Difference [ppm]	Possible Formula
453.05149	57672.98	453.05232	-0.83	-1.84	$^{12}C_{19}{}^{1}H_{18}{}^{35}Cl_{1}{}^{14}N_{2}{}^{16}O_{7}{}^{32}S_{1}$

Mass spectrum of compound **16a**

Charge number:1 Tolerance:500.00[ppm], 500.00 .. 500.... Unsaturation Number:-50.5 .. 50.0 (Fra...
Element:^{12}C:20 .. 20, ^{1}H:19 .. 20, ^{35}Cl:1 .. 1, ^{14}N:2 .. 2, ^{23}Na:0 .. 1, ^{16}O:6 .. 6, ^{32}S:1 .. 1

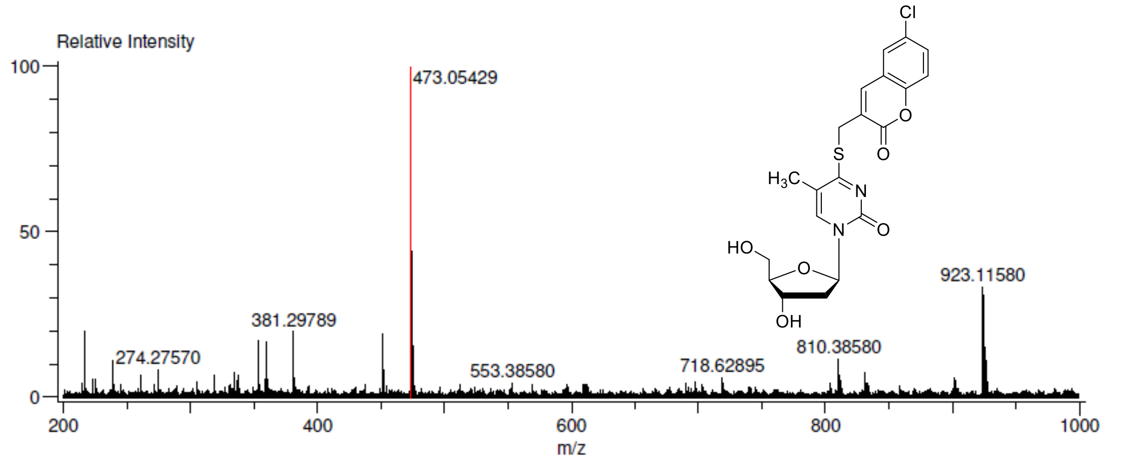

Mass	Intensity	Calc. Mass	Mass Difference [mDa]	Mass Difference [ppm]	Possible Formula
473.05429	64997.17	473.05500	-0.71	-1.50	$^{12}C_{20}{}^{1}H_{19}{}^{35}Cl_{1}{}^{14}N_{2}{}^{23}Na_{1}{}^{16}O_{6}{}^{32}S_{1}$

Mass spectrum of compound **16b**

Charge number:1 Tolerance:500.00[ppm], 500.00 .. 500.... Unsaturation Number:-50.5 .. 50.0 (Fra...

Element:^{12}C:14 .. 14, ^{1}H:9 .. 10, ^{35}Cl:1 .. 1, ^{14}N:2 .. 2, ^{23}Na:0 .. 1, ^{16}O:3 .. 3, ^{32}S:1 .. 1

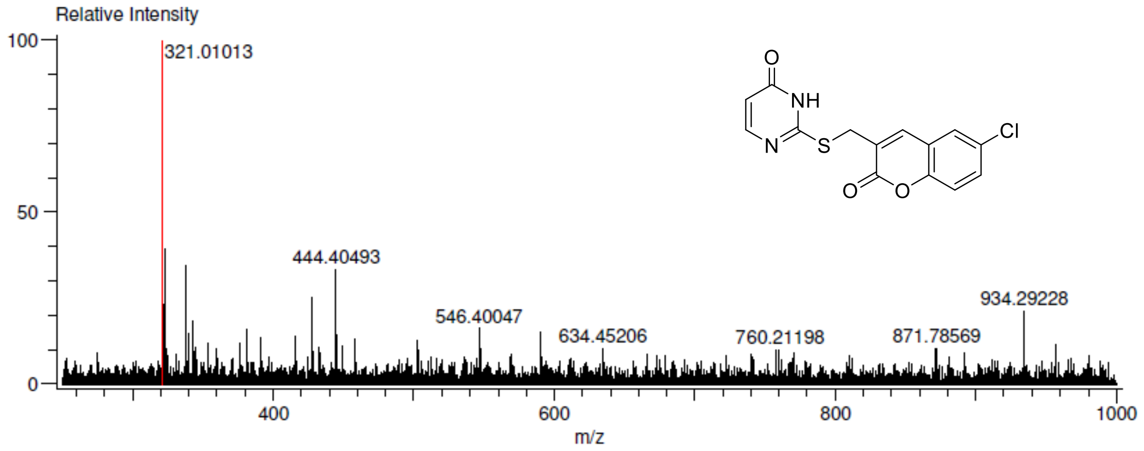

Mass	Intensity	Calc. Mass	Mass Difference [mDa]	Mass Difference [ppm]	Possible Formula
321.01013	48122.46	321.01007	0.06	0.20	$^{12}C_{14}{}^{1}H_{10}{}^{35}Cl_1{}^{14}N_2{}^{16}O_3{}^{32}S_1$

Mass spectrum of compound **18**

Charge number:1 Tolerance:500.00[ppm], 500.00 .. 500.... Unsaturation Number:-50.5 .. 50.0 (Fra...

Element:^{12}C:14 .. 14, ^{1}H:10 .. 11, ^{35}Cl:1 .. 1, ^{14}N:3 .. 3, ^{16}O:2 .. 2, ^{32}S:1 .. 1

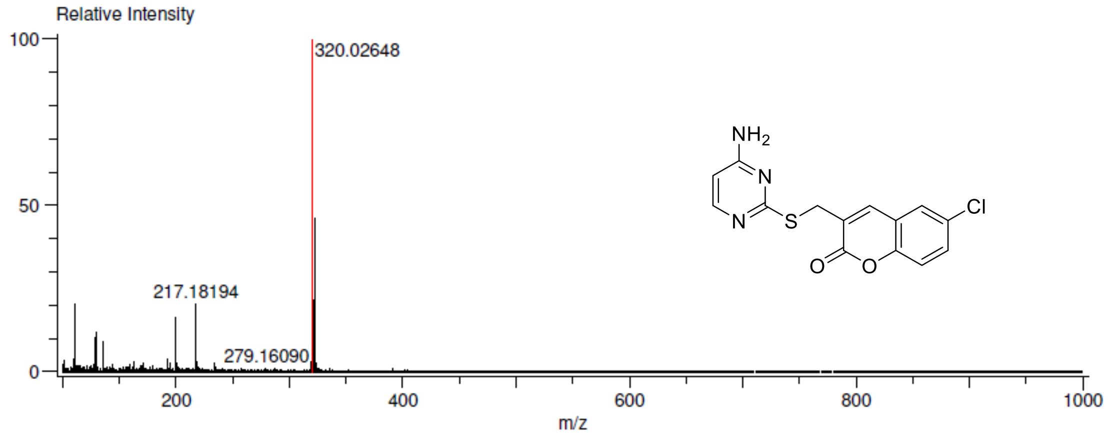

Mass	Intensity	Calc. Mass	Mass Difference [mDa]	Mass Difference [ppm]	Possible Formula
320.02648	1970681.01	320.02605	0.43	1.34	$^{12}C_{14}{}^{1}H_{11}{}^{35}Cl_1{}^{14}N_3{}^{16}O_2{}^{32}S_1$

Mass spectrum of compound **20**

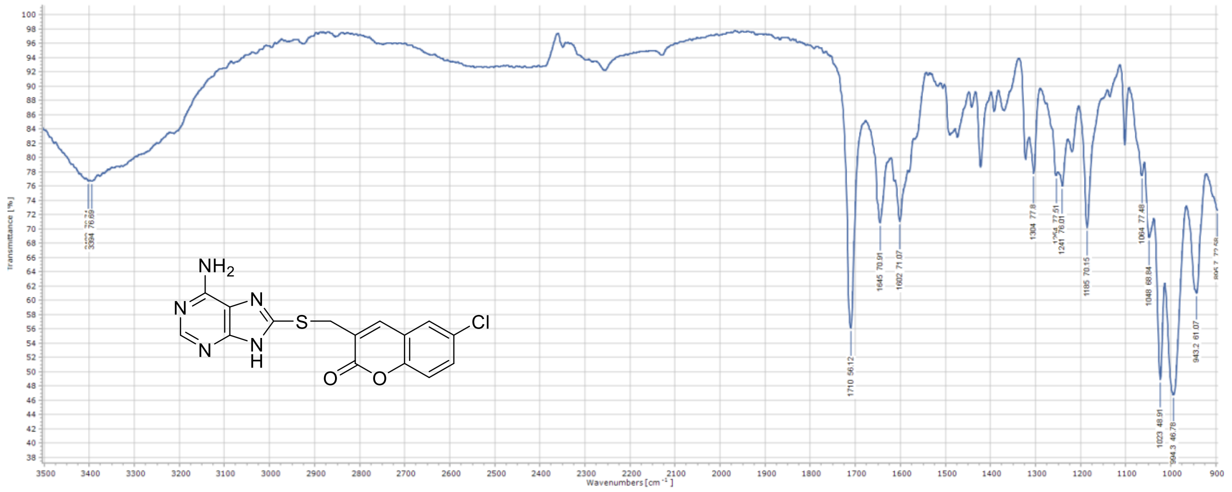

IR spectrum of compound **3a**

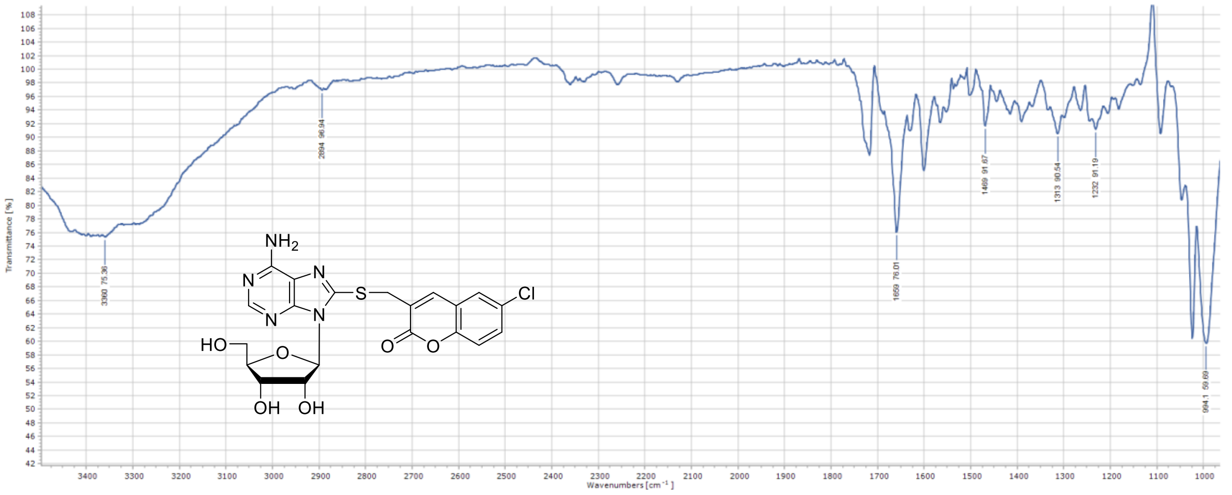

IR spectrum of compound **3b**

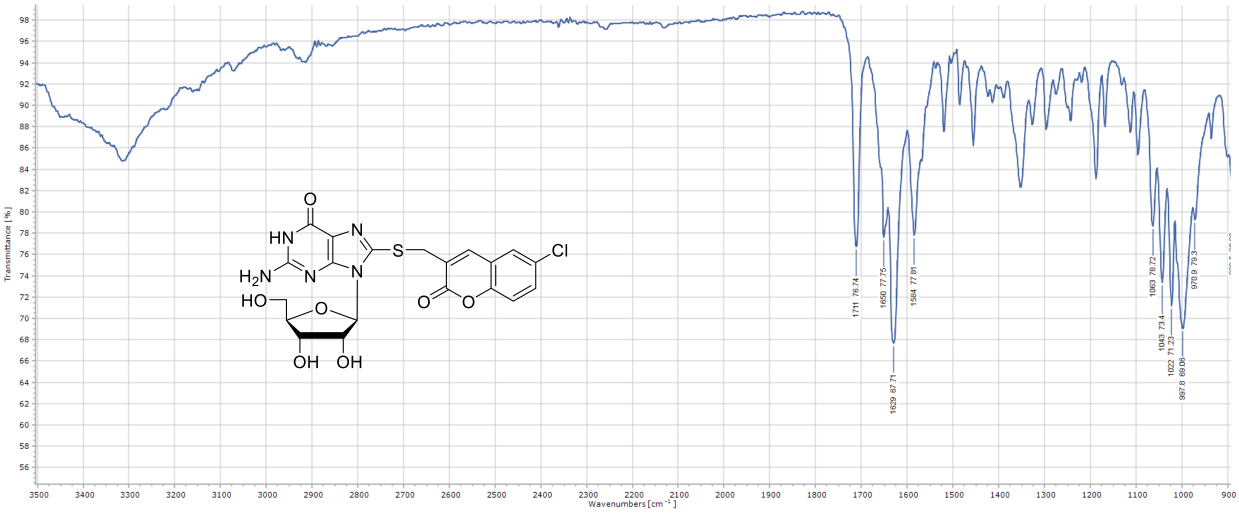

IR spectrum of compound **5a**

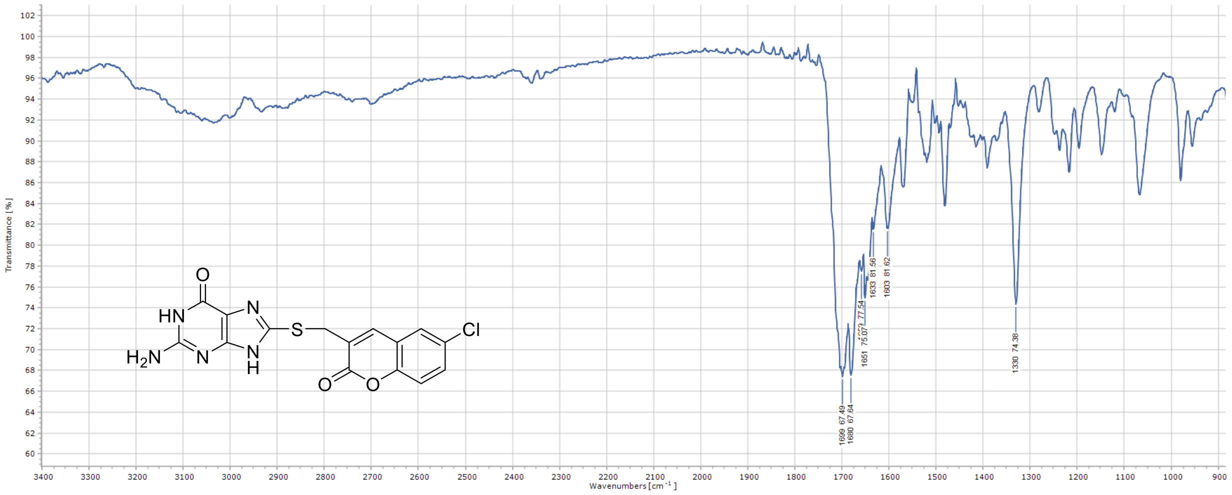

IR spectrum of compound **5b**

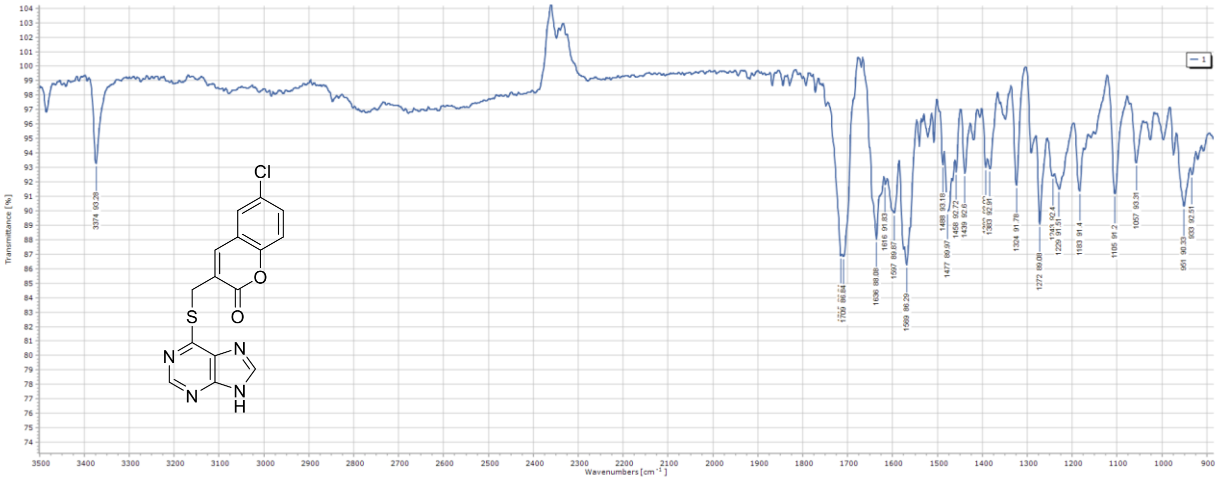

IR spectrum of compound **7a**

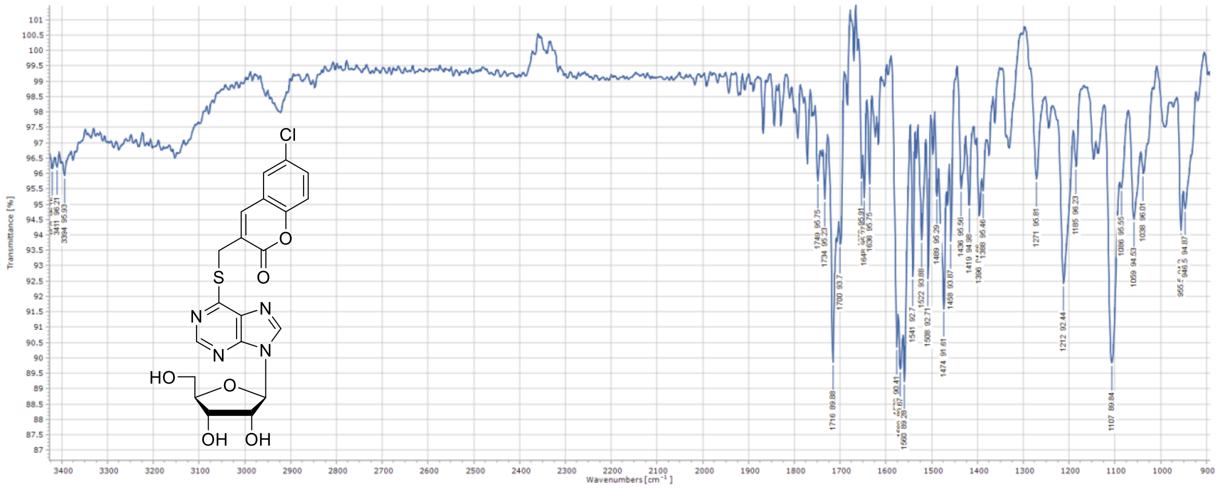

IR spectrum of compound **7b**

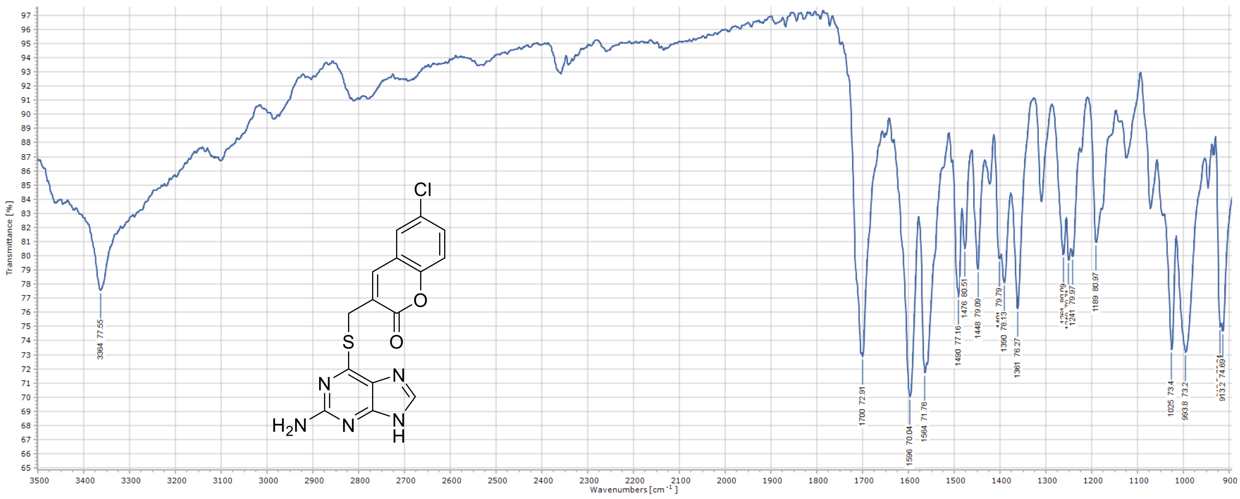

IR spectrum of compound **9a**

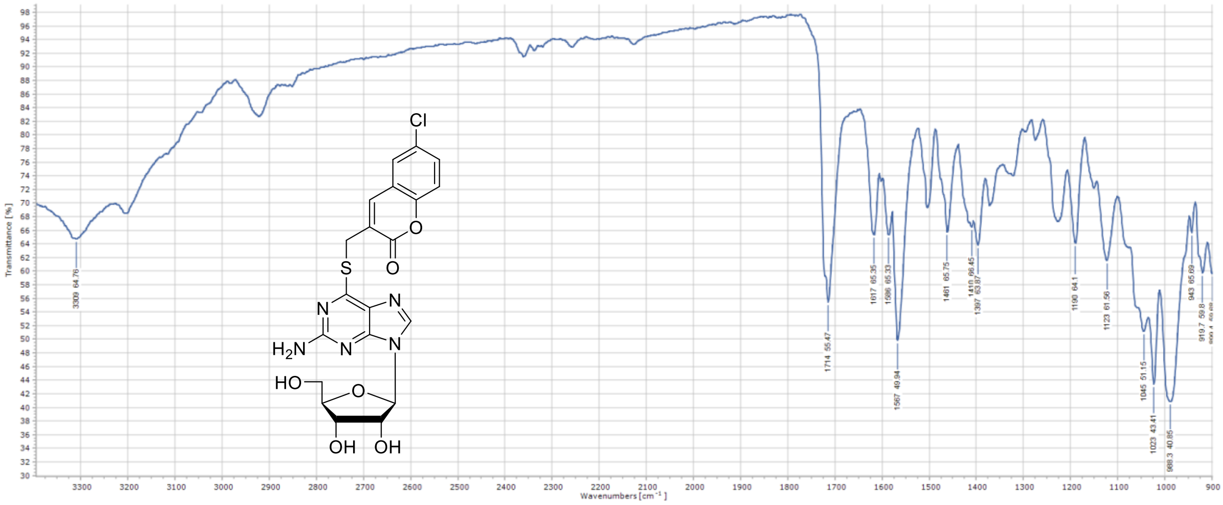

IR spectrum of compound **9b**

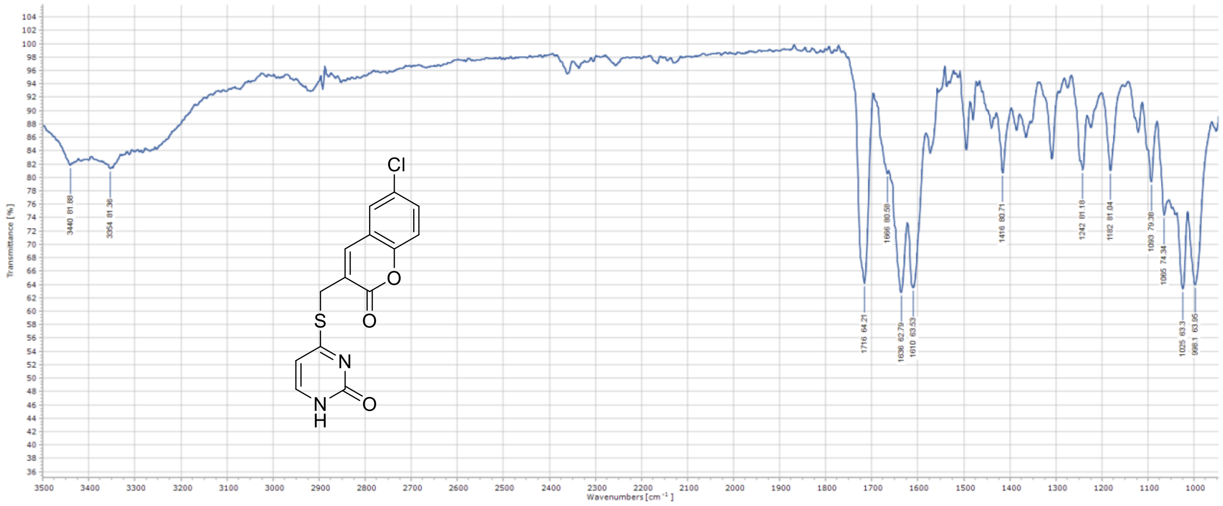

IR spectrum of compound **11a**

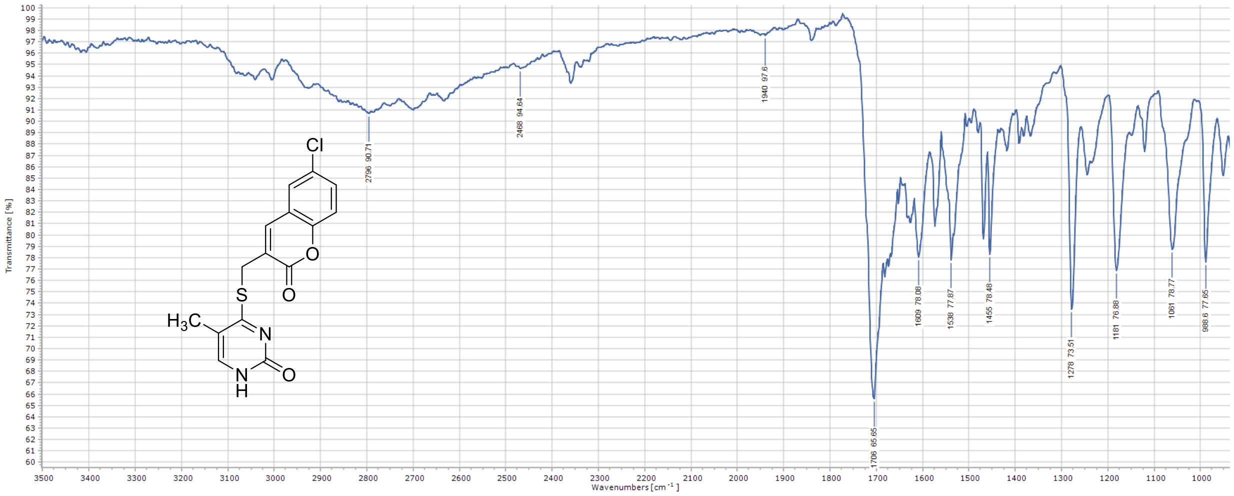

IR spectrum of compound **11b**

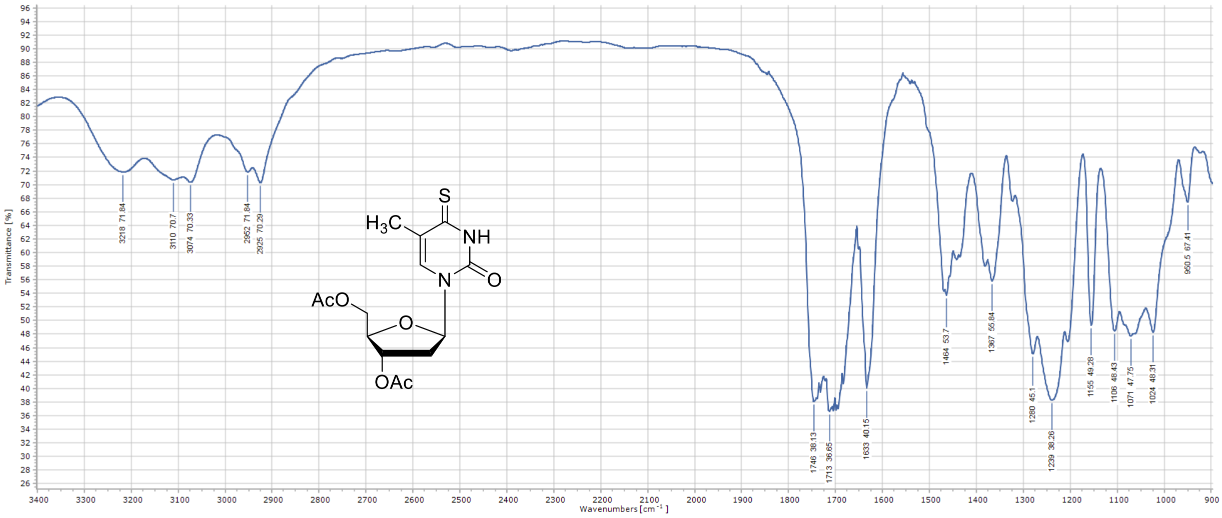

IR spectrum of compound **14b**

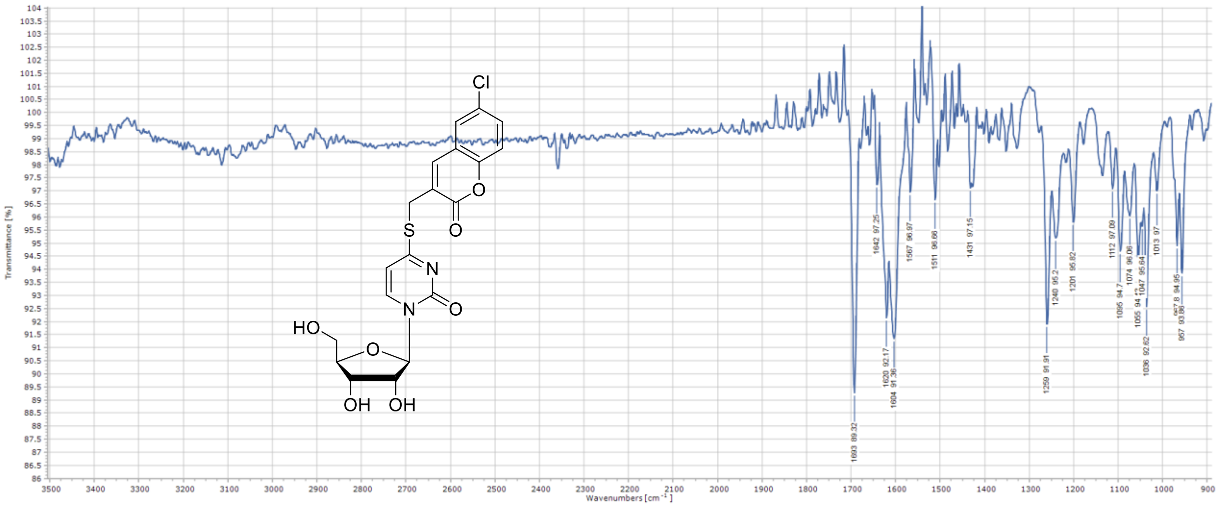

IR spectrum of compound **16a**

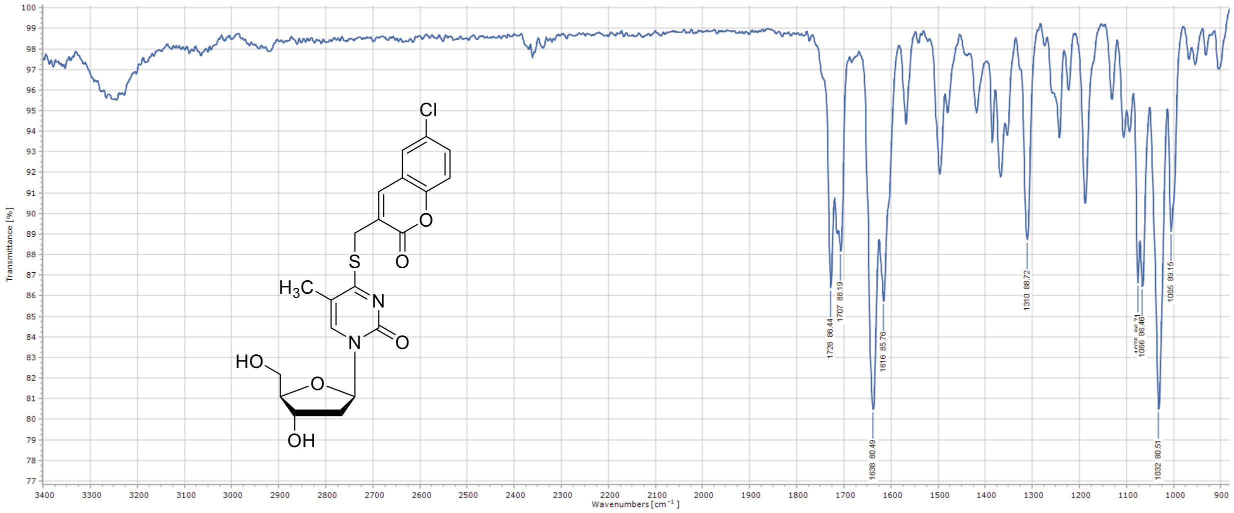

IR spectrum of compound **16b**

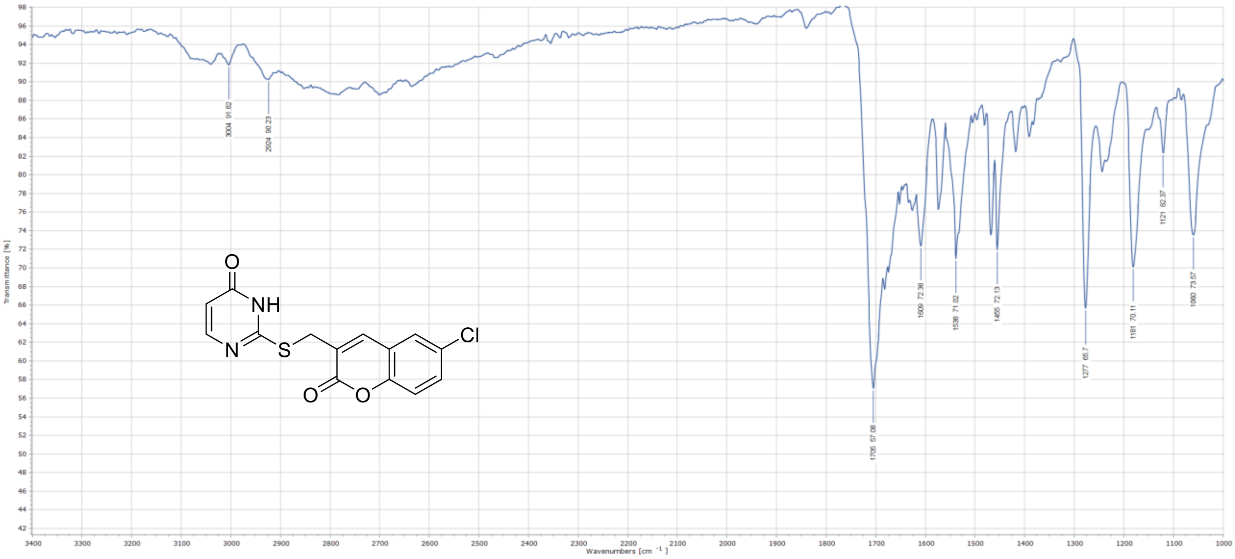

IR spectrum of compound **18**

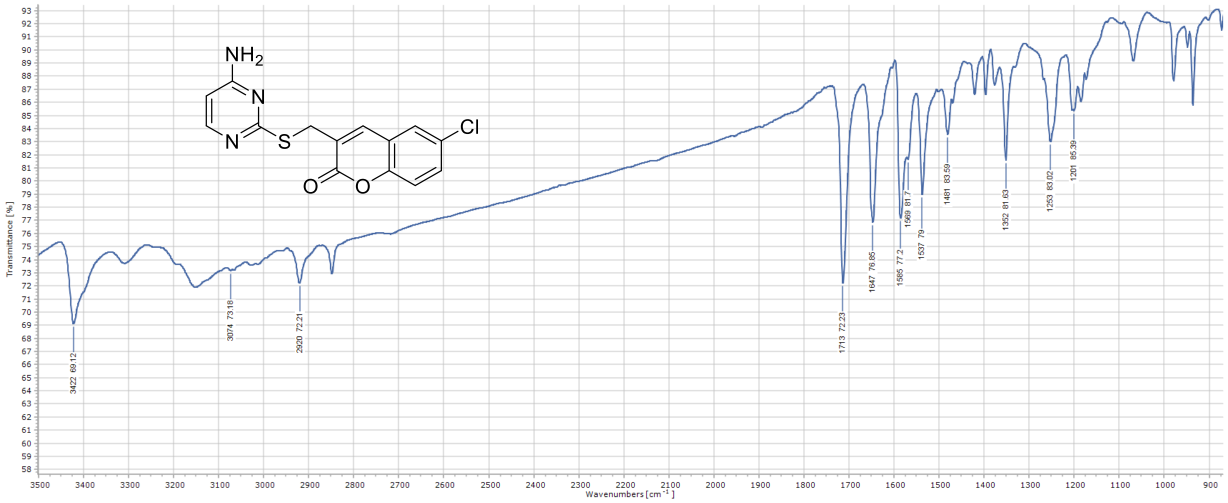

IR spectrum of compound **20**

Dose-Response Curve in Huh 5-2 Cells

In the graph below, a single Y-axis on the left side is used to represent both data sets. The **green curve** depicts cell metabolism (%) and is derived from the cell control (CC), which is the value obtained from untreated, uninfected host cells and represents 100% on the Y-axis. The green curve values were calculated using the formula:

value% = ((value – BG)/(CC – BG)) × 100, (where BG represents the background.)

The **red curve** represents the antiviral effect rate. The value obtained for infected, untreated host cells is referred to as the virus control (VC) and is depicted as 0% antiviral effect on the Y-axis. The VC, together with the CC, is used to convert all other values into a percentage of controls using the following formula:

value% = ((value – VC)/(CC – VC)) × 100.

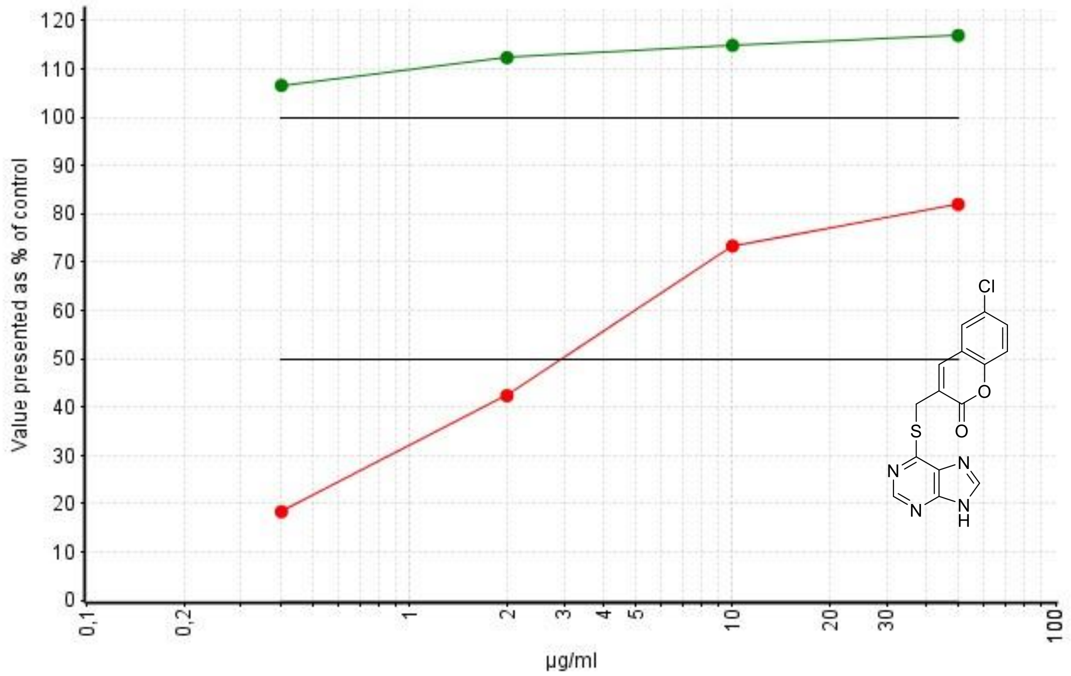

Dose-Response Curve of **7a** (maximum dose: 50 µg/ml)

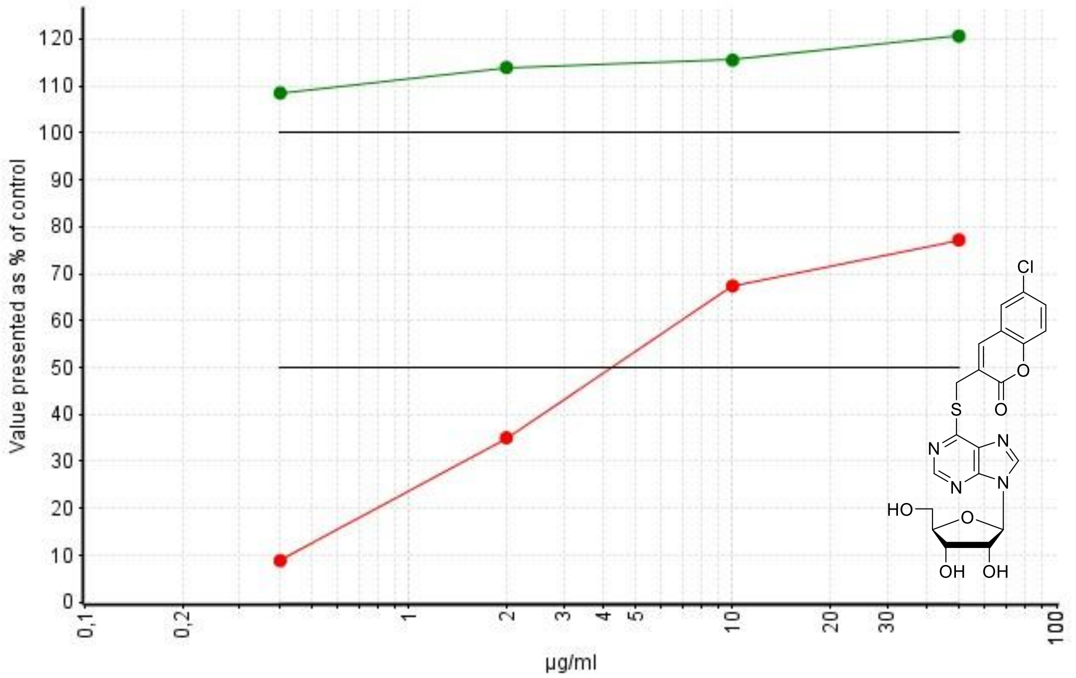

Dose-Response Curve of **7b** (maximum dose: 50 µg/ml)

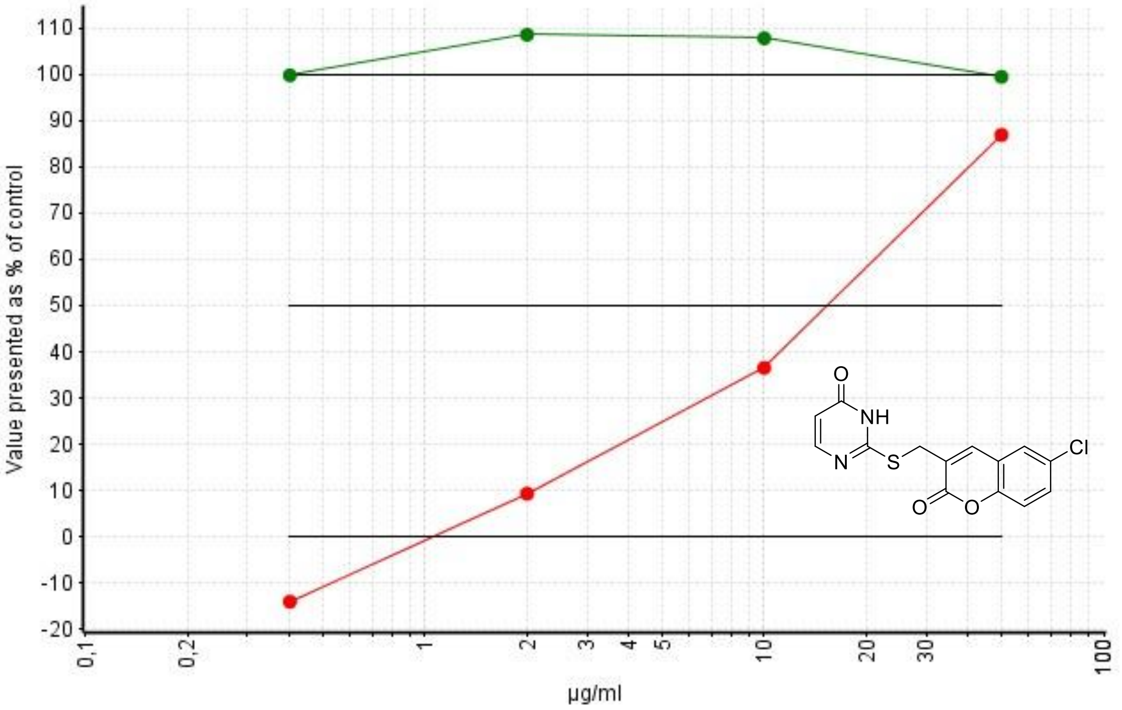

Dose-Response Curve of **18** (maximum dose: 50 µg/ml)

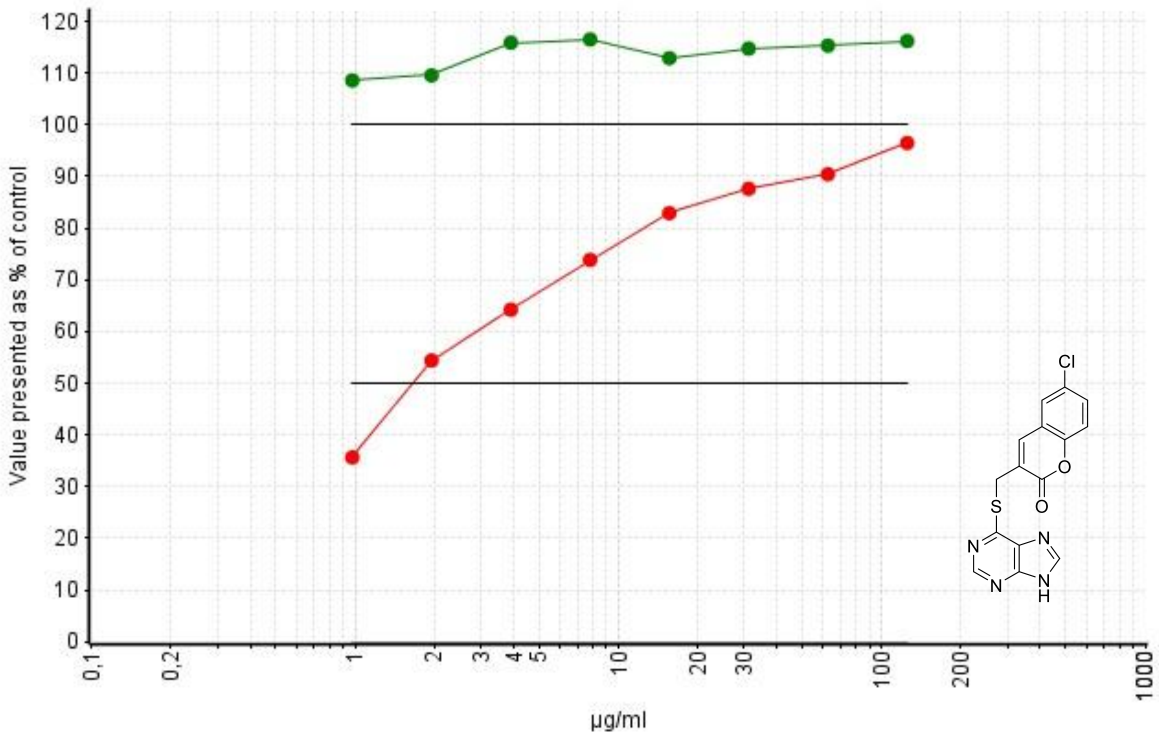

Dose-Response Curve of **7a** (maximum dose: 125 µg/ml)

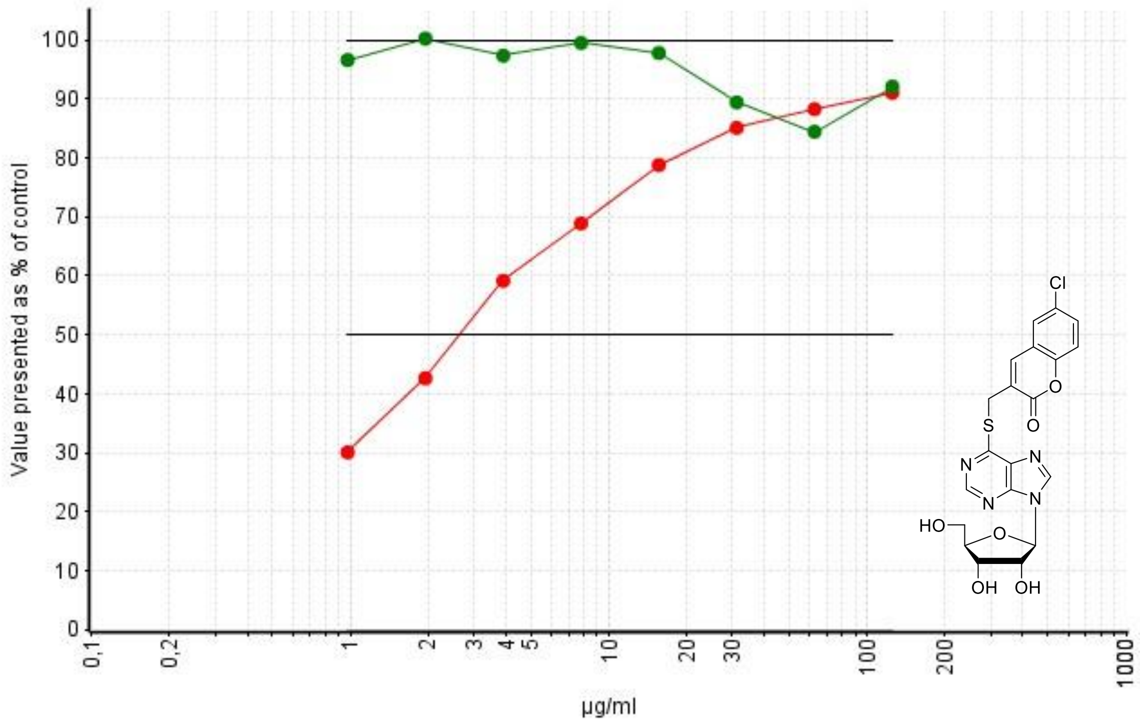

Dose-Response Curve of **7b** (maximum dose: 125 µg/ml)

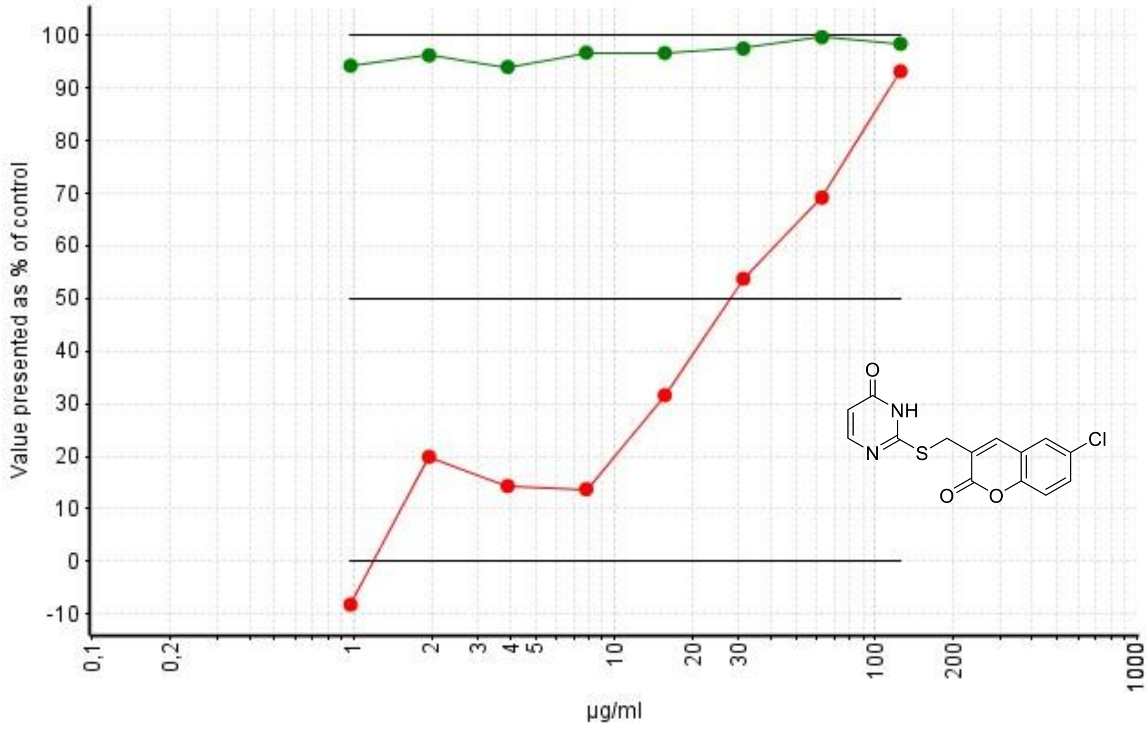

Dose-Response Curve of **18** (maximum dose: 125 µg/ml)

Dose-Response Data in Huh 9-13 Cells

Sample Name	Concentration	Quantity	% replicon RNA*	EC_{50}	% cell metabolism	CC_{50}
**7a**	50 µg/ml	4.54E-06	4	2.5 µg/ml	92	> 50 µg/ml
	16.67	1.20E-05	10		115	
	5.56	2.70E-05	22		112	
	1.852	7.41E-05	61		109	
	0.617	8.96E-05	74		105	
	0.206	1.36E-04	112		109	
	0.069	1.44E-04	119		104	
	0.023	1.12E-04	93		109	
**7b**	50 µg/ml	3.63E-06	3	3.17 µg/ml	94	> 50 µg/ml
	16.67	1.64E-05	14		113	
	5.56	2.85E-05	24		115	
	1.85	9.12E-05	75		106	
	0.617	1.03E-04	85		108	
	0.206	2.07E-04	171		102	
	0.069	1.33E-04	110		107	
	0.023	1.11E-04	92		100	

**18**	125 µg/ml	4.20E-05	1	3 µg/ml	104	> 125 µg/ml
	62.50	6.77E-05	2		99	
	31.25	2.30E-04	7		103	
	15.625	4.59E-04	14		98	
	7.813	1.12E-03	35		101	
	3.906	1.40E-03	44		109	
	1.953	2.06E-03	64		92	
	0.977	2.11E-03	66		103	

* After collection of all samples, RNA was extracted and samples were analyzed by quantitative real-time PCR for their HCV replicon content. Real-time RT-PCR values of all assayed samples were normalized against the "no-drug control".
